# Quantitative analysis of the grain amyloplast proteome reveals differences in metabolism between two wheat cultivars at two stages of grain development

**DOI:** 10.1186/s12864-018-5174-z

**Published:** 2018-10-24

**Authors:** Dongyun Ma, Xin Huang, Junfeng Hou, Ying Ma, Qiaoxia Han, Gege Hou, Chenyang Wang, Tiancai Guo

**Affiliations:** 1 College of Agronomy/National Engineering Research Center for Wheat, Henan Agricultural University, Zhengzhou, 450002 China; 2 The National Key Laboratory of Wheat and Maize Crop Science, Henan Agricultural University, Zhengzhou, 450002 China

**Keywords:** Wheat, Amyloplast, Proteome, Grain development, Starch characteristics

## Abstract

**Background:**

Wheat (*Triticum aestivum* L.) is one of the world’s most important grain crops. The amyloplast, a specialized organelle, is the major site for starch synthesis and storage in wheat grain. Understanding the metabolism in amyloplast during grain development in wheat cultivars with different quality traits will provide useful information for potential yield and quality improvement.

**Results:**

Two wheat cultivars, ZM366 and YM49–198 that differ in kernel hardness and starch characteristics, were used to examine the metabolic changes in amyloplasts at 10 and 15 days after anthesis (DAA) using label-free-based proteome analysis. We identified 523 differentially expressed proteins (DEPs) between 10 DAA and 15 DAA, and 229 DEPs between ZM366 and YM49–198. These DEPs mainly participate in eight biochemical processes: carbohydrate metabolism, nitrogen metabolism, stress/defense, transport, energetics-related, signal transduction, protein synthesis/assembly/degradation, and nucleic acid-related processes. Among these proteins, the DEPs showing higher expression levels at 10 DAA are mainly involved in carbohydrate metabolism, stress/defense, and nucleic acid related processes, whereas DEPs with higher expression levels at 15 DAA are mainly carbohydrate metabolism, energetics-related, and transport-related proteins. Among the DEPs between the two cultivars, ZM366 had more up-regulated proteins than YM49–198, and these are mainly involved in carbohydrate metabolism, nucleic acid-related processes, and transport.

**Conclusions:**

The results of our study indicate that wheat grain amyloplast has the broad metabolic capability. The DEPs involved in carbohydrate metabolism, nucleic acids, stress/defense, and transport processes, with grain development and cultivar differences, are possibly responsible for different grain characteristics, especially with respect to yield and quality-related traits.

**Electronic supplementary material:**

The online version of this article (10.1186/s12864-018-5174-z) contains supplementary material, which is available to authorized users.

## Background

Wheat (*Triticum aestivum* L.) is one of the most important grain crops in the world and is a staple food for almost one-third of the human population. Wheat flour is used in a wide range of applications including bread, pasta, steamed bread, noodles, pastries, and cookies. Starch endosperm accounts for > 80% of the volume of the wheat kernel [1]. Therefore, wheat kernel weight is largely determined by starch biosynthesis and storage, and improving starch biosynthesis could increase starch accumulation and kernel weight [2–4]. Starch consists of two major components; amylose, a linear α-1,4 linked D-glucose polymer, and amylopectin, a branched α-1,4 and α-1,6 D-glucose polymer. In the starch granule, it is widely acknowledged that starch is deposited as B-type granules (diameter <  9.9 μm), and A-type granules (diameter >  9.9 μm) in mature wheat grains [5, 6]. Starch components and the granule size distribution strongly influence starch physicochemical properties and the functionality of wheat flours [7–9].

Amyloplasts are a specialized type of leucoplast that serves as the major site of synthesis and long-term storage of starch in the endosperm [10]. Many starch granules are formed and developed within a single amyloplast, and the amyloplast envelope begins to degrade after the amyloplast becomes full of starch granules [11]. The number of plastid DNA copies per amyloplast increases from ~ 10 copies at 9 days after anthesis (DAA) to ~ 50 copies in the mature amyloplast at 31 DAA [12]. There are functional connections between starch biosynthesis and the structure of internal amyloplast membranes [13]. The amyloplast membrane protein SSG6, encodes a protein homologous to aminotransferase, and is a novel protein controlling starch grain size [14]. A previous study in rice also showed that one or more biochemical processes in the amyloplast stroma control carbon flux into starch [15]. By analogy with chloroplasts and the related non-photosynthetic etioplasts, amyloplasts should have broad metabolic capabilities [16–18]. Balmer et al. [19, 20] and Andon et al. [21] have suggested that amyloplasts are involved in many metabolic functions in addition to starch production. The results of proteomics studies also showed that amyloplasts play a central role in endosperm metabolism, and that the amyloplast may possess a regulatory mechanism that mediates the effect of interaction between genes and environment on protein and starch production [22]. In addition to starch, some important factors (timing, duration, and rate of grain filling) that determine the final protein yield are largely controlled by the amyloplasts present in the endosperm [21]. Amyloplasts also play a role in kernel hardness, and lipids associated with the starch granule surface mainly originate from the amyloplast bilayer lipid membrane which is degraded during seed desiccation [23].

Grain filling is an important process for kernel development, grain compound accumulation, and for determining the final kernel weight and flour quality. Proteomic approaches have been widely used to identify the array of proteins present in the developing wheat grain [24, 25]. Apart from the whole grain, some studies have also focused on isolated organelles or tissues, such as nuclei [26], wheat aleurone layer [27], and kernel periphery [28]. These studies have provided valuable information about the biochemical processes involved in wheat grain development. Proteomics have also been used to characterize wheat amyloplast proteins [21], and the results showed that amyloplasts in the developing wheat endosperm have broad metabolic capability [19, 21].

However, as mentioned above, different wheat types (such as hard vs. soft), and different grain developmental stages have different physiological processes. Previous reports have reviewed the metabolic processes that occur in the wheat amyloplast, but there is little information available about amyloplasts from different wheat cultivars at different stages of grain filling. The label-free-based quantitative proteome method allows for sensitive and accurate protein quantification. In this study, two wheat cultivars with different kernel hardness and starch characteristics were used to study the differentially expressed amyloplast proteins during grain development using the label-free technique. Our findings will provide insights into the function of wheat amyloplast during grain development.

## Results

### Comparing grain characteristics in the two wheat cultivars

As shown in Table 1, hard wheat cultivar ZM366 had higher protein content, while the soft wheat cultivar YM49 had higher kernel weight. Examination of starch granules at maturity showed that ZM366 had a relatively larger number of small granules in the volume and surface distribution, whereas YM49 had a relatively higher number of larger granules.

**Table 1:** Grain characteristics of the two wheat cultivars ZM366 (hard) and YM49–198 (soft)

Items			ZM366	YM49–198
Hardness index (H)			77 ± 0.19	33 ± 1.81
Thousand kernel weight (g)			43.60 ± 0.73	45.80 ± 1.27
Kernel length (cm)			0.579 ± 0.011	0.600 ± 0.028
Kernel width (cm)			0.325 ± 0.005	0.325 ± 0.010
Kernel thick (cm)			0.293 ± 0.013	0.288 ± 0.014
Protein content (mg g^−1^)			144.0 ± 1.6	136.4 ± 1.0
Starch content (mg g^−1^)			776.8 ± 3.6	797.2 ± 5.4
Starch granule size distribution	Volume distribution (%)	< 2.0 μm	11.17 ± 0.28	9.50 ± 0.84
		< 9.8 μm	43.67 ± 1.00	38.33 ± 3.50
		> 9.8 μm	56.33 ± 1.00	61.67 ± 3.50
	Surface distribution (%)	< 2.0 μm	54.71 ± 0.98	51.65 ± 2.34
		< 9.8 μm	88.14 ± 2.30	86.33 ± 1.40
		> 9.8 μm	11.86 ± 2.30	13.67 ± 1.40
	Number distribution (%)	< 2.0 μm	97.13 ± 1.59	96.49 ± 1.42
		< 9.8 μm	99.92 ± 0.02	99.89 ± 0.03
		> 9.8 μm	0.08 ± 0.02	0.11 ± 0.03
Crystallinity of starch granule (%)			13.79 ± 0.75	12.99 ± 1.12

Data represent mean ± standard deviation (SD)

**Fig. 1:**
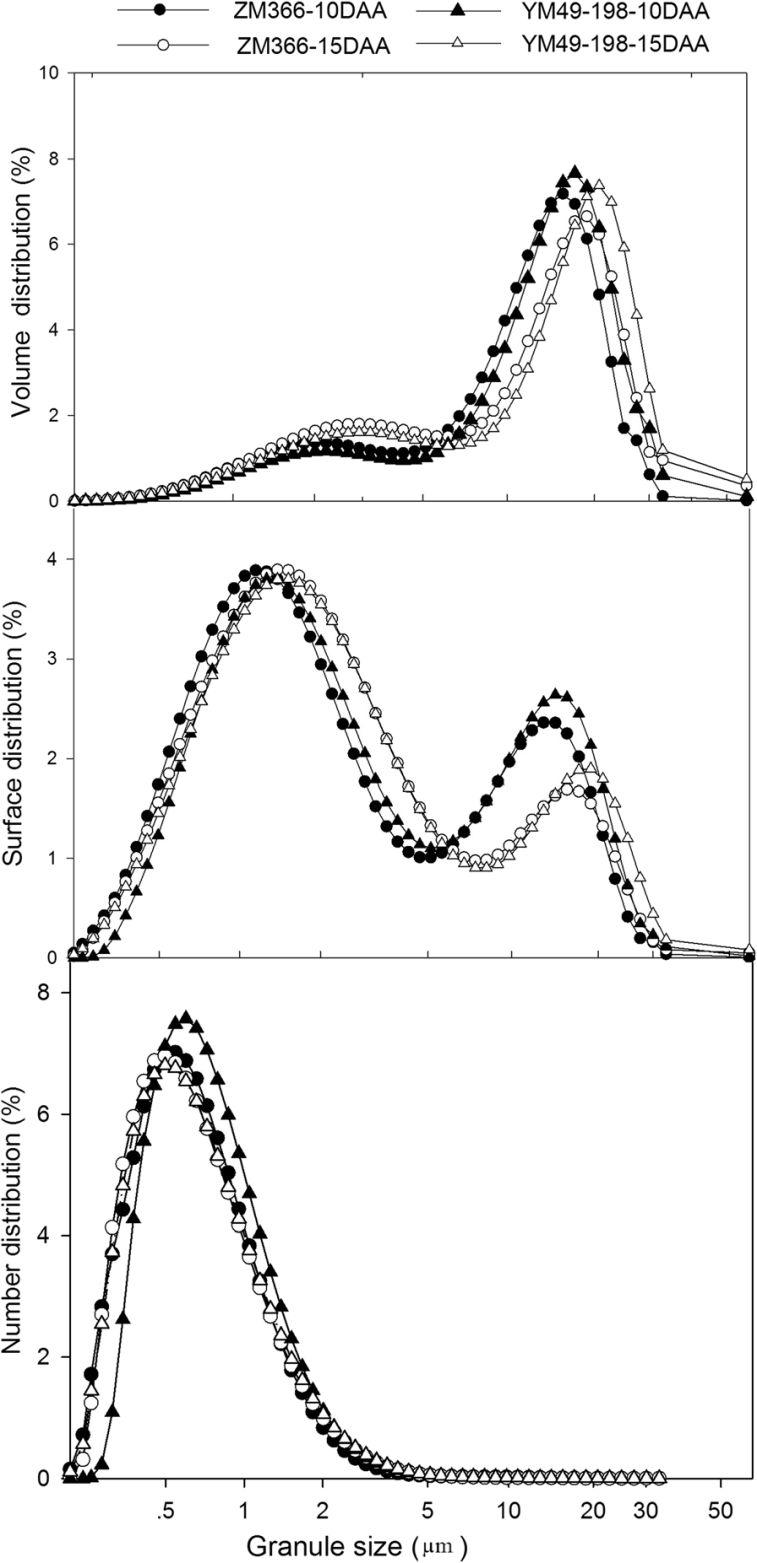
Starch granule size distribution of two wheat cultivars at 10 DAA and 15 DAA

The starch particle distribution in the grain at 10 DAA and 15 DAA is shown in Fig. 1, and it is clear that the volume distribution peak shifted from 15.65–17.18 μm at 10 DAA to 18.86–20.71 μm at 15 DAA. The average volume distribution of granules < 5.1 μm was 23.33% and 29.28% at 10 DAA and 15 DAA, respectively. For granule sizes between 5.1 and 9.8 μm, the average volume percent was 16.42% and 11.58% at 10 DAA and 15 DAA, respectively. The volume distribution of sizes < 5.1 μm and 5.1–9.8 μm for hard wheat cultivar ZM366 was 28.40% and 15.40%, respectively, and it was 24.21% and 12.60%, respectively, for soft wheat cultivar YM49–198. There are two peaks for granule surface distribution; one was at 1.15–1.38 μm, and the other was at 15.65–18.86 μm. The average surface distribution percentages at 10 DAA for the granule size ranges 1.15–5.1 μm, 5.1–18.86 μm, and >  9.8 μm were 42.07%, 27.35%, and 19.63%, respectively. Correspondingly, the average percentages at 15 DAA were 45.60%, 19.37%, and 15.79%, respectively. The surface distribution percentage of the hard wheat cultivar ZM366 for granules < 9.8 μm was 83.85%, while for soft wheat cultivar YM49–198 it was 80.73%. For number distribution, the average percentages for granules < 0.6 μm and <  9.8 μm were 47.51% and 99.88% at 10 DAA, respectively. The corresponding number distributions at 15 DAA were 52.69% and 99.92%, respectively. For hard wheat cultivar ZM366, the number distribution percentages for granules < 0.6 μm and <  9.8 μm were 52.97% and 99.91%, respectively, and the number distribution percentages for soft wheat cultivar YM49–198 were 47.23% and 99.89%, respectively. These results indicate that wheat grains at 10 DAA had more large-size particles than at 15 DAA, and that the hard wheat had more small particles than did the soft wheat.

### Identification of differentially accumulated proteins at different grain development stages

**Fig. 2:**
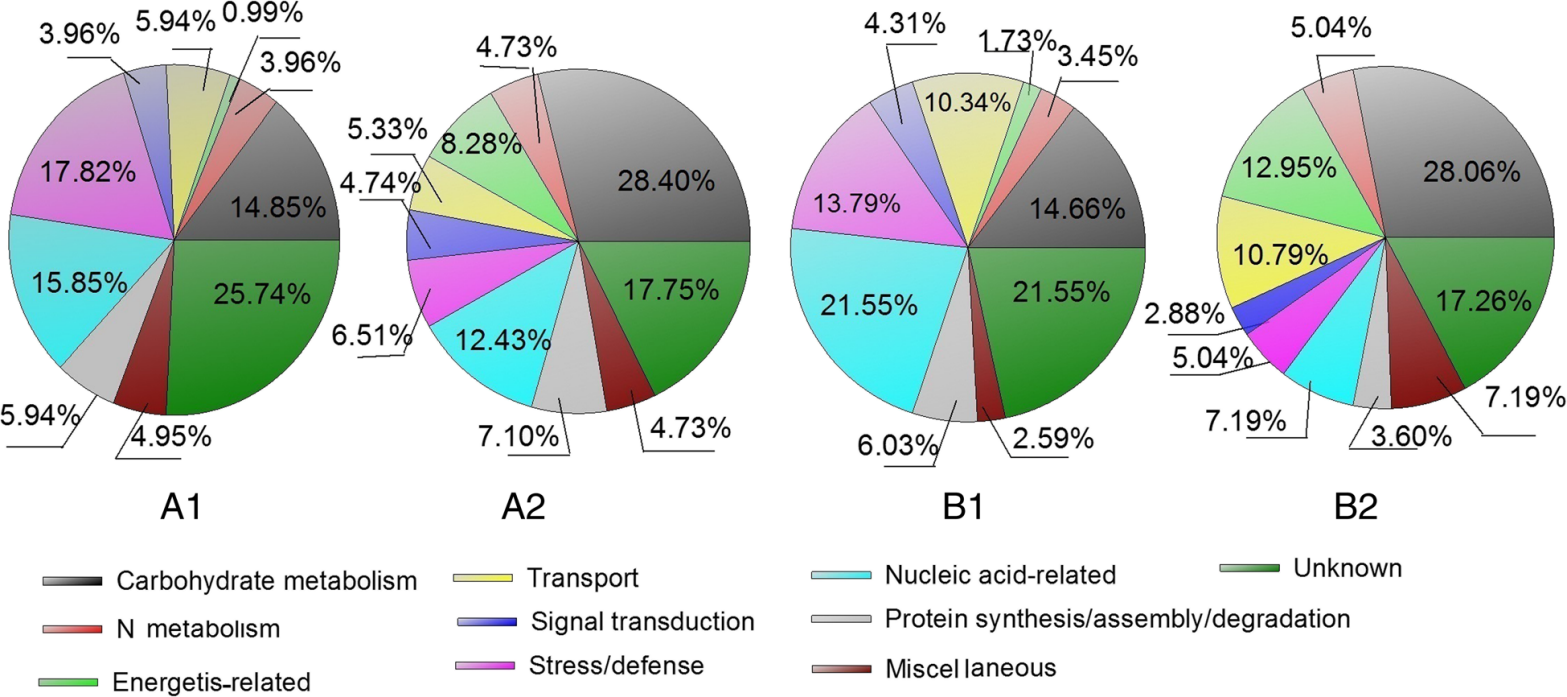
Function classifications of DEPs identified in wheat grain of cultivar ZM366 (A1, A2), and YM49–198 (B1, B2) at two grain developmental stages. A1, Up-regulation DEPs of wheat cultivar ZM366 from 10DAA to 15DAA; A2, Down-regulation DEPs of wheat cultivar ZM366 from 10DAA to 15DAA; B1, Up-regulation DEPs of wheat cultivar YM49–198 from 10DAA to 15DAA; B2, Down-regulation DEPs of wheat cultivar YM49–198 from 10DAA to 15DAA

In this study, we used label-free-based quantitative proteome characterization of hard and soft wheat cultivars to investigate the different metabolic proteins present in wheat amyloplasts at different developmental stages. A fold change of ≥2 and *p* ≤ 0.05 were used as thresholds to indicate significant changes in the abundance of differentially expressed proteins (DEPs) during grain development. Of 1104 non-redundant proteins identified, 270 showed > 2-fold change (*p* ≤ 0.05) in relative protein expression levels in ZM366 from 10 DAA to 15 DAA; of 961 non-redundant proteins identified in YM49–198, 253 showed > 2-fold change (*p* ≤ 0.05) in protein expression levels (Additional file 1: Table S1; Additional file 2: Table S2). Compared with the corresponding proteins expressed at 10 DAA, the number of proteins showing increased levels at 15 DAA were 101 (37.41%) and 116 (45.85%) for ZM366 and YM49–198, respectively. Based on the molecular functions given on the UniProt and Gene Ontology websites, the DEPs were classified into ten functional categories (Fig. 2); carbohydrate metabolism (14.66–28.40%), N metabolism (3.45–5.04%), energetics related (0.99–12.95%), transport (4.74–10.79%), signal transduction (2.88–4.74%), stress/defense (45.04–17.82%), nucleic acid-related (7.19–21.55%), protein synthesis/assembly/degradation (3.60–7.10%), miscellaneous (2.59–7.19%), and unknown (17.26–25.74%).

**Fig. 3:**
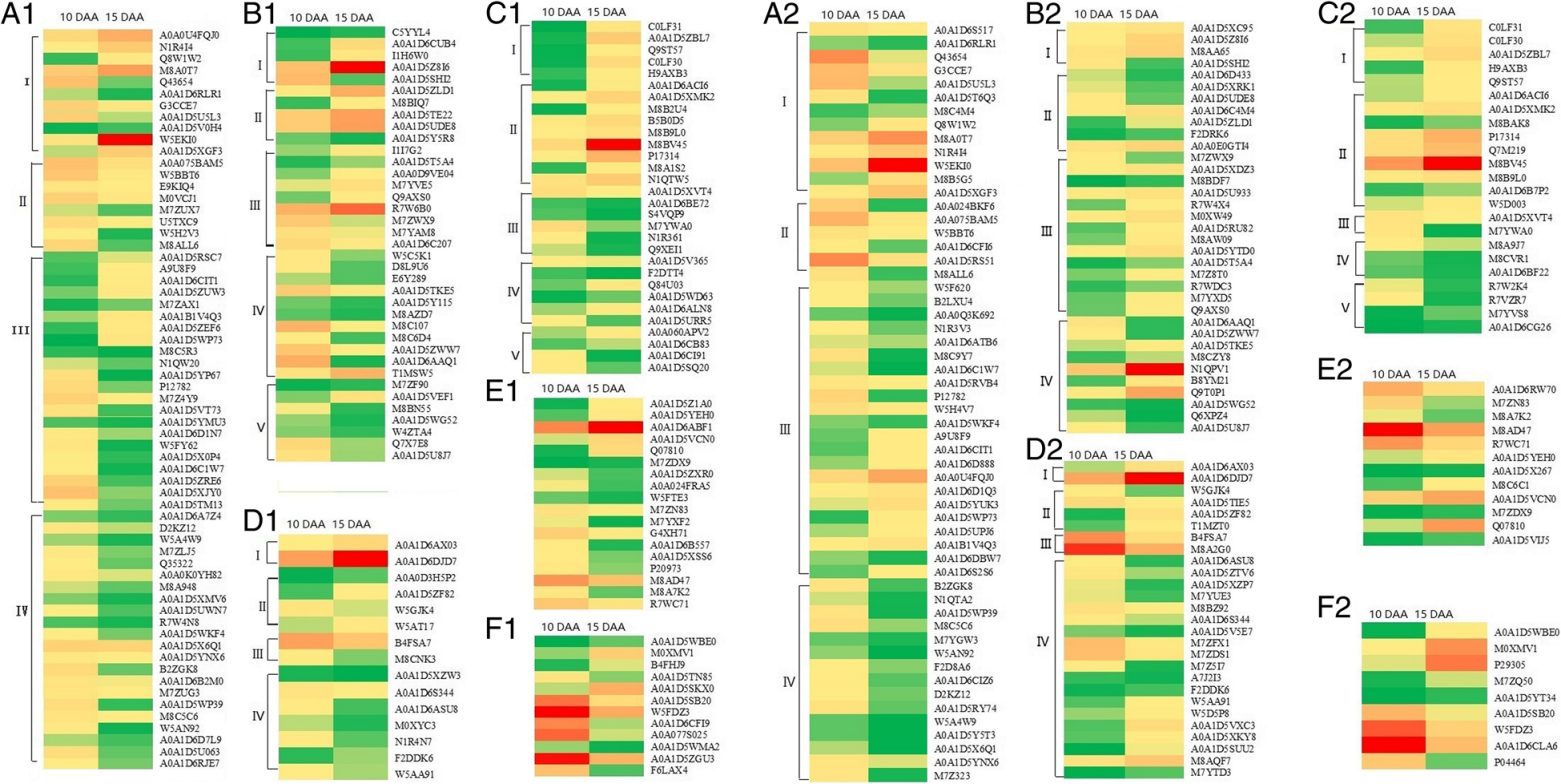
Hierarchical clustering analysis of DEPs identified in grain amyloplast during grain development. A1, A2: DEPs of ZM366 (A1) and YM49–198 (A2) involved in carbohydrate metabolism related proteins, including starch metabolism (A1-I, A2-I), photosynthesis (A1-II, A2-II), glycolysis (A1-III, A2-III), and other carbohydrate metabolism (A1-IV, A2-IV). B1, B2: DEPs of ZM366 (B1) and YM49–198 (B2) involved in nucleic acid-related proteins, including histone (B1-I, B2-I), RNA binding (B1-II, B2-II), 40S/60S ribosomal protein (B1-III, B2-III), translation initiation/elongation factor (B1-IV, B2-IV), and other nucleic acid-related proteins (B1-V, B2-V). C1, C2: DEPs of ZM366 (C1) and YM49–198 (C2) involved in stress/defense proteins, including serpin (C1-I, C2-I), serine-type endospeptidase/alpha-amylase/trypsin inhibitor (C1-II, C2-II), chaperone/heat shock protein (C1-III, C2-III), antioxidant system (C1-IV, C2-IV), and other stress/defense related proteins (C1-V, C2-V). D1, D2: DEPs of ZM366 (D1) and YM49–198 (D2) involved in transport, including ADPG brittle (D1-I, D2-I), protein transporter (D1-II, D2-II), ADP, ATP carrier (D1-III, D2-III), and others transport proteins (D1-IV, D2-IV). E1, E2: DEPs of ZM366 (E1) and YM49–198 (E2) involved in protein synthesis/assembly/degradation. F1, F2: DEPs of ZM366 (F1) and YM49–198 (F2) involved in signal transduction. Red color, the higher abundance of protein expression; Green color, the lower abundance of protein expression

As shown in Fig. 3 and Additional file 2: Table S2, the 63 ZM366 DEPs involved in carbohydrate metabolism were divided into four functional groups (Fig. 3, A1); starch metabolism (A1-I), photosynthesis (A1-II), glycolysis (A1-III), and other carbohydrate metabolism (A1-IV). In group A1-I, there were 11 enzymes involved in starch biosynthesis, of which seven were up-regulated and four were down-regulated including one starch synthesis (Q43654) and two starch branching enzymes (A0A1D6RLR1 and G3CCE7), and one glucan branching enzyme (A0A1D5U5L3). In group A1-II, eight DEPs involved in photosynthesis were all down-regulated from 10 DAA to 15 DAA. In group A1-III, 22 DEPs were involved in glycolysis, of which eight were up-regulated. The 22 DEPs in group A1-IV were involved in other carbohydrate metabolic processes, and all were down-regulated. The 37 DEPs involved in nucleic acid-related process were divided in five groups (Fig. 3, B1), including five histone (B1-I), five RNA binding (B1-II), nine ribosomal proteins (B1-III), eleven translation initiation/elongation factors (B1-IV), and seven other nucleic acid-related proteins (B1-V). Group I contained four up-regulated proteins and one down-regulated protein (A0A1D5SH12). In group IV, all proteins except for one elongation factor (T1MSW5) were down-regulated, and the relative abundance of elongation factor Tu (A0A1D6AAQ1) decreased by almost 50-fold at 15 DAA.

There were 30 DEPs involved in stress/defense (Fig. 3, C1), and these were divided into five functional groups, including serpins (C1-I), serine-type endopeptidase/alpha-amylase inhibitors (C1-II), chaperone and heat shock proteins (C1-III), Reactive oxygen species (ROS) scavenging system (C1-IV), and other stress/defense proteins (C1-V). Group I contained five serpins (C0LF31, A0A1D5ZBL7, Q9ST57, C0LF30, and H9AXB3), and group II contained three serine-type endopeptidase inhibitors (A0A1D6ACI6, A0A1D5XMK2, and M8B2U4) and six alpha-amylase/trypsin inhibitors (B5B0D5, M8B9L0, M8BV45, P17314, M8A1S2, and N1QTW5); all of these proteins were up-regulated from 10 DAA to 15 DAA. Three chaperones (A0A1D5XVT4, A0A1D6BE72, and S4VQP9), and three heat shock proteins (M7YWA0, N1R361, and Q9XEI1) in group III were all down-regulated. The ROS scavenging system group contained six DEPs; peroxidase (Q84U03), pyrroline-5-carboxylate reductase (A0A1D5WD63), and betaine-aldehyde dehydrogenase (A0A1D6ALN8) were up-regulated, and oxophytodienoate reductase (A0A1D5URR5), L-ascorbate peroxidase (A0A1D5V365), and peroxiredoxin (F2DTT4) were down-regulated.

The 15 DEPs that participate in transport processes were divided into four groups (Fig. 3, D1), including group D1-I (two brittle proteins: A0A1D6AX03 and A0A1D6DJD7), group D1-II (four protein transporters), group D1-III (two ADP, ATP carrier proteins), and group D1-IV (seven other transportation proteins including two transmembrane proteins). Among these proteins, six were up-regulated, including two brittle proteins (A0A1D6AX03 and A0A1D6DJD7) and two protein transporters (A0A0D3H5P2 and A0A1D5ZF82), and nine were down-regulated, including two transmembrane proteins (A0A1D6ASU8 and M0XYC3), and two ADP, ATP carrier proteins (B4FSA7 and M8CNK3).

There were 18 DEPs involved in protein biosynthesis/assembly/degradation (Fig. 3, E1), of which six were up-regulated, including two metalloendopeptidases (A0A1D5Z1A0 and A0A1D5YEH0), two aspartic proteinases (A0A1D6ABF1 and A0A1D5VCN0), and 12 proteins were down-regulated, including four disulfide isomerases (A0A024FRA5, W5FTE3, M7ZN83, and M7YXF2) and two ubiquitin proteins (A0A1D5XSS6 and P20973). There were 12 DEPs that participate in signal transduction (Fig. 3, F1), of which four were up-regulated and eight were down-regulated. The down-regulated DEPs were mainly GTPases (A0A1D5SB20, W5FDZ3, A0A1D6CFI9, A0A077S025, A0A1D5WMA2, and A0A1D5ZGU3).

We also found 12 DEPs related to nitrogen metabolism, of which four were up-regulated, including one aspartate aminotransferase (A0A077S3V2) and one serine/threonine phosphatase (M7ZWM8), and eight were down-regulated, including ketol-acid reductoisomerase (A0A1D5RP81) (Additional file 1: Table S1). Fifteen DEPs were involved in energetics-related processes, of which only one protein (B3VEX2) was up-regulated and 14 were down-regulated, including eight ATP synthesis and five ATP binding proteins. In addition, there were 13 DEPs in miscellaneous groups, and the functions of another 56 DEPs were unclear.

Similar trends for DEPs in the grain between 10 DAA and 15 DAA developmental stages were also found in soft wheat cultivar YM49–198 (Fig. 3, A2-F2; Additional file 2: Table S2). There were 56 DEPs involved in carbohydrate metabolism, of which 13 were involved in starch metabolism, and seven of these were up-regulated. Six DEPs involved in photosynthesis were all up-regulated, as were 22 that participate in glycolysis and 15 involved in other carbohydrate metabolism. As in YM49–198, the 35 nucleic acid-related DEPs were divided into five groups, including four histone (B2-I), seven RNA/DNA binding proteins (B2-II), 14 ribosomal proteins (B2-III), four translation initiation/elongation factors (B2-IV), and six other nucleic acid-related proteins (B2-V). Almost all of the ribosomal proteins (92.86%) in group III were up-regulated, and two elongation factors were down-regulated, with the level of elongation factor Tu (A0A1D6AAQ1) decreasing by 25-fold. There were 23 DEPs identified that participate in stress/defense, including five serpins (C2-I), three serine-type endopeptidase inhibitors (C2-II), six alpha-amylase inhibitors (C2-II), one chaperone and one heat shock protein (C2-III), three ROS scavenging system proteins (C2-IV), and four other stress/defense proteins (C2-V). All the DEPs in groups I and II were up-regulated, of which serpin2 (C0LF31) increased by 22.08-fold. The five DEPs in groups III and IV were all down-regulated, including one super-oxidase dismutase (M8CVR1), one catalase (M8A9J7), and one peroxidase (A0A1D6BF22). These results indicate that the expression of stress/defense proteins is different at the two grain developmental stages.

There were 27 DEPs involved in transport processes (Fig. 3, D2), including two brittle proteins (D2-I), four protein transporter group proteins (D2-II), two ADP, ATP carriers (D2-III), and 19 other transporter proteins (D2-IV). Two brittle proteins (A0A1D6AX03 and A0A1D6DJD7) and two protein transporter proteins (A0A0D3H5P2 and A0A1D5ZF82) were up-regulated at 15 DAA. In group IV, eight DEPs were down-regulated, including four transmembrane proteins (A0A1D6ASU8, A0A1D5ZTV6, A0A1D5XZP7, and M7YUE3), and 11 were up-regulated, including three coatomers (W5D5P8, A0A1D5XKY8, and A0A1D5SUU2).

Twelve DEPs were involved in protein biosynthesis/assembly/degradation (Fig. 3, E2), of which seven were up-regulated, including one metalloendopeptidase (A0A1D5YEH0), two aspartic proteinases (M8C6C1 and A0A1D5VCN0), and one rRNA N-glycosidase (Q07810) which increased by 15.04-fold. Five DEPs were found to be down-regulated, including one disulfide isomerase (M7ZN83) and one ubiquitin protein (A0A1D5X267). There were nine DEPs that participate in signal transduction (Fig. 3, F2), of which five were up-regulated and four were down-regulated. The up-regulated DEPs were mainly 14–3-3 proteins (A0A1D5WBE0, M0XMV1, and P29305).

In addition, there were 11 DEPs related to nitrogen metabolism, of which four were up-regulated, including one serine/threonine phosphatase (M7ZWM8), and seven were down-regulated including one ketol-acid reductoisomerase (W5DWT9) that decreased by 11.11-fold (Additional file 2: Table S2). There were also 20 DEPs involved in energetics-related processes (Additional file 2: Table S2), of which only two (A0A1D5SCG8 and A0A1D6AHD7) were up-regulated and the other 18 were down-regulated, including three ATP synthesis and six ATP binding proteins. In addition, 13 DEPs were placed in miscellaneous groups, and the functions of 49 DEPs were unclear.

### Identification of cultivar-specific expressed proteins

**Fig. 4:**
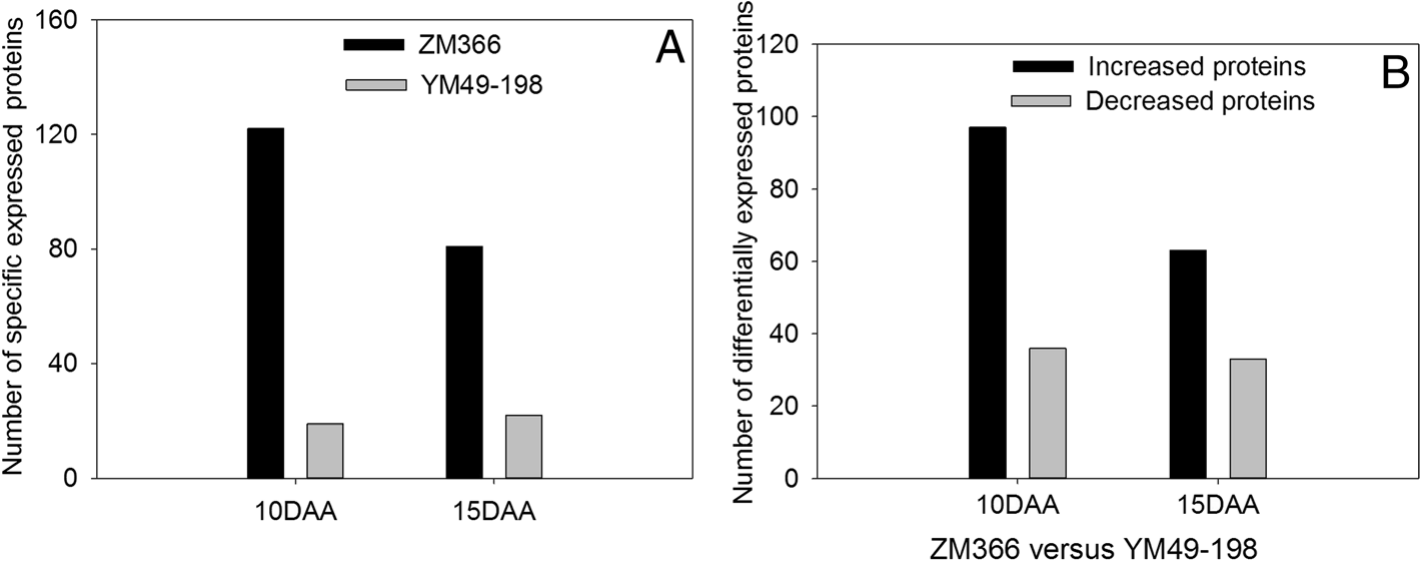
The number of cultivar-specific expressed proteins and differentially expressed proteins of wheat cultivar ZM366 and YM49–198 at 10 DAA and 15 DAA, respectively. **a**, Number of cultivar-specific expressed proteins of ZM366 and YM49–198 at 10 DAA and 15 DAA. **b**, Number of differentially expressed proteins of ZM366 and YM49–198 at 10 DAA and 15 DAA

**Fig. 5:**
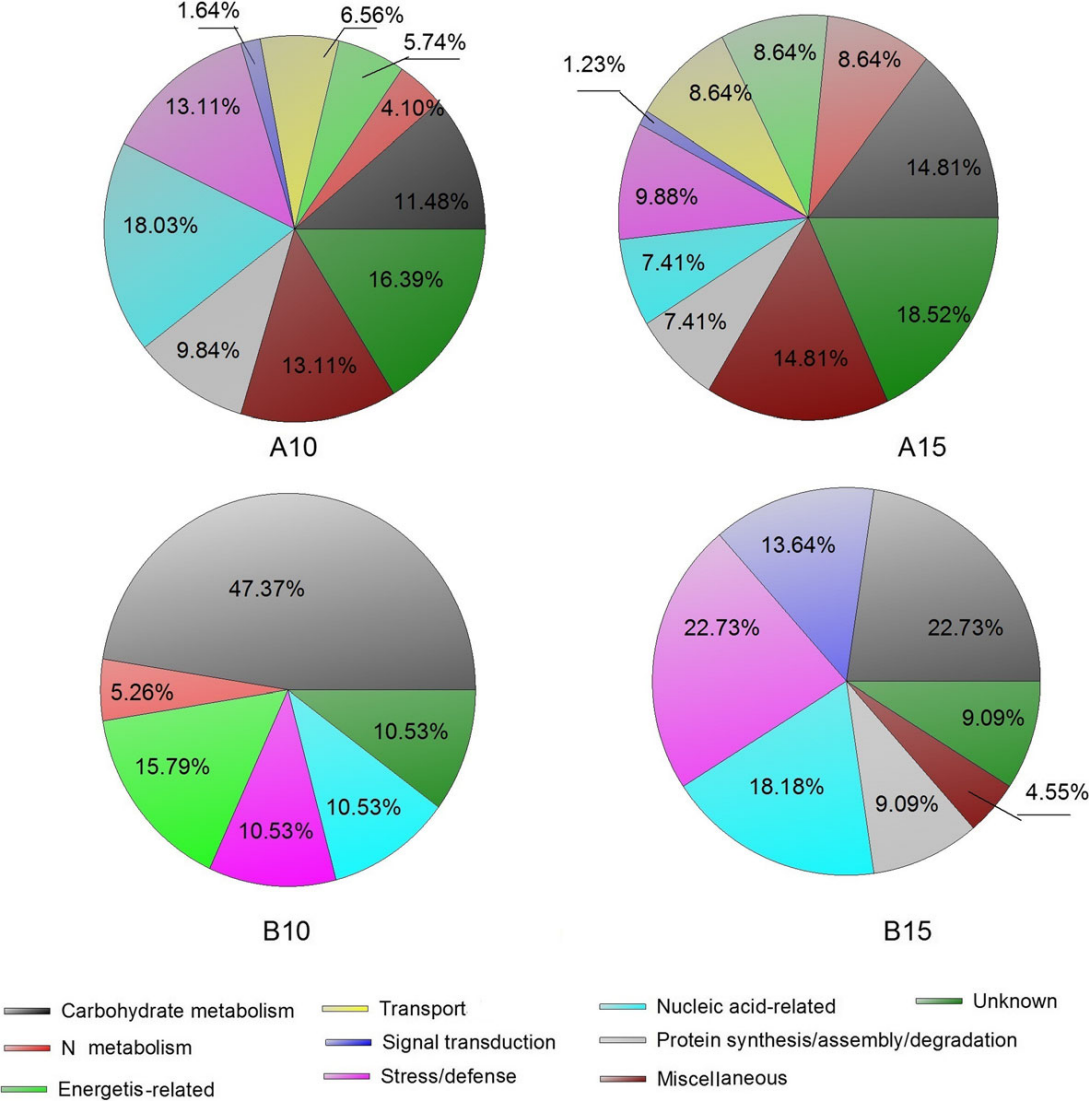
Function classification of cultivar-specific expressed proteins identified in wheat grain amyloplast at 10 DAA and 15 DAA. (A10, A15), Function classification of cultivar-specific expressed proteins identified in wheat cultivar ZM366 at 10 DAA and 15 DAA, respectively. (B10, B15), Function classification of cultivar-specific expressed proteins identified in wheat cultivar YM49–198 at 10 DAA and 15 DAA, respectively

Cultivar-specific expressed proteins were identified in grain amyloplasts at 10 DAA and 15 DAA (Additional file 3: Table S3; Additional file 4: Table S4). ZM366 had more specifically expressed proteins than did YM49–198 (Fig. 4a). There were 122 and 81 specifically expressed proteins in ZM366 grain amyloplasts at 10 DAA and 15 DAA, respectively. Correspondingly, there were 19 and 22 specifically expressed proteins in YM49–198 at 10 DAA and 15 DAA, respectively. Based on the functional classification of these specifically expressed proteins (Fig. 5, Additional file 3: Table S3), wheat cultivar ZM366 cultivar-specific proteins were mainly found to be involved in nucleic acid-related (18.03%), stress/defense (13.11%) and carbohydrate metabolism (11.48%) at 10 DAA, whereas the specifically expressed proteins from YM49–198 were mainly involved in carbohydrate metabolism (47.37%), including two starch synthases (A0A1C8E331 and Q6L798) and four granule-bound starch synthases (O81591, Q9AWE1, Q9SBD2 and Q9SBD2), and energetics-related proteins (15.79%). No specifically expressed proteins involved in transport, signal transduction, and protein synthesis/assembly/degradation were found in YM49–198 at 10 DAA.

A similar trend was also seen in the grain amyloplast at 15 DAA (Fig. 5, A15, B15; Additional file 4: Table S4). Cultivar-specific expressed proteins were mainly involved in carbohydrate metabolism in ZM366 and YM49–198 (14.81% and 22.73%, respectively), and included two starch metabolism proteins in YM49–198 (A0A1D6L3I4 and A9UGN1); stress/defense proteins (9.88% and 22.73%), including four ROS scavenging system proteins (H2DPU3, M7YQT7, W5AQ87, and M8CFS7) in ZM366; and two peroxidases (A0A1D5X0A7 and A0A1D6BF22) in YM49–198, and as well as nucleic acid-related proteins (7.41% and 18.18% in ZM366 and YM49–198, respectively).

### Identification of differentially-expressed proteins in the two wheat cultivars

**Fig. 6:**
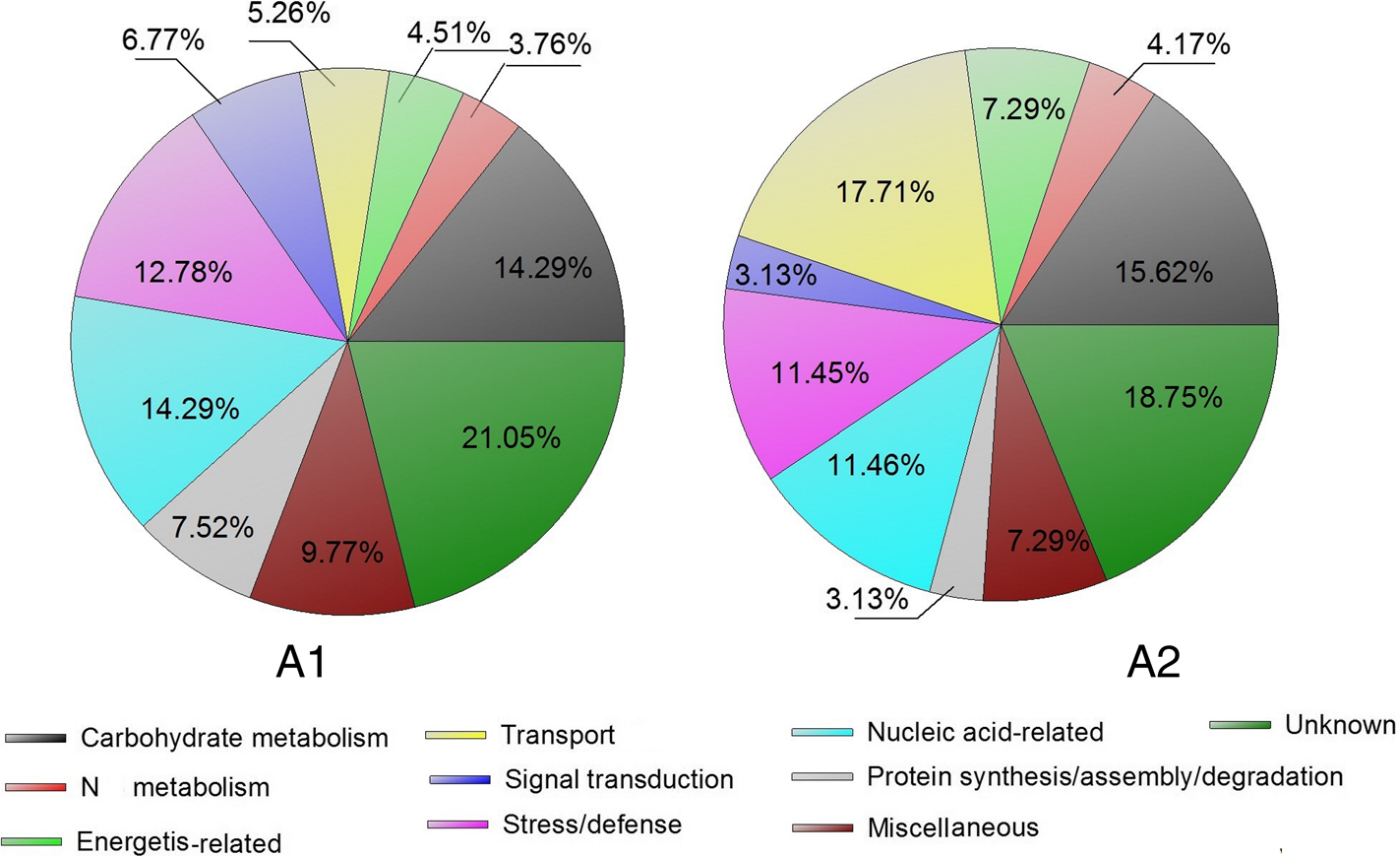
Function classifications of DEPs identified in grain amyloplast between wheat cultivar ZM366 and YM49–198. A1, DEPs identified in wheat grain at 10 DAA; A2, DEPs identified in wheat grain at 15 DAA

The number of differentially expressed proteins in the two wheat cultivars is shown in Fig. 4b. There were 133 DEPs at 10 DAA, with 97 showing up-regulation expression levels in ZM366 and 36 in YM49–198. Similarly, 96 DEPs were found at 15 DAA, including 63 in ZM366 and 33 in YM49–198 that were up-regulated. The DEPs were classified into ten functional categories (Fig. 6). The protein categories were: carbohydrate metabolism (14.29–15.62%), N metabolism (3.76–4.17%), energetics-related (4.51–7.29%), transport (5.26–17.71%), signal transduction (3.13–6.77%), stress/defense (11.45–12.78%), nucleic acid-related (11.46–14.29%), protein synthesis/assembly/degradation (3.13–7.52%), miscellaneous (7.29–9.77%), and unknown (18.75–21.05%).

There were 19 DEPs at 10 DAA involved in carbohydrate metabolism (Additional file 5: Table S5), including one starch synthase (A0A1D5T6Q3) and one beta-amylase (A0A1D5XGF3), and 10 glycolysis process proteins. The expression of alpha-galactoside (W5F620) in ZM366 was 2.16-fold higher than in YM49–198. Similarly, 15 DEPs involved in carbohydrate metabolism were found at 15 DAA, of which 14 were expressed at higher levels in ZM366 and only one DEP (P7W9X7) showed higher expression in YM49–198. There were seven and 17 DEPs involved in transport processes at 10 DAA and 15 DAA, respectively. The DEPs at 15 DAA included five that showed higher expression in YM49–198 and 12 that were up-regulated in ZM366, of which there was one ADP-glucose brittle-1 transporter (A0A1D6AX03) and two transmembrane 9 superfamily members (M8C0V9 and A0A1D5ZTV6). There were nine DEPs at 10 DAA that participate in signal transduction; six were up-regulated in ZM366, including one 14–3-3a protein (P29305) and four GTPases (A0A1D5SVG3, A0A1D5SB20, A0A077S025, and A0A1D6CFI9). At 15 DAA, we found three DPEs involved in signal transduction, of which all were up-regulated in YM49–198. There were 17 DEPs at 10 DAA involved in stress/defense, of which 10 were up-regulated in YM49–198 and seven were up-regulated in ZM366. The up-regulated proteins in YM49–198 included two serine-type endopeptidase inhibitors, five alpha-amylase/trypsin inhibitors, and two peroxidases (Q84U03 and A0A1D6BF22), while the up-regulated proteins in ZM366 were mainly chaperonins and heat shock proteins (S4VQP9, N1R361, A0A1D5YB80, M8AVT1, and F4Y5B2). Similarly, there were 11 DEPs at 15 DAA that participate in stress/defense; of these, six were up-regulated in YM49–198 including four trypsin inhibitors (A0A1D6D8Z1, B5B0D5, P81713, and A0A1D5X8F8), and five were up-regulated in ZM366 including ROS proteins (A3FKE5 and A0A1D6CM14) and a heat-shock protein (M7YWA0). Additionally, the expression of the heat-shock protein M7YWA0 was 20-fold higher in ZM366 than that of in YM49–198. There were 19 nucleic acid-related process DEPs at 10 DAA, of which 16 were up-regulated in ZM366 including three translation initiation factors and four elongation factors. However, 11 nucleic acid-related process DEPs were found at 15 DAA, and of these, six were translation initiation/elongation factors (M8AZD7, A0A1D6B1C3, A3RCW1, A0A1D5TKE5, A0A1E5W0T3, and A0A1D5ZWW7). There were 10 DEPs that participate in protein biosynthesis/assembly/degradation at 10 DAA, whereas only three DEPs were found at 15 DAA. There were five up-regulated DEPs in YM49–198, and eight up-regulated DEPs in ZM366, of which the expression of a ubiquitin-activating enzyme (P20973) in ZM366 was 6.62-fold and 3.45-fold higher than those of in YM49–198 at 10 DAA and 15 DAA, respectively. In total, there were 20 DEPs in the miscellaneous biological process group, of which four were up-regulated in YM49–198 and 16 were up-regulated in ZM366. In addition, there were 46 DEPs of unknown function.

### Comparison of the expression patterns of identified proteins at the mRNA and protein levels

**Fig. 7:**
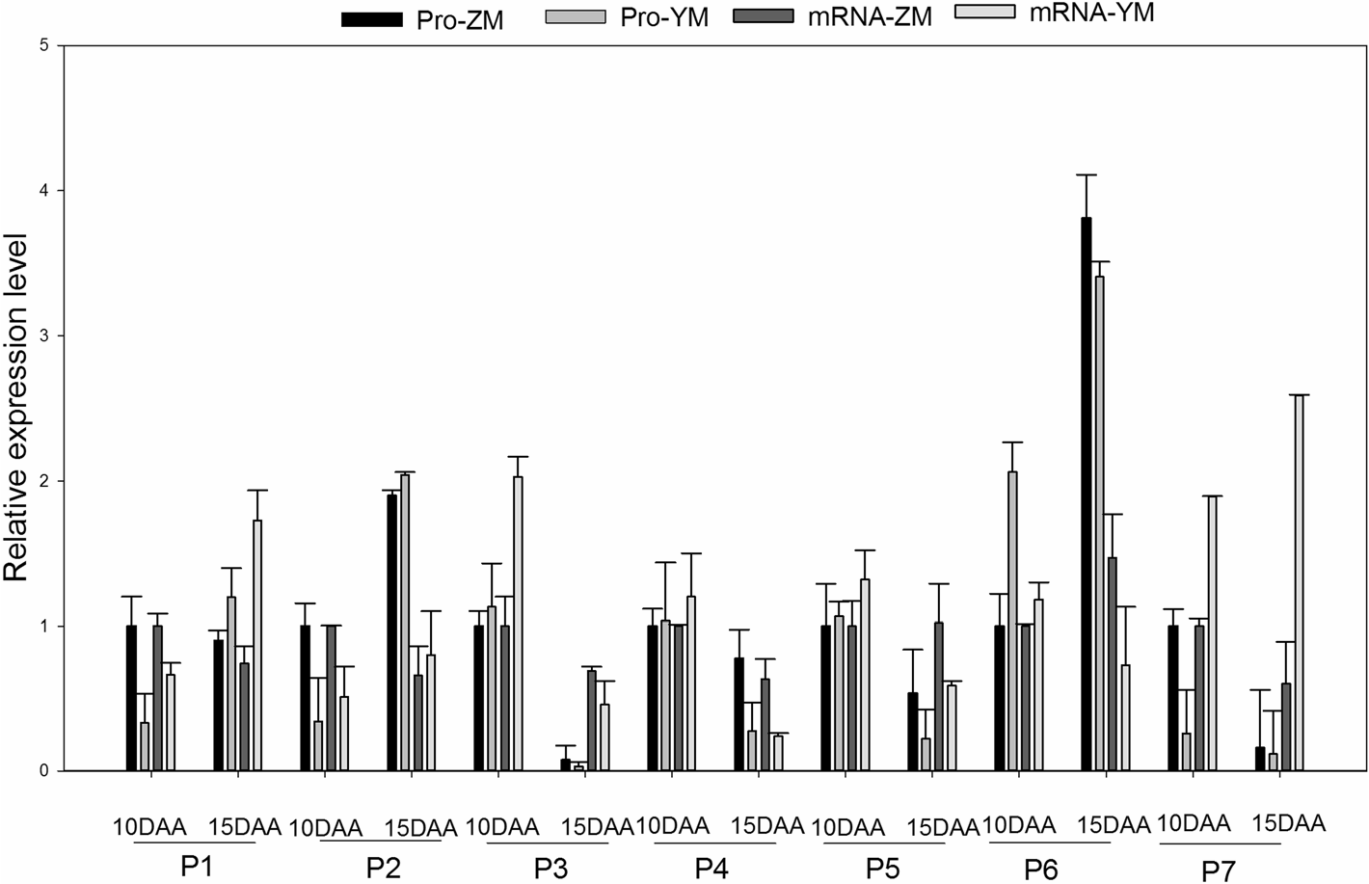
Comparison of protein and mRNA expression patterns of 7 representative DEPs at 10 DAA and 15 DAA. Pro-ZM and Pro-YM represent protein expression pattern of cultivar ZM366 and YM49–198, respectively. mRNA-ZM and mRNA-YM represent mRNA expression pattern of cultivar ZM366 and YM49–198, respectively. P1, P2, P3, P4, P5, P6, P7 represent protein A0A1D6D1Q3, A0A1D5YUK3, P12782, M7ZFX1, A0A1D5VEF1, and M8A7K2, respectively

The relative expression levels of seven representative DEPs and the transcription of the corresponding genes are shown in Fig. 7. The sequences of the gene-specific primers are given in Table 2. Three proteins (A0A1D6D1Q3, A0A1D5YUK3, and P12782) are involved in carbohydrate metabolism. Two transporter proteins (M7ZDS1 and M7ZFX1) were down-regulated from 10 DAA to 15 DAA. One protein (A0A1D5VEF1) belongs to the nucleic acid-related protein group, and one protein (M8A7K2) is involved in protein biosynthesis/assembly/degradation. The mRNA levels for four genes encoding pyrophosphate-fructose 6-phosphate 1-phosphotransferase subunit beta (A0A1D6D1Q3), phosphoglycerate kinase (P12782), 2-oxoglutarate/malate carrier protein (M7ZDS1), and an outer membrane porin (M7ZFX1) were highly consistent with their protein levels, and the relative mRNA and protein levels were very similar for two genes encoding pyrophosphate-fructose 6-phosphate 1-phosphotransferase subunit alpha (A0A1D5YUK3) and zinc finger protein (A0A1D5VEF1). However, the relative expression of one gene that encodes a DnaJ protein-like protein (M8A7K2) showed the opposite pattern with respect to the protein levels in both cultivars at both 10 and 15 DAA.

**Table 2:** Names and sequences of the oligonucleotide primers used for gene amplification

Protein ID in Uniprot	Primer Sequence (5′–3′)		Reference Gene ID in NCBI
A0A1D6D1Q3	F	AGGGCTGATGTATCGGTTCT	AK450587
	R	CTGCGTTGATGGATGTTGAG	
A0A1D5YUK3	F	AGTCACCTGGAGGGCACAA	HX137926.1
	R	CCCACCAATAAATCCGTAGA	
P12782	F	ATTCGGAACAGCCCACAGAG	X15233.1
	R	ATGGGCGCTTAGGGTTTGAA	
M7ZDS1	F	GCGTCTGCTACACTTCCT	AK448820.1
	R	AATGGCTTGATCGTCTTC	
M7ZFX1	F	CCTCGGTTTCGGATGA	X77733.1
	R	CAACGGACAGGCACTACA	
A0A1D5VEF1	F	CCACTATGCAATCTGGGCCA	AK455773
	R	ACCTGCACAATCCAGTCCTG	
M8A7K2	F	CCCGACTCGCTGAACCT	DQ789026.1
	R	CCATCGTCTCCTCGCACT	
Actin	F	AGCGGTCGAACAACTGGTA	AB181991
	R	AAACGAAGGATAGCATGAGGAAGG	

### Protein-protein interaction analysis of the DEPs

Protein-protein interaction (PPI) networks of the DEPs identified in this study were analyzed using IntAct (http://www.ebi.ac.uk/intact/main.xhtml). As shown in Additional file 6: Figure S1, the PPI network of the DEPs identified in ZM366 between 10 DAA and 15 DAA contained 73 nodes and 295 edges. Seventy-three DEPs involved in eight functional categories were used to construct the PPI network. Four stress/defense proteins, two transporters, and 34 carbohydrate metabolism proteins were involved in this network. Similarly, the PPI network of DEPs identified in wheat cultivar YM49–198 at 10 DAA and 15 DAA contained 76 nodes and 237 edges (Additional file 7: Figure S2).

## Discussion

### Grain characteristics of the two wheat cultivars

Wheat endosperm contains two predominant starch granule populations: A-type granules that are larger in size (> 9.9 μm) and are initiated early in the grain filling period, and B-type starch granules that are smaller in size (< 9.9 μm) and are initiated during the later stages of grain filling [8, 29]. It has been reported that A-type starch granules begin forming at 4–5 DAA, and that each amyloplst contains one A-type starch granule [29, 30]. The B-type starch granules are initiated at 12–14 DAA from A-type granules [29–31]. In this study, we found that the average volume distribution and average surface distribution of starch granules > 9.9 μm were all lower at 15 DAA (59.18%, and 15.80%, respectively) than that at 10 DAA (60.26% and 19.63%, respectively), whereas the number distribution of granules > 10 μm was 0.125% at 10 DAA and 0.075% at 15 DAA. These results indicate that there are more A-type starch granules at 10 DAA than at 15 DAA. Hard wheat has more B-type granules (< 9.9 μm) and fewer granules between 22.8 and 42.8 μm than soft wheat [9]. Here, we found that hard wheat cultivar ZM366 had more small starch granules than did the soft wheat cultivar YM49–198; the different starch granule distribution may contribute to the suitability for different food-making qualities [32, 33].

### Proteome analysis of the wheat grain amyloplast

Amyloplasts are specialized organelles responsible for the synthesis and storage of starch. Understanding amyloplast metabolism will provide more useful information about starch biosynthesis and starch physiochemical characteristics. To gain additional insight into the role of amyloplast metabolism, the amyloplast proteins from two different wheat cultivars at two developmental stages were analyzed in this study. We found that multiple metabolic processes, including energy and carbon metabolism, signal transduction, stress/defense, transport, nucleic acid-related, and protein synthesis/assembly/degradation were involved in amyloplast metabolism.

### Carbohydrate metabolism

Carbohydrate metabolism is one of the most important metabolic processes during grain development. A previous report describing the wheat grain proteome found that 21% of the DEPs were involved in carbohydrate metabolism [25]. Here, proteome analysis of the amyloplast at two grain developmental stages showed that an average of 21.50% of the DEPs participate in carbohydrate metabolism, indicating that carbohydrate metabolism is also one of the primary functions of the amyloplast. Amyloplasts are the major site of synthesis and long-term storage of starch in the endosperm [10]. Here, only some of the amyloplast DEPs detected were involved in starch metabolism, which is in agreement with Dupont [22]. Starch biosynthesis in plants involves the concerted action of a number of enzymes, including ADP-glucose pyrophosphorylase (ADPGase), starch synthases (SS), granule-bound starch synthase (GBSS), starch branching enzymes (SBE), and debranching enzymes (DBE) [2, 3, 34–37]. SS and SBE are related to amylopectin synthesis, and amylose synthesis is controlled by GBSS [35]. We found that two SSs (Q43654 and A0A1D5T6Q3), two SBEs (A0A1D6RLR1 and G3CCE7) and a single 1, 4-alpha-glucan branching enzyme (A0A1D5U5L3) were significantly down-regulated in the 15 DAA sample compared to 10 DAA. However, no significant DEPs were found related to GBSS, which indicates that there are differences between amylose and amylopectin biosynthesis during grain development. In addition, the expression levels of SSs at 15 DAA were 0.03~ 0.04-fold higher than those of at 10DAA, whereas that of SBE were 0.23~ 0.32-fold. The different levels of down-regulated expression maybe contribute to different starch physicochemical properties. Toyosawa et al. [38] found that SSIIIa have a crucial role in determining granule morphology and in maintaining the amyloplast envelopment structure in rice seeds; whereas SBE mutation altered the fine structure of amylopectin, and the endosperm starch from the *sbe1* mutant had a lower onset temperature for thermo-gelatinization compared with the wild type [39]. Of course, just as Wang et al. [40], speculated that these enzymes proteins may have a coordinating action in starch biosynthesis within the amyloplast, operating as functional multiprotein complexes. The different expression patterns of these DEPs may partly reflect that there is coordination mechanism of starch synthesis in wheat grains. The SS and SBE in the two wheat cultivars showed the same expression patterns from 10 DAA to 15 DAA. ZM366 had relatively higher expression levels of SS (A0A1D5T6Q3) than did YM49–198 at 10 DAA, and YM49–198 showed cultivar-specific expression of five GBSS proteins (A0A1D6L3I4, O81591, Q9AWE1, Q9SBD2, and D71C0) at 10 and 15 DAA; all of these differences could contribute to the synthesis of different types of starch and result in different starch properties between two wheat cultivars. Liu et al. [41], suggested that the change of GBSS activity were consistent with the amylose content indicating that amylose in grain are determined by GBSS activity, especially at later grain filling stages. Here, we also found that YM49–198 had a higher amylose content than did ZM366 (data not shown), which indicates that these DEPs may contribute to amylose accumulation. The study of SSIIa/SSIIIa double repression lines revealed that the double mutation had increased pasting temperatures, and decreased viscosities, and also affected the fine structure of amylopectin [42]. The up-regulated expression of SS in ZM366 may be related to starch properties. Here, only DEPs in amyloplast at the early grain developmental stages were studied, and the metabolic process of amyloplast at the later grain developmental stages need further study.

Alph- and beta-amylases required for starch degradation were not detected in wheat grain amyloplasts by Dupont [22]. In our study, YM49–198 had relatively higher expression levels of beta-amylases (A0A1D5XGF3) than did ZM366 at 10 DAA, which may indicate that there are different degrees of starch degradation in wheat grain. However, Agrawal [43] found that triticale S71–142 had more amylase activity at certain stages of grain development than triticale IMJ3 but the grain of S71–142 was much better than IMJ3, indicating that amylases are not related to the grain shriveling. Further studies are needed on the role of amylase in starch synthesis during grain development. Interestingly, six and eight DEPs between 10 DAA and 15 DAA that involve the photosystems, including chlorophyll 1-b binding protein, were detected in ZM366 and YM49–198, respectively. It has been hypothesized that these proteins are actual amyloplast constituents, and do not result from contamination with chloroplast proteins [22]. Amyloplasts are non-pigmented plastids and, along with chloroplasts, are derived from proplastids [44]. It is clear that starch synthesis and storage also take place in chloroplasts; amyloplasts and chloroplasts are closely related, and it was found that potato amyloplasts can turn into chloroplasts in the light [45]. Thus, it is reasonable that photosynthesis-related proteins are detected in amyloplasts.

Apart from photosynthesis and starch metabolism, we found that some DEPs involved in other types of carbohydrate metabolic processes were up-regulated from 10 DAA to 15 DAA, but others were down-regulated. The broad distribution of carbohydrate metabolism enzymes suggests that plastids are actively involved in other processes in addition to starch synthesis [46]. Analysis of the rice ADP-glucose (ADPG) transporter also showed that there are one or more biochemical processes in amyloplast stroma that control carbon flux into starch, which affects starch synthesis and kernel weight [15]. Additionally, even though the two wheat cultivars in our study showed similar protein expression patterns with grain development, cultivar ZM366 had more up-regulated DEPs and cultivar-specific proteins than did YM49–198. These results indicate that the amyloplasts in the two wheat cultivars have similar functions, and that the DEPs between the two wheat cultivars and the cultivar-specific proteins may be responsible for the differences in both starch content and characteristics. Further research is needed to define the regulatory role of amyloplast on starch synthesis.

### Transport proteins

Membrane transporters serve as internal and external exchange components in the amyloplast [47]. It has been reported that the majority of ADPG in cereal grain endosperm is generated in cytosol from AGPase [48] and is subsequently transported into amyloplasts by the BRITTLE-1 (BT1) protein located in the plastid envelope [49, 50]. A study of the rice BRITTLE1 mutation demonstrated that ADPG transport by BT1 is essential for the normal rate of starch synthesis in rice endosperm [15]. Here, we found that two brittle-1 transporter proteins (A0A1D6AX03 and A0A1D6DJD7) in the two wheat cultivars were up-regulated at 15 DAA compared with 10 DAA, indicating that more ADPG transport occurs with grain development. And for BRITTLE-1 protein transport of ADPG, there are different exchange substrates. Biochemical transport studies of maize BT1 showed that it imports ADPG through counter exchange with ADP [51]. Recombinant BT1 protein synthesized from potato (*Solanum tuberosum*) showed that StBT1 does not transport ADPG but does transport AMP, ADP, and ATP, and that the transport of AMP, ADP, and ATP occurs in a unidirectional rather than antiportal mode [52]. AtBT1 is a plastidal nucleotide uniport carrier protein that is strictly required for the export of newly synthesized adenylates into the cytosol [53]. The wheat BT1 homolog also transports ADPG but has similar affinities for ADP and AMP as the counter-exchange substrates [54]. In this study, we do not know the identity of the exchange substrate of the BRITTLE-1 protein, but two ADP, ATP carrier proteins were significantly down-regulated at 15 DAA. The expression pattern of the BRITTLE-1 protein suggests a difference in amyloplast metabolism at the different developmental stages; the mode of specific transport of ADPG may also need further investigation. Also, we found that ZM366 had higher levels of BRITTLE-1 transporter expressed at 15 DAA compared to YM49–198. However, the starch content is slightly lower in ZM366, which could indicate that the regulation of other metabolic processes on starch synthesis affects starch content. Overexpression of the maize protein ZmBt1 in rice lines showing elevated ADPG levels in the amyloplasts did not lead to further increases in seed weight [15]. Additionally, some essential DEPs for ion channels, transmembrane family proteins, and translocators were detected with grain development and between the two wheat cultivars, suggesting that there is complexity in the transport machinery of the plastid envelope, and that transport varies with grain development and between wheat cultivars.

### Stress/defense

A number of stress/defense proteins that act against biotic or abiotic stress are expressed throughout wheat grain development. Serpin family proteins play an important role in plant growth, development, and the stress response by irreversible inhibition of endogenous and exogenous proteinases [55]. It has been suggested that wheat grain serpins probably protect storage proteins from digestion [24, 56]. In our study, the expression levels of five serpins (C0LF31, C0LF30, A0A1D5ZBL7, H9AXB3, and Q9ST57) in the two wheat cultivars were increased at 15 DAA, especially in wheat cultivar ZM366, and the average expression level increased by almost 52-fold. Two serpins, H9AXB3 and Q9ST57, were reported to be phosphorylated [24], and phosphorylation would increase their activity and protect the proteins from degradation. α-Amylase inhibitors also play important roles in protecting starch and proteins in the endosperm from degradation [57]. Here, α-amylase inhibitors (five in ZM366 and six in YM49–198) were found to accumulate to high levels in the amyloplasts from 10 to 15 DAA. α-amylase inhibitor DEPs have also been found in grain from 21 to 42 DAA [25]. Apart from serpins, small heat shock proteins and chaperone proteins are involved in a wide range of cellular functions. For example, Hsp70 can stabilize protein conformation, prevent aggregation, and maintain non-native proteins in a competent state [58]. Unlike the expression patterns of serpins and α-amylase inhibitors, heat shock and chaperone proteins all showed higher expression levels at 10 DAA compared to 15 DAA. These different expression patterns may be ascribed to different stress responses at different stages of development.

Plant exposed to abiotic and biotic stresses can induce the elevated production of ROS and break down the balance between ROS production and antioxidant defenses [59]. Plants have evolved complex enzymatic and non-enzymatic antioxidant defense systems to mitigate cellular oxidative damage [60–62]. In the present study, nine DEPs (three in YM49–198 and six in ZM366) are involved in ROS scavenging systems, and include three that were up-regulated (peroxidase, pyrroline-5-carboxylate reductase, and betaine-aldehyde dehydrogenase) and three that were down-regulated (L-ascorbate peroxidase 2, peroxiredoxin, and 12-oxophytodienoate reductase 2) from 10 DAA to 15 DAA. The different expression patterns of stress/defense related proteins may reflect their different functions in the response to diverse stress conditions at different developmental stages. Additionally, there were some proteins that showed different expression patterns between the two wheat cultivars at the same stage; these proteins that showed response differences could play roles in yield-related traits and quality-related traits. This is similar to previous reports that proteins abundances differing between two cultivars were possibly associated with yield-related traits in bread wheat [25, 63].

### Nucleic acid-related proteins

The DEPs involved in nucleic acid-related processes were 14.25% of the total DEPs detected in our study, which was similar to a previous finding in wheat amyloplasts at 10 DAA [15]. Here, the DEPs were mainly RNA/DNA binding proteins, ribosomal proteins, and translation/elongation factors. Interestingly, most of the RNA/DNA binding and ribosomal proteins were up-regulated, while the translation initiation/elongation factors were down-regulated from 10 DAA to 15 DAA. In the two wheat cultivars, ZM366 had more highly expressed proteins at 10 DAA than did YM49–198, while the numbers of highly expressed proteins were comparable at 15 DAA. A study of amyloplast DNA showed that there was a large increase in the amount of plastid DNA (ptDNA) per endosperm between 9 and ~ 15 DAA, and the average number of ptDNA copies per amyloplast increased from 10 copies at 9 DAA to ~ 50 copies in the mature amyloplast [12]. The differential expression of nucleic acid-related proteins may be related to the different number of ptDNA copies present in the amyloplast. It has even been suggested that most proteins required by amyloplasts are encoded in the nucleus [64, 65], and the possibility remains that some functions related to starch accumulation in amyloplasts require proteins encoded by the plastid genome [12].

### Other functional proteins

14–3-3 proteins can regulate cell growth by interaction with Raf-1 in the Raf-1/ERK pathway [66]. In addition, these proteins can regulate cell growth and survival by promoting big mitogen-activated protein kinase (BMK1) [67]. The expression of genes for 14–3-3b and 14–3-3c showed a decreasing tendency with advancing grain development [68]. Here, we found that 14–3-3 proteins in the two wheat cultivars were up-regulated from 10 DAA to 15 DAA. Cultivar differences as well as post-transcriptional and translational regulation and protein degradation could explain this discrepancy [69]. It has been reported that there are hundreds of closely related forms [70], and at least one family member, a 14–3-3 protein from the ε-group, may directly regulate the synthesis of starch by binding to SSIII [71]. Research in cassava has also revealed that 14–3-3 proteins and their binding enzymes may play important roles in carbohydrate metabolism and starch accumulation during root tuberization [72]. In our study, expression of one 14–3-3 protein (A0A1D5WBE0) was 3.55-fold higher in YM49–198 than in ZM366, while the level of another 14–3-3a protein (P29305) was 3.14-fold higher in ZM366 than in YM49–198 at 10 DAA. These differences possibly indicate that the 14–3-3 proteins that differ between the two cultivars may be related to variations in the starch content and its characteristics in wheat cultivars ZM366 and YM49–198. The relationships between 14-3-3 proteins and starch accumulation, as well as the pattern of regulation, need to be investigated in future studies.

Plant aspartic proteinases (APs) are mainly involved in the processing of precursor proteins, protein degradation, plant disease resistance, and abiotic stress tolerance [73]. The relative expression of rice aspartic proteinase gene *oryzasin* increased from 2 to 4 weeks after anthesis, indicating that *oryzasin* plays an important role during rice seed development [74]. We found that three aspartic proteinase proteins (A0A1D6ABF1, A0A1D5VCN0, and M8C6C1) were up-regulated at 15 DAA, especially for A0A1D5VCN0, which was 8.88-fold higher at 15 DAA than at 10 DAA at ZM366. Thus, these aspartic proteinases could possibly play a regulatory role in amyloplast during grain development.

Amyloplasts may not only function as sites for starch synthesis and storage, they could also play important roles in regulating metabolism. The diversity of amyloplast metabolic processes may be related to the origin of amyloplast. Badenhuizen [75] reported that A-type amyloplasts are derived from plastids, while B-type amyloplasts come from mitochondria. Buttrose [76] suggested that small granules form in vesicles budded off from outgrowths of the A-type granule-containing amyloplasts. Other findings suggest that amyloplasts in wheat endosperm divide and increase in number through protrusions [30, 77, 78]. The regulatory role of amyloplasts in grain development and the function of proteins specifically expressed in wheat amyloplast need further study.

## Conclusions

Label-free-based proteome analysis showed that apart from starch metabolism, wheat grain amyloplasts have broad metabolic capabilities, such as carbohydrate metabolism, stress/defense, transport, signal transduction, and nucleic acid-related processes. The similar expression patterns of the DEPs in the two wheat cultivars between 10 DAA and 15 DAA reveal that the complex regulation processes might be related to amyloplast development and starch accumulation. Proteins that are expressed differently between the two wheat cultivars may contribute to differences in kernel weights and starch characteristics. Our results have provided specific proteomic insights into wheat amyloplast metabolism at two stages of grain development, and will help to increase our understanding of the role of the amyloplast in grain development.

## Materials and methods

### Experimental design

Two winter wheat (*Triticum aestivum* L.) cultivars, hard wheat cv. ‘Zhengmai366’ (ZM366), and soft wheat cv. ‘Yumai49–198’ (YM49–198) were used in this study. The grain characteristics of the two wheat cultivars are given in Table 1. Wheat seeds were sown during the 2016–2017 growing season at Henan Agricultural University Experimental Station, Zhengzhou, Henan province, China (34°44′ N, 113°42′ E). The soil is loamy Fluvoaquic with an organic matter content of 16.47 mg kg^− 1^ (0–30 cm), available phosphorus of 20.83 mg kg^− 1^, available potassium of 212.56 mg kg^− 1^, and a pH of 7.91. Seeds were sown on 14 October 2016. The plot dimensions were 3 m × 7 m and the sowing density was 160 seeds/m^2^. Field trials were managed based on local agronomic practices.

### Sampling

At flowering, spikes undergoing anthesis on the same day were tagged. For amyloplast preparation, the tagged spikelets were harvested separately at 10 and 15 days after anthesis (DAA). Three replicates of each grain sample were used as biological replicates for each stage for the two wheat cultivars. The harvest spikes were used immediately for amyloplast preparation. In addition, more tagged spikes harvested at the same times and stored for biochemical analyses.

### Amyloplast preparation

Wheat amyloplasts were isolated using the methods of Andon et al. [21] and Balmer et al. [19]. Thirty heads were harvested and used within a 2 h period for each preparation. Embryos were removed from the grains, and the wheat endosperm was gently squeezed from the whole caryopsis and collected in ice-cold buffer (0.5 M sorbitol, 50 mM HEPES pH 7.5). The collected endosperm was transferred to plasmolysis buffer (0.8 M sorbitol, 50 mM HEPES pH 7.5, 1 mM EDTA, 1 mM KCl, 2 mM MgCl_2_) and allowed to plasmolyse for 1 h at 4 °C. The plasmolysed endosperm tissue was then chopped with a razor blade in chopping buffer (0.8 M sorbitol, 50 mM HEPES pH 7.5, 1 mM EDTA, 1 mM KCl, 2 mM MgCl_2_, 0.1% *w*/*v* BSA). The resulting homogenate was filtered through two layers of Miracloth (Millipore Sigman) onto a 6 ml Nycodenz cushion (Nycomed, Oslo, Norway) and dissolved in chopping buffer. Amyloplasts were separated from the endosperm extract by centrifugation at 30×g for 10 min at 4 °C. The pellet containing the amyloplasts was gently suspended in chopping buffer and the Nycodenz procedure was repeated once more.

### Protein extraction

The purified amyloplast samples were mixed with 100 volumes of SDT buffer (4% SDS, 100 mM DTT, 150 mM Tris-HCl pH 8.0) sonicated (80 W, working 10 s, interval 15 s; repeated 10 times), then boiled for 5 min. After centrifugation at 14,000×g for 40 min, the supernatants were retained. The protein concentrations were quantified with the BCA Protein Assay Kit (Bio-Rad, USA), and the samples were stored at − 80 °C.

### FASP digestion

For each sample, 30 μL of extracted proteins were dissolved in DTT buffer and boiled for 5 min. After cooling to room temperature, 200 μL of UA buffer was added. The solution was transferred to an ultrafiltration centrifuge tube (Microcon units, 10kD) and centrifuged at 14,000×g for 15 min. The filter was discarded. The process was repeated one time. Iodoacetamide (100 μl of 100 mM IAA in UA buffer) was then added, and the mixture was incubated for 30 min in darkness. The filters were washed with 100 μl UA buffer three times, and then washed with 100 μl of 25 mM NH_4_HCO_3_. After incubation at 37 °C for 16–18 h, the samples were centrifuged at 14,000×g for 15 min and the filters were kept. This process was repeated twice. The peptides from each sample were desalted on C_18_ Cartridges (Empore™ SPE Cartridges C18 (standard density); bed I.D. 7 mm, volume 3 ml; Sigma Aldrich), concentrated by vacuum centrifugation, and reconstituted in 40 μl of 0.1% (*v*/v) formic acid.

### MS/MS protein identification and quantification

Each fraction was injected for nanoLC-MS/MS analysis. The peptide mixture was loaded onto a reverse-phase trap column (Thermo Scientific Acclaim PepMap100, 100 μm × 2 cm, nanoViper C18) connected to the C18-reverse phase analytical column (Thermo Scientific Easy Column, 10 cm long, 75 μm inner diameter, 3 μm, C18-A2) in buffer A (0.1% formic acid) and separated with a linear gradient of buffer B (84% acetonitrile, 0.1% formic acid) at a flow rate of 300 nl/min controlled by IntelliFlow technology.

LC-MS/MS analysis was performed on a Q Exactive mass spectrometer (Thermo Scientific) coupled to an Easy nLC (Proxeon Biosystems, now ThermoFisher Scientific) for 240 min. The mass spectrometer was operated in positive ion mode. MS data was acquired using a data-dependent top10 method, dynamically choosing the most abundant precursor ions from the survey scan (300–1800 m/z) for higher collision energy dissociation (HCD) fragmentation. The automatic gain control (AGC) target was set to 1e6, and the maximum injection time to 50 ms. The dynamic exclusion duration was 60.0 s. Survey scans were acquired at a resolution of 70,000 at 200 m/z and resolution for HCD spectra was set to 17,500 at m/z 200, the isolation width was 2 m/z, normalized collision energy was 30 eV, and the under-fill ratio was defined as 0.1%.

For protein identification, the MS raw files were processed by MaxQuant v.1.3.0.5 software [79]. The acquired MS/MS spectra were automatically searched against the uniprot_pooideae_1440380.fasta (Jan 04, 2017), and the total number of protein sequences used in this database was 1,440,380. The minimum peptide length was set to six amino acids and the maximum false discovery rate (FDR) to 1% for both peptides and proteins. The other parameters were set as: peptide mass tolerance = ± 20 ppm; enzyme = trypsin; max missed cleavage = 2; fixed modification: carbamidomethyl (C); variable modification: oxidation (M), Acetyl (Protein N-term). Protein quantification was based on both ‘razor’ and unique peptides [79, 80], and the Label free quantitation (LFQ) algorithm was performed [81]. For each fraction, peptides were matched across different LC-MS/MS runs based on mass and retention time (‘match between runs’ option in MaxQuant) using the time window of 2 min.

Differentially accumulated proteins were analyzed for significant downregulation or upregulation. For quantitative changes, a 2.0-fold cutoff was set to determine up-accumulated and down-accumulated proteins, with a *p*-value < 0.05 present in at least two replicates.

### Bioinformatics analysis

Functional annotation analysis was performed with Blast2GO software (http://www.geneontology.org/) [82]. We used the KAAS (Automatic Annotation Server) software to annotate differentially expressed proteins and to investigate the biochemical pathways of molecular interactions [83].

### RNA extraction, primer design, and real-time PCR

Total RNA was extracted from grain using TriZol Reagent (Invitrogen, Carlsbad, CA, USA) according to the manufacturer’s instructions. Seven differentially accumulated proteins were selected to investigate their expression levels at the mRNA level. Gene expression analysis was performed using SYBR Premix ExTaq (Promega Biotechnology [Beijing] Co., Ltd.), and the experiments were performed according to the manufacturer’s instructions. All qPCR experiments included two biological replicates and three technical replicates. Primer pairs for qRT-PCR analysis were designed using Primer 5 software (http:www.premierbiosoft.com) based on the corresponding genomic sequences of the targeted proteins. Primer pairs for A0A1D6D1Q3, A0A1D5YUK3, P12782, M7ZDS1, M7ZFX1, A0A1D5VEF1, and M8A7K2 were designed from the wheat genomic sequences (GeneBank accession: AK450587, HX137926.1, X15233.1, AK448820.1, X77733.1, AK455773, and DQ789026.1, respectively), (http://www.ncbi.nlm.nih.gov) (Table 2). For real-time PCR, β-actin was used as the internal reference gene to normalize the relative expression levels of candidate genes in the RNA samples.

### Starch isolation, purification and particle size analysis

Starch was extracted from wheat grains as described by Peng et al. [8] with some modifications. Wheat kernels (2 g) were steeped in 30 mL double distilled water at 4 °C for 24 h. The grains were then de-germed and ground with a mortar and pestle in double distilled water until essentially all the starch granules were released. The slurry was filtered through a 74 μm screen and centrifuged at 1700×g for 10 min to obtain a crude starch pellet. The crude starch was purified three times using 5 mL of 2 M NaCl, 0.2% NaOH, 2% SDS, and double-distilled water. The starch was washed once with acetone to remove the water, then air-dried at room temperature and stored at − 20 °C. Starch particle sizes were determined using a model LS13320 laser diffraction particle size analyzer (Beckman Coulter, USA).

## Additional files

Additional file 1: **Table S1.** Differentially expressed proteins identified in wheat grain amyloplasts between 10 DAA and 15 DAA in the hard wheat cultivar ZM366. (DOCX 55 kb)

Additional file 2: **Table S2.** Differentially expressed proteins identified in wheat grain amyloplasts between 10 DAA and 15 DAA in the soft wheat cultivar YM49–198. (DOCX 54 kb)

Additional file 3: **Table S3.** Cultivar-specific expressed proteins identified in wheat grain amyloplasts of cultivars ZM366 (I) and YM49–198 (II) at 10 DAA. (DOCX 42 kb)

Additional file 4: **Table S4.** Cultivar-specific expressed proteins identified in wheat grain amyloplasts of cultivars ZM366 (I) and YM49–198 (II) at 15 DAA. (DOCX 33 kb)

Additional file 5: **Table S5.** Differentially expressed proteins identified between ZM366 and YM49–198 at 10 DAA (I) and 15 DAA (II). (DOCX 50 kb)

Additional file 6: **Figure S1.** Protein-protein interaction (PPI) network for the DEPs identified in wheat cultivar ZM366 between 10 DAA and 15 DAA. (TIF 4329 kb)

Additional file 7: **Figure S2.** PPI network for the DEPs identified in wheat cultivar YM49–198 between 10 DAA and 15 DAA. (TIF 3495 kb)

## Abbreviations

ADP: Adenosine diphosphate
ADPG: ADP-glucose
ADPGase: ADP-glucose pyrophosphorylase
AMP: Adenosine monophosphate
ATP: Adenosine triphosphate
CAT: Catalase
DAA: Days after anthesis
DBE: Debranching enzyme
DEPs: Differentially expressed proteins
GBSS: Granule-bound starch synthase
POD: Peroxidase
PPI: Protein-protein interaction
PtDNA: Plastid DNA
ROS: Reactive oxygen species
SBS: Starch branching synthase
SDS-PAGE: SDS polyacrylamide gels
SS: Starch synthase
