# Supplementary material for: Quantitative analysis of the grain amyloplast proteome reveals differences in metabolism between two wheat cultivars at two stages of grain development
Source: BMC Genomics. 2018 Oct 24;19:768. doi: 10.1186/s12864-018-5174-z (PMC6201562; doi:10.1186/s12864-018-5174-z)
Supplement: Supplementary file 1 — Table S1. Differentially expressed proteins identified in wheat grain amyloplasts between 10 DAA and 15 DAA in the hard wheat cultivar ZM366. (DOCX 55 kb) [file 12864_2018_5174_MOESM1_ESM.docx]

**Table S1. Differentially expressed proteins identified in wheat grain amyloplasts at 10 DAA and 15 DAA in the hard wheat cultivar ZM366.**

| **Accession No.** | **Species** | **Mr** | **NP** | **Ratio** | **Description** |
| --- | --- | --- | --- | --- | --- |
| **Carbohydrate metabolism** | |  |  |  |  |
| A0A1D5V0H4 | Triticum aestivum | 64.00 | 7 | 2.73 | 4-alpha-glucanotransferase |
| A0A0U4FQJ0 | Triticum aestivum | 57.77 | 23 | 2.78 | Glucose-1-phosphate adenylyltransferase |
| N1R4I4 | Aegilops tauschii | 94.03 | 36 | 7.52 | Sucrose synthase |
| Q8W1W2 | Bambusa oldhamii | 92.12 | 24 | 7.64 | Sucrose synthase |
| M8A0T7 | Triticum monococcum | 92.67 | 36 | 2.17 | Sucrose synthase |
| W5EKI0 | Triticum aestivum | 61.11 | 23 | 18.98 | Beta-amylase |
| A0A1D5XGF3 | Triticum aestivum | 58.73 | 23 | 14.44 | Beta-amylase |
| Q43654 | Triticum aestivum | 71.00 | 14 | 0.03 | Starch synthase 1 |
| A0A1D6RLR1 | Triticum aestivum | 93.08 | 25 | 0.23 | Starch branching enzyme IIa |
| G3CCE7 | Triticum aestivum | 93.01 | 25 | 0.32 | Starch branching enzyme IIa |
| A0A1D5U5L3 | Triticum aestivum | 88.06 | 22 | 0.07 | 1,4-alpha-glucan branching enzyme |
| A0A075BAM5 | Triticum aestivum | 56.18 | 13 | 0.37 | 9POAL Photosystem II CP47 reaction center protein |
| W5BBT6 | Triticum aestivum | 35.19 | 5 | 0.17 | Chlorophyll a-b binding protein, chloroplastic |
| E9KIQ4 | Triticum aestivum | 11.81 | 5 | 0.44 | Cytochrome b559 subunit alpha |
| M0VCJ1 | Triticum aestivum | 34.90 | 7 | 0.27 | Chlorophyll a-b binding protein |
| M7ZUX7 | Triticum aestivum | 23.95 | 6 | 0.42 | Cytochrome b-c1 complex subunit Rieske, |
| U5TXC9 | Triticum aestivum | 26.18 | 5 | 0.22 | Cytochrome b6 |
| W5H2V3 | Triticum aestivum | 63.66 | 18 | 0.14 | RuBisCO large subunit-binding protein subunit beta |
| M8ALL6 | Triticum aestivum | 99.15 | 26 | 0.05 | RuBisCO large subunit-binding protein subunit beta |
| A0A1D5RSC7 | Triticum aestivum | 44.77 | 5 | 2.51 | Alpha-galactosidase |
| A9U8F9 | Triticum aestivum | 41.10 | 11 | 3.55 | Alcohol dehydrogenase ADH3D |
| A0A1D6CIT1 | Triticum aestivum | 41.36 | 8 | 8.86 | Aldose 1-epimerase |
| A0A1D5ZUW3 | Triticum aestivum | 101.2 | 19 | 4.11 | Alpha, alpha-trehalose-phosphate synthase） |
| M7ZAX1 | Triticum aestivum | 38.91 | 11 | 3.62 | Fructose-bisphosphate aldolase |
| A0A1B1V4Q3 | Triticum aestivum | 36.55 | 16 | 4.24 | Glyceraldehyde-3-phosphate dehydrogenase |
| A0A1D5ZEF6 | Triticum aestivum | 72.58 | 17 | 10.41 | Pyrophosphate--fructose 6-phosphate 1-phosphotransferase subunit alpha |
| A0A1D5WP73 | Triticum aestivum | 54.44 | 10 | 54.36 | Pectin acetylesterase |
| M8C5R3 | Triticum aestivum | 41.52 | 4 | 0.32 | Aldose 1-epimerase |
| N1QW20 | Triticum aestivum | 86.31 | 6 | 0.49 | Beta glucosidase |
| A0A1D5YP67 | Triticum aestivum | 53.13 | 16 | 0.02 | Dihydrolipoamide acetyltransferase component of pyruvate dehydrogenase complex |
| P12782 | Triticum aestivum | 49.84 | 21 | 0.07 | Phosphoglycerate kinase |
| M7Z4Y9 | Triticum aestivum | 42.00 | 14 | 0.17 | Fructose-bisphosphate aldolase |
| A0A1D5VT73 | Triticum aestivum | 45.11 | 12 | 0.06 | Fructose-bisphosphate aldolase |
| A0A1D5YMU3 | Triticum aestivum | 40.27 | 7 | 0.43 | Pyruvate dehydrogenase E1 component subunit beta |
| A0A1D6D1N7 | Triticum aestivum | 69.00 | 13 | 0.31 | 6(G)-fructosyltransferase |
| W5FY62 | Triticum aestivum | 67.06 | 16 | 0.04 | Glucose-6-phosphate isomerase |
| A0A1D5X0P4 | Triticum aestivum | 83.65 | 9 | 0.22 | Hydrolyzing O-glycosyl |
| A0A1D6C1W7 | Triticum aestivum | 102.6 | 10 | 0.08 | Hydrolyzing O-glycosyl |
| A0A1D5ZRE6 | Triticum aestivum | 61.76 | 19 | 0.03 | Hydrolyzing O-glycosyl |
| A0A1D5XJY0 | Triticum aestivum | 46.93 | 13 | 0.04 | Glyceraldehyde-3-phosphate dehydrogenase |
| A0A1D5TM13 | Triticum aestivum | 45.89 | 9 | 0.22 | Pyruvate dehydrogenase E1 component subunit alpha |
| R7W4N8 | Triticum aestivum | 46.97 | 6 | 0.43 | Putative acetyl-CoA acetyltransferase |
| A0A1D5WKF4 | Triticum aestivum | 63.65 | 17 | 0.39 | Phosphoglycerate mutase |
| A0A1D5X6Q1 | Triticum aestivum | 54.09 | 7 | 0.05 | 3-phosphoshikimate 1-carboxyvinyltransferase] |
| A0A1D5YNX6 | Triticum aestivum | 68.32 | 9 | 0.48 | Succinate dehydrogenase [ubiquinone] flavoprotein subunit |
| B2ZGK8 | Triticum aestivum | 254.8 | 40 | 0.07 | Acetyl-CoA carboxylase |
| A0A1D6B2M0 | Triticum aestivum | 45.18 | 11 | 0.26 | Succinate-CoA ligase subunit beta |
| M7ZUG3 | Triticum aestivum | 59.61 | 11 | 0.40 | Aldehyde dehydrogenase family 2 member B7 |
| A0A1D5WP39 | Triticum aestivum | 51.82 | 9 | 0.04 | D-glyceraldehyde-3-phosphate glyceronetransferase |
| A0A1D6A7Z4 | Triticum aestivum | 39.92 | 6 | 0.45 | Isocitrate dehydrogenase (NAD) regulatory subunit 1 |
| D2KZ12 | Triticum aestivum | 47.93 | 12 | 0.25 | 3-Ketoacyl-CoA thiolase-like protein |
| W5A4W9 | Triticum aestivum | 41.73 | 10 | 0.13 | L-malate dehydrogenase |
| M7ZLJ5 | Triticum aestivum | 54.68 | 13 | 0.25 | NADH dehydrogenase [ubiquinone] flavoprotein 1 |
| Q35322 | Triticum aestivum | 22.99 | 6 | 0.36 | NADH dehydrogenase, subunit 9 |
| A0A0K0YH82 | Triticum aestivum | 44.45 | 7 | 0.40 | NADH dehydrogenase subunit 7 |
| M8A948 | Triticum aestivum | 71.89 | 8 | 0.50 | 2-oxoglutarate dehydrogenase complex 1 |
| A0A1D5XMV6 | Triticum aestivum | 19.52 | 5 | 0.27 | Putative NADH dehydrogenase (Ubiquinone) 1 alpha subcomplex subunit |
| A0A1D5UWN7 | Triticum aestivum | 58.68 | 12 | 0.30 | Phospho-2-dehydro-3-deoxyheptonate aldolase |
| A0A1D6D7L9 | Triticum aestivum | 87.17 | 5 | 0.44 | Dolichyl-diphosphooligosaccharide--protein glycosyltransferase subunit STT3 |
| A0A1D5U063 | Triticum aestivum | 64.01 | 16 | 0.07 | D-3-phosphoglycerate dehydrogenase |
| A0A1D6RJE7 | Triticum aestivum | 52.52 | 5 | 0.41 | Dolichyl-diphosphooligosaccharide--protein glycosyltransferase subunit 1 |
| M8C5C6 | Triticum aestivum | 58.39 | 15 | 0.28 | Dihydrolipoyl dehydrogenase |
| W5AN92 | Triticum aestivum | 62.13 | 8 | 0.06 | Dihydrolipoyl dehydrogenase |
| **N Metabolism** |  |  |  |  |  |
| A0A077S3V2 | Triticum aestivum | 49.52 | 17 | 2.47 | Aspartate aminotransferase |
| A0A1D5TAT0 | Triticum aestivum | 25.69 | 2 | 7.15 | Aminoacyl-tRNA hydrolase activity |
| M7ZWM8 | Aegilops tauschii | 39.07 | 9 | 2.07 | Serine/threonine phosphatase |
| W5AC96 | Triticum aestivum | 52.14 | 11 | 50.79 | Carboxypeptidase |
| A0A096UK97 | Triticum aestivum | 54.34 | 15 | 0.38 | Aspartic-type endopeptidase |
| W4ZT78 | Triticum aestivum | 52.13 | 12 | 0.14 | Argininosuccinate synthase |
| M7Z423 | Triticum aestivum | 125.7 | 26 | 0.02 | Carbamoyl-phosphate synthase large chain |
| A0A1D6CWT9 | Triticum aestivum | 49.65 | 13 | 0.03 | Glutamate-1-semialdehyde 2,1-aminomutase |
| A0A1D5RP81 | Triticum aestivum | 67.41 | 13 | 0.15 | Ketol-acid reductoisomerase |
| A0A1D6A1U6 | Triticum aestivum | 45.12 | 6 | 0.42 | Sulfurtransferase |
| S5YTU4 | Triticum aestivum | 235.1 | 20 | 0.17 | NADH-dependent glutamate synthase |
| A0A1D6S7W1 | Triticum aestivum | 56.08 | 7 | 0.30 | Acetolactate synthase |
| **Energetics-related** | |  |  |  |  |
| B3VEX2 | Triticum aestivum | 29.62 | 2 | 2.35 | L Cytochrome c oxidase subunit 2 |
| A8Y9G7 | Triticum aestivum | 55.74 | 20 | 0.04 | ATP synthase subunit alpha |
| A0A1D5T2S6 | Triticum aestivum | 35.44 | 10 | 0.42 | Proton-transporting ATP synthase |
| A0A1D6SDU1 | Triticum aestivum | 74.32 | 22 | 0.35 | Proton-transporting ATPase |
| M8AIK4 | Triticum aestivum | 104.9 | 25 | 0.24 | Plasma membrane ATPase |
| A0A1D5RP45 | Triticum aestivum | 63.53 | 20 | 0.46 | ATP synthase subunit beta |
| A0A1D5XH30 | Triticum aestivum | 93.89 | 9 | 0.37 | V-type proton ATPase subunit a |
| M7YLU4 | Triticum aestivum | 44.71 | 14 | 0.25 | Obg-like ATPase 1 |
| M7YU16 | Triticum aestivum | 19.53 | 8 | 0.12 | ATP synthase subunit d |
| M8BLL3 | Triticum aestivum | 140.1 | 31 | 0.38 | Phosphate dikinase 1 |
| A0A1D5Z2E1 | Triticum aestivum | 89.86 | 21 | 0.04 | ATP binding |
| F2D5G5 | Triticum aestivum | 73.83 | 18 | 0.05 | ATP binding |
| W5EF54 | Triticum aestivum | 70.09 | 24 | 0.13 | ATP binding |
| A0A1D6AT12 | Triticum aestivum | 65.03 | 6 | 0.20 | ATP binding |
| A0A1D5XJM8 | Triticum aestivum | 108.6 | 23 | 0.32 | ATP binding |
| **Transport** |  |  |  |  |  |
| A0A1D6AX03 | Triticum aestivum | 45.89 | 21 | 5.63 | ADP-glucose brittle-1 transporter |
| A0A1D6DJD7 | Triticum aestivum | 45.79 | 21 | 3.01 | Protein brittle-1 |
| A0A0D3H5P2 | Triticum aestivum | 111.51 | 7 | 4.49 | Protein transporter |
| A0A1D5ZF82 | Triticum aestivum | 108.3 | 17 | 3.84 | Protein transporter |
| W5GJK4 | Triticum aestivum | 42.84 | 11 | 0.28 | Transporter |
| W5AT17 | Triticum aestivum | 99.02 | 13 | 2.45 | Transportin-1 |
| B4FSA7 | Triticum aestivum | 41.26 | 7 | 0.50 | ADP, ATP carrier protein |
| M8CNK3 | Triticum aestivum | 72.48 | 7 | 0.32 | ADP, ATP carrier protein |
| A0A1D5XZW3 | Triticum aestivum | 100.8 | 5 | 0.39 | Beta-adaptin-like protein |
| A0A1D6S344 | Triticum aestivum | 25.26 | 9 | 0.40 | Voltage-gated cation channel |
| A0A1D6ASU8 | Triticum aestivum | 74.38 | 7 | 0.20 | Transmembrane 9 superfamily member |
| M0XYC3 | Triticum aestivum | 89.37 | 7 | 0.26 | Multiple C2 and transmembrane domain-containing protein 1 |
| N1R4N7 | Triticum aestivum | 76.38 | 11 | 0.22 | Translocation SEC63-like protein |
| F2DDK6 | Hordeum vulgare | 95.96 | 8 | 2.87 | Importin subunit beta-1 |
| W5AA91 | Triticum aestivum | 58.62 | 10 | 0.49 | Importin subunit alpha |
| **Signal Transduction** | |  |  |  |  |
| A0A1D5WBE0 | Triticum aestivum | 31.93 | 9 | 14.61 | 14-3-3 protein |
| M0XMV1 | Hordeum vulgare | 29.69 | 9 | 2.44 | 14-3-3-like protein B |
| B4FHJ9 | Zea mays | 20.02 | 3 | 5.38 | GTP binding protein |
| A0A1D5TN85 | Triticum aestivum | 52.51 | 5 | 0.48 | GTP binding |
| A0A1D5SKX0 | Triticum aestivum | 22.91 | 4 | 2.57 | GTPase |
| A0A1D5SB20 | Triticum aestivum | 82.70 | 14 | 0.44 | GTPase |
| W5FDZ3 | Triticum aestivum | 71.43 | 23 | 0.44 | GTPase |
| A0A1D6CFI9 | Triticum aestivum | 98.60 | 19 | 0.27 | GTPase |
| A0A077S025 | Triticum aestivum | 90.21 | 11 | 0.27 | GTPase |
| A0A1D5WMA2 | Triticum aestivum | 89.73 | 11 | 0.15 | GTPase |
| A0A1D5ZGU3 | Triticum aestivum | 75.10 | 10 | 0.42 | GTPase |
| F6LAX4 | Triticum aestivum | 65.50 | 11 | 0.20 | Protein phosphatase 2A structural subunit |
| **Stress/ Defense** |  |  |  |  |  |
| C0LF31 | Triticum aestivum | 43.43 | 13 | 69.60 | Serpin 2 |
| A0A1D5ZBL7 | Triticum aestivum | 43.12 | 19 | 91.39 | Serpin-Z1A |
| Q9ST57 | Triticum aestivum | 43.31 | 13 | 22.59 | Serpin-Z2A |
| C0LF30 | Triticum aestivum | 43.12 | 17 | 70.82 | Serpin 1 |
| H9AXB3 | Triticum aestivum | 43.00 | 13 | 7.65 | Serpin-N3.2 |
| A0A1D6ACI6 | Triticum aestivum | 12.63 | 3 | 8.20 | Serine-type endopeptidase inhibitor |
| A0A1D5XMK2 | Triticum aestivum | 14.73 | 5 | 8.04 | Serine-type endopeptidase inhibitor |
| M8B2U4 | Triticum aestivum | 15.84 | 4 | 14.03 | Serine-type endopeptidase inhibitor |
| B5B0D5 | Triticum aestivum | 15.78 | 6 | 3.42 | Alpha-amylase inhibitor CM16 subunit |
| M8B9L0 | Triticum aestivum | 15.92 | 5 | 2.59 | Alpha-amylase/trypsin inhibitor CM16 |
| M8BV45 | Aegilops tauschii | 24.25 | 10 | 10.57 | Alpha-amylase/trypsin inhibitor CM3 |
| P17314 | Triticum aestivum | 18.22 | 9 | 10.28 | Alpha-amylase/trypsin inhibitor CM3 |
| M8A1S2 | Triticum urartu | 16.47 | 4 | 8.60 | Trypsin/alpha-amylase inhibitor CMX1/CMX3 |
| N1QTW5 | Aegilops tauschii | 15.26 | 4 | 2.94 | Trypsin inhibitor CMc |
| A0A1D5XVT4 | Triticum aestivum | 61.12 | 25 | 0.16 | Chaperonin CPN60-2 |
| A0A1D6BE72 | Triticum aestivum | 110.00 | 17 | 0.43 | Chaperone protein ClpB 2 |
| S4VQP9 | Triticum aestivum | 58.05 | 9 | 0.20 | Chaperonin family theta subunit |
| M7YWA0 | Triticum aestivum | 76.12 | 20 | 0.08 | Heat shock 70 kDa protein |
| N1R361 | Triticum aestivum | 160.0 | 17 | 0.06 | Heat shock cognate 70 kDa protein 1 |
| Q9XEI1 | Triticum aestivum | 101.1 | 21 | 0.18 | Heat shock protein 101 |
| A0A1D5V365 | Triticum aestivum | 27.66 | 8 | 0.47 | L-ascorbate peroxidase 2 |
| F2DTT4 | Triticum aestivum | 28.23 | 6 | 0.23 | Peroxiredoxin |
| Q84U03 | Triticum aestivum | 38.82 | 14 | 6.83 | Peroxidase |
| A0A1D5WD63 | Triticum aestivum | 29.50 | 6 | 4.90 | Pyrroline-5-carboxylate reductase |
| A0A1D6ALN8 | Triticum aestivum | 54.42 | 14 | 3.49 | Betaine-aldehyde dehydrogenase |
| A0A1D5URR5 | Triticum aestivum | 41.10 | 9 | 0.32 | 12-oxophytodienoate reductase 2 |
| A0A1D6CB83 | Triticum aestivum | 37.32 | 6 | 2.09 | Lactoylglutathione lyase |
| A0A060APV2 | Triticum aestivum | 8.35 | 2 | 3.39 | Defensin SD2 |
| A0A1D6CI91 | Triticum aestivum | 8.11 | 2 | 0.05 | Defensin Tk-AMP-D4 |
| A0A1D5SQ20 | Triticum aestivum | 58.02 | 12 | 0.23 | Serine hydroxymethyltransferase |
| **Nucleic acid-related** | |  |  |  |  |
| C5YYL4 | Sorghum bicolor | 16.28 | 2 | 11.96 | Histone H2A |
| A0A1D6CUB4 | Triticum aestivum | 25.31 | 4 | 9.55 | Histone H2A |
| I1H6W0 | Brachypodium distachyon | 16.92 | 2 | 6.17 | Histone H2A |
| A0A1D5Z8I6 | Triticum aestivum | 16.41 | 7 | 4.36 | Histone H4 |
| A0A1D5SHI2 | Triticum aestivum | 38.54 | 7 | 0.06 | Histone deacetylase HDAC2 |
| A0A1D5ZLD1 | Triticum aestivum | 40.66 | 8 | 4.24 | 5S rRNA binding |
| M8BIQ7 | Triticum aestivum | 45.46 | 6 | 4.94 | RNA binding |
| A0A1D5TE22 | Triticum urartu | 47.22 | 7 | 2.05 | RNA binding |
| A0A1D5UDE8 | Triticum aestivum | 51.40 | 7 | 0.11 | RNA binding |
| A0A1D5Y5R8 | Triticum aestivum | 16.11 | 4 | 0.22 | Glycine-rich RNA-binding protein |
| I1I7G2 | Triticum aestivum | 16.15 | 2 | 2.41 | 60S ribosomal protein L34 |
| A0A1D5T5A4 | Triticum aestivum | 22.21 | 4 | 3.90 | 40S ribosomal protein S7 |
| A0A0D9VE04 | Oryza barthii | 18.07 | 4 | 2.14 | 40S ribosomal protein S24 |
| M7YVE5 | Triticum urartu | 22.00 | 6 | 2.29 | 60S ribosomal protein L28-1 |
| Q9AXS0 | Triticum aestivum | 19.56 | 3 | 2.75 | Ribosomal protein L17-1 |
| R7W6B0 | Triticum aestivum | 30.41 | 10 | 2.07 | 60S ribosomal protein L7a |
| M7ZWX9 | Triticum aestivum | 12.09 | 6 | 0.20 | 60S acidic ribosomal protein P3 |
| M7YAM8 | Triticum aestivum | 18.28 | 3 | 0.46 | 40S ribosomal protein S27 |
| A0A1D6C207 | Triticum aestivum | 15.15 | 3 | 0.48 | 60S acidic ribosomal protein P1 |
| W5C5K1 | Triticum aestivum | 45.91 | 11 | 0.44 | Translation initiation factor 3 subunit M |
| D8L9U6 | Triticum aestivum | 117.15 | 19 | 0.26 | Translation initiation factor 3 subunit A |
| E6Y289 | Triticum aestivum | 18.78 | 8 | 0.48 | Translationally-controlled protein |
| A0A1D5TKE5 | Triticum aestivum | 23.39 | 9 | 0.37 | Translation elongation factor |
| A0A1D5Y115 | Triticum aestivum | 30.50 | 5 | 0.43 | Translation initiation factor |
| M8AZD7 | Triticum aestivum | 63.77 | 9 | 0.39 | Translation initiation factor 3 subunit E |
| M8C107 | Triticum aestivum | 24.94 | 6 | 0.18 | Translation initiation factor 5A-1 |
| M8C6D4 | Triticum aestivum | 90.07 | 15 | 0.22 | Eukaryotic initiation factor iso-4F subunit p82-34 |
| A0A1D5ZWW7 | Triticum aestivum | 53.06 | 11 | 0.24 | Elongation factor Tu |
| A0A1D6AAQ1 | Triticum aestivum | 50.47 | 14 | 0.02 | Elongation factor Tu |
| T1MSW5 | Triticum aestivum | 55.31 | 12 | 3.01 | Elongation factor 1-alpha |
| M7ZF90 | Triticum urartu | 59.86 | 4 | 3.09 | T-complex protein 1 subunit epsilon |
| A0A1D5VEF1 | Triticum urartu | 47.36 | 6 | 3.81 | Zinc finger protein |
| M8BN55 | Triticum aestivum | 54.68 | 6 | 0.19 | Basic leucine zipper and W2 domain-containing protein 2 |
| A0A1D5WG52 | Triticum aestivum | 23.43 | 2 | 0.20 | H/ACA ribonucleoprotein complex subunit |
| W4ZTA4 | Triticum aestivum | 51.92 | 5 | 0.39 | DEAD-box ATP-dependent RNA helicase 56 |
| Q7X7E8 | Triticum aestivum | 24.65 | 6 | 0.23 | Peptidyl-prolyl cis-trans isomerase |
| A0A1D5U8J7 | Triticum aestivum | 63.87 | 13 | 0.32 | Pseudouridine synthase |
| **Protein synthesis/assembly/degradation** | |  |  |  |  |
| A0A1D5Z1A0 | Triticum aestivum | 60.74 | 14 | 10.65 | Metalloendopeptidase |
| A0A1D5YEH0 | Triticum aestivum | 56.24 | 15 | 5.83 | Metalloendopeptidase |
| A0A1D6ABF1 | Triticum aestivum | 54.81 | 21 | 2.29 | Aspartic proteinase |
| A0A1D5VCN0 | Triticum aestivum | 53.09 | 14 | 8.88 | Aspartic proteinase。 |
| Q07810 | Triticum aestivum | 29.61 | 11 | 36.52 | rRNA N-glycosidase |
| M7ZDX9 | Triticum aestivum | 72.70 | 9 | 3.65 | Tubulin alpha-2 chain |
| A0A1D5ZXR0 | Triticum aestivum | 49.77 | 17 | 0.37 | Tubulin alpha chain |
| A0A024FRA5 | Triticum aestivum | 63.59 | 8 | 0.28 | Disulfide-isomerase |
| W5FTE3 | Triticum aestivum | 47.17 | 12 | 0.30 | Disulfide-isomerase A6 |
| M7ZN83 | Triticum aestivum | 43.58 | 10 | 0.31 | Protein disulfide isomerase-like 2-1 |
| M7YXF2 | Triticum aestivum | 52.50 | 7 | 0.09 | Disulfide isomerase-like 5-2 |
| G4XH71 | Triticum aestivum | 18.38 | 5 | 0.24 | Peptidyl-prolyl cis-trans isomerase |
| A0A1D6B557 | Triticum aestivum | 65.81 | 13 | 0.09 | Metalloexopeptidase activity |
| A0A1D5XSS6 | Triticum aestivum | 71.11 | 16 | 0.35 | Ubiquitin protein ligase |
| P20973 | Triticum aestivum | 117.01 | 18 | 0.28 | Ubiquitin-activating enzyme E1 1 |
| M8AD47 | Triticum aestivum | 77.46 | 27 | 0.50 | Luminal-binding protein 3 |
| M8A7K2 | Triticum aestivum | 49.58 | 6 | 0.16 | DnaJ protein-like protein |
| R7WC71 | Triticum aestivum | 138.2 | 28 | 0.30 | Endoplasmin-like protein |
| **Miscellaneous** |  |  |  |  |  |
| A0A1D5S2A 6 | Triticum aestivum | 52.81 | 20 | 3.42 | Pyridoxal phosphate binding |
| A0A1D5W804 | Triticum aestivum | 83.47 | 12 | 2.95 | Proline-tRNA ligase |
| A0A1D6D2N0 | Triticum aestivum | 30.40 | 7 | 2.52 | Acid phosphatase |
| M8BYD7 | Aegilops tauschii | 37.13 | 3 | 7.92 | Purple acid phosphatase 3 |
| A0A1D6C5Q0 | Triticum aestivum | 56.59 | 5 | 2.37 | Purple acid phosphatase |
| Q06I75 | Triticum aestivum | 30.34 | 4 | 0.03 | Fasciclin-like protein FLA31 |
| A0A1D6CIZ6 | Triticum aestivum | 48.53 | 6 | 0.23 | 3-oxoacyl-[acyl-carrier-protein] synthase |
| A0A1D5S1G9 | Triticum aestivum | 86.31 | 23 | 0.40 | Hydrogen-translocating pyrophosphatase |
| A0A1D5T4H8 | Triticum aestivum | 53.88 | 7 | 0.05 | Transaminase |
| A0A1D6CWR8 | Triticum aestivum | 32.44 | 9 | 0.25 | Methyltransferase |
| A0A1D6AF35 | Triticum aestivum | 39.62 | 5 | 0.09 | Methylenetetrahydrofolate dehydrogenase (NADP+) |
| A0A1D5XDV1 | Triticum aestivum | 50.29 | 13 | 0.04 | Transaminase |
| A0A1D6BBP6 | Triticum aestivum | 101.4 | 8 | 0.51 | Aminopeptidase |
| **Unknown** |  |  |  |  |  |
| A0A1D5XS09 | Triticum aestivum | 68.91 | 18 | 9.46 |  |
| W5EST8 | Triticum aestivum | 70.62 | 21 | 13.63 |  |
| A0A1D5U5J4 | Triticum aestivum | 165.0 | 27 | 2.04 |  |
| A0A1D5XVS8 | Triticum aestivum | 77.09 | 12 | 12.95 |  |
| M8B8E6 | Aegilops tauschii | 41.62 | 11 | 3.55 |  |
| W5F815 | Triticum aestivum | 25.04 | 6 | 2.87 |  |
| A0A1D6DC72 | Triticum aestivum | 22.55 | 4 | 23.92 |  |
| W5D4F5 | Triticum aestivum | 36.16 | 13 | 2.23 |  |
| M7YGR1 | Triticum aestivum | 30.97 | 3 | 2.31 |  |
| F2DBD1 | Triticum aestivum | 23.53 | 8 | 2.23 |  |
| A0A1D6RXI3 | Triticum aestivum | 25.43 | 7 | 6.57 |  |
| M8AUX2 | Triticum aestivum | 27.69 | 9 | 20.83 |  |
| W5EFT2 | Triticum aestivum | 25.15 | 9 | 4.57 |  |
| CON__P00761 | Triticum aestivum | 24.41 | 5 | 2.50 |  |
| M0YHR5 | Triticum aestivum | 54.27 | 24 | 2.11 |  |
| A0A1D5UZ29 | Triticum aestivum | 113.96 | 15 | 2.63 |  |
| A0A1D5VGX7 | Triticum aestivum | 64.80 | 21 | 3.23 |  |
| A0A1D6C0D3 | Triticum aestivum | 17.74 | 6 | 6.11 |  |
| A0A1D5X2J6 | Triticum aestivum | 18.19 | 5 | 7.12 |  |
| A0A0D3G8F9 | Oryza barthii | 18.53 | 4 | 8.11 |  |
| W4ZRU3 | Triticum aestivum | 12.35 | 3 | 4.33 |  |
| A0A1D5S0Z9 | Triticum aestivum | 63.87 | 23 | 3.25 |  |
| A0A1D5S1Q4 | Triticum aestivum | 16.11 | 4 | 8.60 |  |
| A0A1D6CKP1 | Triticum aestivum | 40.98 | 4 | 4.07 |  |
| W5G8H4 | Triticum aestivum | 81.81 | 7 | 3.01 |  |
| M0Z6T1 | Triticum aestivum | 33.42 | 5 | 20.14 |  |
| A0A1D6DKE3 | Triticum aestivum | 112.79 | 20 | 0.43 |  |
| A0A1D5WP22 | Triticum aestivum | 28.40 | 5 | 0.08 |  |
| A0A1D5YZ83 | Triticum aestivum | 21.88 | 7 | 0.03 |  |
| W5AQE7 | Triticum aestivum | 34.91 | 19 | 0.01 |  |
| A0A1D5T8I8 | Triticum aestivum | 10.71 | 5 | 0.41 |  |
| A0A1D5TZM1 | Triticum aestivum | 36.45 | 5 | 0.36 |  |
| W5DWT9 | Triticum aestivum | 37.01 | 5 | 0.40 |  |
| W5BGN8 | Triticum aestivum | 59.18 | 8 | 0.34 |  |
| A0A1D5Y6Y2 | Triticum aestivum | 72.79 | 6 | 0.31 |  |
| F2DVW0 | Triticum aestivum | 28.49 | 4 | 0.33 |  |
| W5EPT4 | Triticum aestivum | 34.66 | 3 | 0.31 |  |
| M8A2R3 | Triticum aestivum | 41.80 | 17 | 0.35 |  |
| W5EB84 | Triticum aestivum | 108.6 | 37 | 0.43 |  |
| A0A1D5YME0 | Triticum aestivum | 28.73 | 6 | 0.40 |  |
| A0A1D5S022 | Triticum aestivum | 90.86 | 6 | 0.38 |  |
| A0A1D5Y2X2 | Triticum aestivum | 34.18 | 4 | 0.24 |  |
| W5AKQ2 | Triticum aestivum | 12.08 | 4 | 0.32 |  |
| W5AZH8 | Triticum aestivum | 61.78 | 18 | 0.18 |  |
| A0A1D5SUM8 | Triticum aestivum | 64.80 | 12 | 0.04 |  |
| A0A1D6C8U9 | Triticum aestivum | 39.17 | 7 | 0.03 |  |
| M0XGG6 | Triticum aestivum | 57.52 | 10 | 0.16 |  |
| A0A1D5WLK3 | Triticum aestivum | 36.02 | 10 | 0.02 |  |
| A0A1D5YMS0 | Triticum aestivum | 29.34 | 4 | 0.16 |  |
| A0A1D6CZK2 | Triticum aestivum | 9.68 | 3 | 0.49 |  |
| A0A1D5WMS9 | Triticum aestivum | 54.73 | 6 | 0.44 |  |
| A0A1D6CDM8 | Triticum aestivum | 19.55 | 7 | 0.36 |  |
| A0A1D5X267 | Triticum aestivum | 18.89 | 4 | 0.11 |  |
| A0A1D5UBT2 | Triticum aestivum | 71.96 | 8 | 0.49 |  |
| F2D1A7 | Triticum aestivum | 17.67 | 6 | 0.42 |  |
| M8BEB8 | Triticum aestivum | 51.56 | 10 | 0.38 |  |

^a^ Accession number of the predicted protein in Uniprot.

^b^ Mr: Molecular mass of the predicted protein.

^c^ NP: Number of matched peptides.

^d^ Ratio: Ratio of the abundance of the protein identified at 15DAA to that of 10 DAA.

^e^ One way ANOVA *p* value <0.05.
