# Supplementary material for: Quantitative analysis of the grain amyloplast proteome reveals differences in metabolism between two wheat cultivars at two stages of grain development
Source: BMC Genomics. 2018 Oct 24;19:768. doi: 10.1186/s12864-018-5174-z (PMC6201562; doi:10.1186/s12864-018-5174-z)
Supplement: Supplementary file 2 — Table S2. Differentially expressed proteins identified in wheat grain amyloplasts between 10 DAA and 15 DAA in the soft wheat cultivar YM49–198. (DOCX 54 kb) [file 12864_2018_5174_MOESM2_ESM.docx]

Table S2. Differentially expressed proteins identified in wheat grain amyloplasts between 10 DAA and 15 DAA in the soft wheat cultivar YM49-198.

| **Accession No.** | | **Species** | **Mr** | **NP** | **Ratio** | **Description** |
| --- | --- | --- | --- | --- | --- | --- |
| **Carbohydrate Metabolism** | | |  |  |  |  |
| A0A1D6S517 | Triticum aestivum | | 59.70 | 19 | 0.41 | UTP:glucose-1-phosphate uridylyltransferase |
| A0A1D6RLR1 | Triticum aestivum | | 93.08 | 25 | 0.34 | Starch branching enzyme IIa |
| Q43654 | Triticum aestivum | | 71.00 | 14 | 0.03 | Starch synthase 1 |
| G3CCE7 | Triticum aestivum | | 93.01 | 25 | 0.24 | Starch branching enzyme IIa |
| A0A1D5T6Q3 | Triticum aestivum | | 72.40 | 11 | 0.04 | Starch synthase |
| A0A1D5U5L3 | Triticum aestivum | | 88.06 | 22 | 0.04 | 1,4-alpha-glucan branching enzyme |
| M8C4M4 | Triticum aestivum | | 61.76 | 8 | 2.35 | UDP-glucose 6-dehydrogenase |
| Q8W1W2 | Bambusa oldhamii | | 92.12 | 24 | 5.02 | Sucrose synthase |
| M8A0T7 | Triticum aestivum | | 92.67 | 36 | 2.89 | Sucrose synthase |
| N1R4I4 | Triticum aestivum | | 94.03 | 36 | 5.28 | Sucrose synthase |
| W5EKI0 | Triticum aestivum | | 61.11 | 23 | 5.19 | Beta-amylase |
| M8B5G5 | Triticum aestivum | | 58.75 | 20 | 9.04 | Beta-amylase |
| A0A1D5XGF3 | Triticum aestivum | | 58.73 | 23 | 5.64 | Beta-amylase |
| A0A024BKF6 | Pennisetum americanum | | 38.93 | 9 | 0.02 | Photosystem II protein D1 |
| A0A075BAM5 | Fargesia nitida | | 56.18 | 13 | 0.16 | 9POAL Photosystem II CP47 reaction center protein |
| W5BBT6 | Triticum aestivum | | 35.19 | 5 | 0.32 | Chlorophyll a-b binding protein |
| A0A1D6CFI6 | Triticum aestivum | | 32.35 | 5 | 0.24 | Chlorophyll a-b binding protein |
| A0A1D5RS51 | Triticum aestivum | | 28.20 | 8 | 0.15 | Chlorophyll a-b binding protein |
| M8ALL6 | Aegilops tauschii | | 99.15 | 26 | 0.06 | RuBisCO large subunit-binding protein subunit beta |
| W5F620 | Triticum aestivum | | 44.00 | 9 | 0.47 | Alpha-galactosidase |
| B2LXU4 | Triticum aestivum | | 108.2 | 17 | 0.46 | Alpha-1,4 glucan phosphorylase |
| A0A0Q3K692 | Brachypodium distachyon | | 58.84 | 7 | 0.16 | Acetyltransferase component of pyruvate dehydrogenase complex |
| N1R3V3 | Triticum aestivum | | 60.73 | 11 | 0.33 | Acetyltransferase component of pyruvate dehydrogenase complex |
| A0A1D6ATB6 | Triticum aestivum | | 56.16 | 7 | 0.80 | Glycosyl transferase |
| M8C9Y7 | Aegilops tauschii | | 43.02 | 13 | 0.02 | Glyceraldehyde-3-phosphate dehydrogenase |
| A0A1D6C1W7 | Triticum aestivum | | 102.6 | 10 | 0.06 | Hydrolyzing O-glycosyl |
| A0A1D5RVB4 | Triticum aestivum | | 54.76 | 13 | 0.42 | Phosphotransferase |
| P12782 | Triticum aestivum | | 49.84 | 21 | 0.03 | Phosphoglycerate kinase |
| W5H4V7 | Triticum aestivum | | 42.12 | 20 | 0.47 | Phosphoglycerate kinase |
| A0A1D5WKF4 | Triticum aestivum | | 63.65 | 17 | 0.46 | Phosphoglycerate mutase |
| A9U8F9 | Triticum aestivum | | 41.10 | 11 | 3.43 | Alcohol dehydrogenase ADH3D |
| A0A1D6CIT1 | Triticum aestivum | | 41.36 | 8 | 4.05 | Aldose 1-epimerase |
| A0A1D6D888 | Triticum aestivum | | 28.08 | 8 | 2.46 | Carbonic anhydrase |
| A0A0U4FQJ0 | Triticum monococcum | | 57.77 | 23 | 2.92 | Glucose-1-phosphate adenylyltransferase |
| A0A1D6D1Q3 | Triticum aestivum | | 60.56 | 21 | 3.61 | Pyrophosphate--fructose 6-phosphate 1-phosphotransferase subunit beta |
| A0A1D5YUK3 | Triticum aestivum | | 67.60 | 19 | 5.96 | Pyrophosphate--fructose 6-phosphate 1-phosphotransferase subunit alpha |
| A0A1D5WP73 | Triticum aestivum | | 54.44 | 10 | 8.49 | Pectin acetylesterase |
| A0A1D5UPJ6 | Triticum aestivum | | 55.43 | 14 | 2.03 | Pyruvate kinase |
| A0A1B1V4Q3 | Triticum aestivum | | 36.55 | 16 | 2.71 | Glyceraldehyde-3-phosphate dehydrogenase |
| A0A1D6DBW7 | Triticum aestivum | | 39.55 | 9 | 0.27 | Pyruvate dehydrogenase E1 component subunit beta |
| A0A1D6S2S6 | Triticum aestivum | | 57.42 | 17 | 3.17 | Pyruvate kinase |
| B2ZGK8 | Triticum aestivum | | 254.8 | 40 | 0.09 | Acetyl-CoA carboxylase |
| N1QTA2 | Triticum aestivum | | 56.75 | 10 | 0.15 | Citrate synthase |
| A0A1D5WP39 | Hordeum vulgare | | 51.82 | 9 | 0.03 | D-glyceraldehyde-3-phosphate glyceronetransferase |
| M8C5C6 | Triticum aestivum | | 58.39 | 15 | 0.13 | Dihydrolipoyl dehydrogenase |
| M7YGW3 | Aegilops tauschii | | 54.46 | 4 | 0.36 | Dihydroorotate dehydrogenase |
| W5AN92 | Triticum aestivum | | 62.13 | 8 | 0.06 | Dihydrolipoyl dehydrogenase |
| F2D8A6 | Triticum aestivum | | 39.76 | 9 | 0.46 | Isocitrate dehydrogenase [NAD] subunit, |
| A0A1D6CIZ6 | Triticum aestivum | | 48.53 | 6 | 0.22 | 3-oxoacyl-[acyl-carrier-protein] synthase |
| D2KZ12 | Triticum aestivum | | 47.93 | 12 | 0.32 | 3-ketoacyl-CoA thiolase-like protein |
| A0A1D5RY74 | Triticum aestivum | | 35.53 | 10 | 0.10 | Malate dehydrogenase |
| W5A4W9 | Triticum aestivum | | 41.73 | 10 | 0.12 | L-malate dehydrogenase |
| A0A1D5Y5T3 | Triticum aestivum | | 70.11 | 17 | 0.44 | Phosphotransferases |
| A0A1D5X6Q1 | Triticum aestivum | | 54.09 | 7 | 0.06 | 3-phosphoshikimate 1-carboxyvinyltransferase |
| A0A1D5YNX6 | Triticum aestivum | | 68.32 | 9 | 0.28 | Succinate dehydrogenase [ubiquinone] flavoprotein subunit |
| M7Z323 | Aegilops tauschi | | 40.33 | 8 | 0.04 | Succinyl-CoA ligase [ADP-forming] subunit alpha |
| **N Metabolism** | | |  |  |  |  |
| W5GE58 | Triticum aestivum | | 47.33 | 9 | 0.49 | Aspartate aminotransferase |
| W5B8D7 | Triticum aestivum | | 56.74 | 11 | 0.19 | Glutamate dehydrogenase |
| S5YTU4 | Triticum turgidum | | 235.1 | 20 | 15.65 | NADH-dependent glutamate synthase |
| W4ZT78 | Triticum aestivum | | 52.13 | 12 | 0.19 | Argininosuccinate synthase |
| W5DWT9 | Triticum aestivum | | 37.01 | 5 | 0.09 | Ketol-acid reductoisomerase |
| A0A1D6S7W1 | Triticum aestivum | | 56.08 | 7 | 0.37 | Acetolactate synthase |
| M7Z423 | Triticum urartu | | 125.7 | 26 | 0.05 | Carbamoyl-phosphate synthase large chain |
| A0A096UK97 | Triticum aestivum | | 54.34 | 15 | 0.21 | Aspartic-type endopeptidase |
| M7ZWM8 | Triticum urartu | | 39.07 | 9 | 4.76 | Protein serine/threonine phosphatase |
| F2D4K4 | Aegilops tauschii | | 108.5 | 5 | 2.91 | GMP synthase (Glutamine-hydrolyzing) |
| W5AC96 | Triticum aestivum | | 52.14 | 11 | 6.49 | Carboxypeptidase |
| **Energetics-related** | | |  |  |  |  |
| U5TXC9 | | Aegilops speltoides | 26.18 | 5 | 0.11 | Cytochrome b6 |
| M0WTH3 | | Triticum aestivum | 27.24 | 13 | 0.01 | Oxygen-evolving enhancer protein 2, |
| A0A1D6SDU1 | | Triticum aestivum | 74.32 | 22 | 0.34 | ATP binding |
| A0A191TDI3 | | Hordeum vulgare | 55.31 | 18 | 0.21 | ATP synthase subunit alpha |
| F2VQK3 | | Triticum aestivum | 21.62 | 5 | 0.21 | ATP synthase protein MI25 |
| A8Y9G7 | | Lolium multiflorum | 55.74 | 20 | 0.03 | ATP synthase subunit alpha, |
| A0A1D5Z2E1 | | Triticum aestivum | 89.86 | 21 | 0.03 | ATP binding |
| W5GD60 | | Triticum aestivum | 63.64 | 17 | 0.10 | ATP binding |
| A0A1D5ZRE6 | | Triticum aestivum | 61.76 | 19 | 0.04 | ATP binding |
| W5EF54 | | Triticum aestivum | 70.09 | 24 | 0.05 | ATP binding |
| M8AIK4 | | Triticum aestivum | 104.9 | 25 | 0.15 | Plasma membrane ATPase |
| A0A1D5RP45 | | Triticum aestivum | 63.53 | 20 | 0.19 | ATP synthase subunit beta |
| A0A0K0YH82 | | Hordeum vulgare | 44.45 | 7 | 0.31 | NADH dehydrogenase subunit 7 |
| A0A1D5T2S6 | | Triticum aestivum | 35.44 | 10 | 0.20 | Proton-transporting ATP synthase |
| B1A0V1 | | Bambusa oldhamii | 36.07 | 3 | 0.10 | NADH-ubiquinone oxidoreductase chain 1 |
| M7ZLJ5 | | Triticum urartu | 54.68 | 13 | 0.17 | NADH dehydrogenase |
| Q35322 | | Oryza sativa | 22.99 | 6 | 0.42 | NADH dehydrogenase [ubiquinone] iron-sulfur protein 3 |
| A0A1D5XMV6 | | Triticum aestivum | 19.52 | 5 | 0.23 | Putative NADH dehydrogenase (Ubiquinone) 1 alpha subcomplex subunit 5 |
| A0A1D5SCG8 | | Triticum aestivum | 97.56 | 12 | 3.76 | AMP deaminase |
| A0A1D6AHD7 | | Triticum aestivum | 50.03 | 24 | 4.07 | ATP binding |
| **Transport** | |  |  |  |  |  |
| A0A1D6AX03 | | Triticum aestivum | 45.89 | 21 | 3.59 | ADP-glucose brittle-1 transporter |
| A0A1D6DJD7 | | Triticum aestivum | 45.79 | 21 | 3.18 | Protein brittle-1 |
| W5GJK4 | | Triticum aestivum | 42.84 | 11 | 0.34 | Transporter |
| A0A1D5TIE5 | | Triticum aestivum | 30.37 | 5 | 0.30 | Transporter |
| A0A1D5ZF82 | | Triticum aestivum | 108.3 | 17 | 13.86 | protein transporter |
| T1MZT0 | | Aegilops tauschii | 103.1 | 13 | 3.69 | Protein transport protein Sec24-like CEF |
| B4FSA7 | | Zea mays | 41.26 | 7 | 0.23 | ADP, ATP carrier protein |
| M8A2G0 | | Triticum urartu | 41.40 | 11 | 0.35 | ADP, ATP carrier protein |
| A0A1D6ASU8 | | Triticum aestivum | 74.38 | 7 | 0.21 | Transmembrane 9 superfamily member |
| A0A1D5ZTV6 | | Triticum aestivum | 73.31 | 5 | 0.38 | Transmembrane 9 superfamily member |
| A0A1D5XZP7 | | Triticum aestivum | 22.50 | 6 | 0.24 | Hydrogen ion transmembrane transporter |
| M7YUE3 | | Hordeum vulgare | 21.52 | 3 | 0.33 | Hydrogen ion transmembrane transporter |
| M8BZ92 | | Aegilops tauschii | 29.46 | 9 | 0.40 | Outer membrane protein porin of 34 kDa |
| A0A1D6S344 | | Triticum aestivum | 25.26 | 9 | 0.46 | Voltage-gated cation channel |
| A0A1D5V5E7 | | Hordeum vulgare | 29.90 | 5 | 0.29 | Voltage-gated anion channel |
| M7ZFX1 | Triticum urartu | | 64.60 | 9 | 0.21 | Outer membrane porin |
| M7ZDS1 | | Aegilops tauschii | 32.69 | 10 | 0.26 | 2-oxoglutarate/malate carrier protein |
| M7Z5I7 | | Triticum rartu | 32.34 | 7 | 0.16 | Uncoupling protein 3 |
| A7J2I3 | | Triticum aestivum | 29.89 | 4 | 0.49 | membrane intrinsic protein |
| F2DDK6 | | Triticum urartu | 95.96 | 8 | 2.53 | Importin subunit beta-1 |
| W5AA91 | | Triticum aestivum | 58.62 | 10 | 3.04 | Importin subunit alpha |
| W5D5P8 | | Triticum aestivum | 105.3 | 13 | 2.07 | Coatomer subunit beta |
| A0A1D5VXC3 | | Triticum aestivum | 58.95 | 14 | 8.96 | Importin subunit alpha |
| A0A1D5XKY8 | | Triticum aestivum | 135.8 | 17 | 3.48 | Coatomer subunit alpha |
| A0A1D5SUU2 | | Triticum aestivum | 56.64 | 9 | 14.37 | Coatomer subunit delta |
| M8AQF7 | | Triticum aestivum | 195.8 | 42 | 2.50 | Clathrin heavy chain |
| M7YTD3 | | Triticum aestivum | 35.33 | 11 | 4.88 | Annexin |
| **Signal Transduction** | | |  |  |  |  |
| A0A1D5WBE0 | | Triticum aestivum | 31.93 | 9 | 6.69 | 14-3-3 protein |
| M0XMV1 | | Triticum urartu | 29.69 | 9 | 2.70 | 14-3-3-like protein B |
| P29305 | | Triticum aestivum | 29.35 | 12 | 3.55 | 14-3-3-like protein A |
| M7ZQ50 | | Triticum rartu | 28.51 | 9 | 4.04 | GTP-binding protein SAR1A |
| A0A1D5YT34 | | Triticum aestivum | 22.46 | 5 | 2.28 | Small GTP-binding protein |
| A0A1D5SB20 | | Triticum aestivum | 82.70 | 14 | 0.47 | GTPase |
| W5FDZ3 | | Triticum aestivum | 71.43 | 23 | 0.39 | Rho GTPase |
| A0A1D6CLA6 | | Triticum aestivum | 68.79 | 11 | 0.35 | Cell elongation protein DIMINUTO |
| P04464 | | Triticum aestivum | 16.85 | 7 | 0.29 | Calmodulin |
| **Stress/Defense** | |  |  |  |  |  |
| C0LF31 | | Triticum aestivum | 43.43 | 13 | 22.08 | Serpin 2 |
| C0LF30 | | Triticum aestivum | 43.12 | 17 | 10.69 | Serpin 1 |
| A0A1D5ZBL7 | | Triticum aestivum | 43.12 | 19 | 6.48 | Serpin-Z1A |
| H9AXB3 | | Triticum aestivum | 43.00 | 13 | 11.71 | Serpin-N3.2 |
| Q9ST57 | | Triticum aestivum | 43.31 | 13 | 2.84 | Serpin-Z2A |
| A0A1D6ACI6 | | Triticum aestivum | 12.63 | 3 | 2.80 | Serine-type endopeptidase inhibitor |
| A0A1D5XMK2 | | Triticum aestivum | 14.73 | 5 | 2.03 | Serine-type endopeptidase inhibitor |
| M8BAK8 | | Aegilops tauschii | 16.35 | 4 | 4.65 | Serine-type endopeptidase inhibitor |
| P17314 | | Triticum aestivum | 18.22 | 9 | 3.48 | Alpha-amylase/trypsin inhibitor CM3 |
| Q7M219 | | Triticum turgidum | 2.39 | 1 | 2.95 | Alpha-amylase inhibitor |
| M8BV45 | | Aegilops tauschii | 24.25 | 10 | 2.73 | Alpha-amylase/trypsin inhibitor CM3 |
| M8B9L0 | | Aegilops tauschii | 15.92 | 5 | 2.48 | Alpha-amylase/trypsin inhibitor CM16 |
| A0A1D6B7P2 | | Triticum aestivum | 24.97 | 2 | 3.00 | Alpha-amylase/trypsin inhibitor |
| W5D003 | | Triticum aestivum | 16.48 | 9 | 3.65 | Dimeric alpha-amylase inhibitor |
| A0A1D5XVT4 | | Triticum aestivum | 61.12 | 25 | 0.19 | Chaperonin CPN60-2 |
| M7YWA0 | | Triticum urartu | 76.12 | 20 | 0.01 | Heat shock 70 kDa protein, |
| M8A9J7 | | Triticum aestivum | 70.63 | 11 | 0.43 | Catalase |
| M8CVR1 | | Triticum aestivum | 27.42 | 6 | 0.23 | Superoxide dismutase |
| A0A1D6BF22 | | Triticum aestivum | 56.20 | 4 | 0.31 | Peroxidase |
| R7W2K4 | | Triticum aestivum | 10.98 | 2 | 0.18 | Ozone-responsive stress-related protein |
| R7VZR7 | | Triticum aestivum | 58.00 | 9 | 0.08 | Serine hydroxymethyltransferase |
| M7YVS8 | Triticum aestivum | | 15.28 | 4 | 11.71 | Profilin |
| A0A1D6CG26 | Triticum aestivum | | 14.18 | 4 | 10.58 | Profilin |
| **Nucleic acid-related** | | |  |  |  |  |
| A0A1D5XC95 | Triticum aestivum | | 16.47 | 4 | 3.72 | Histone H2A |
| A0A1D5Z8I6 | Triticum aestivum | | 16.41 | 7 | 8.19 | Histone H4 |
| M8AA65 | Triticum urartu | | 11.37 | 7 | 2.72 | Histone H4 |
| A0A1D5SHI2 | Triticum aestivum | | 38.54 | 7 | 0.07 | Histone deacetylase HDT1 |
| A0A1D6D433 | Triticum aestivum | | 48.53 | 5 | 0.23 | RNA binding |
| A0A1D5XRK1 | Triticum aestivum | | 52.99 | 9 | 0.39 | RNA binding |
| A0A1D5UDE8 | Triticum aestivum | | 51.40 | 7 | 0.13 | RNA binding |
| A0A1D6C4M4 | Triticum aestivum | | 30.95 | 13 | 2.64 | RNA binding |
| A0A1D5ZLD1 | Triticum aestivum | | 40.66 | 8 | 2.51 | RNA binding |
| F2DRK6 | Triticum urartu | | 25.47 | 9 | 2.96 | RNA binding |
| A0A0E0GTI4 | Oryza nivara | | 37.06 | 5 | 2.21 | DNA binding |
| M7ZWX9 | Triticum urartu | | 12.09 | 6 | 0.19 | 60S acidic ribosomal protein P3 |
| A0A1D5XDZ3 | Triticum aestivum | | 40.79 | 10 | 2.82 | 40S ribosomal protein |
| M8BDF7 | Triticum aestivum | | 17.48 | 4 | 6.34 | 40S ribosomal protein S12 |
| A0A1D5U933 | Triticum aestivum | | 30.71 | 7 | 2.27 | 40S ribosomal protein S6 |
| R7W4X4 | Aegilops tauschii | | 16.73 | 3 | 3.69 | 60S ribosomal protein L36 |
| M0XW49 | Triticum urartu | | 22.49 | 9 | 2.60 | 40S ribosomal protein S9-2 |
| A0A1D5RU82 | Triticum aestivum | | 25.55 | 7 | 2.91 | 60S ribosomal protein L18a |
| M8AW09 | Triticum urartu | | 16.45 | 7 | 6.45 | 40S ribosomal protein S17-4 |
| A0A1D5YTD0 | Triticum urartu | | 29.82 | 7 | 5.31 | 60S ribosomal protein L8 |
| A0A1D5T5A4 | Triticum aestivum | | 22.21 | 4 | 4.92 | 40S ribosomal protein S7 |
| M7Z8T0 | Triticum urartu | | 22.68 | 7 | 2.24 | 40S ribosomal protein S9-2 |
| R7WDC3 | Aegilops tauschii | | 31.44 | 4 | 2.53 | 60S ribosomal protein L31 |
| M7YXD5 | Triticum aestivum | | 19.06 | 3 | 2.42 | 40S ribosomal protein S24 |
| Q9AXS0 | Poa secunda | | 19.56 | 3 | 2.80 | Ribosomal protein L17-1 |
| A0A1D6AAQ1 | Triticum aestivum | | 50.47 | 14 | 0.04 | Elongation factor Tu |
| A0A1D5ZWW7 | Triticum aestivum | | 53.06 | 11 | 0.09 | Elongation factor Tu |
| A0A1D5TKE5 | Triticum aestivum | | 23.39 | 9 | 3.07 | translation elongation factor |
| M8CZY8 | Triticum aestivum | | 104.8 | 15 | 2.83 | translation initiation factor 3 subunit |
| N1QPV1 | Triticum aestivum | | 18.57 | 6 | 5.99 | Alpha-2-purothionin |
| B8YM21 | Triticum urartu | | 14.64 | 6 | 15.75 | Beta purothionin |
| Q9T0P1 | Triticum aestivum | | 14.63 | 5 | 12.39 | Alpha purothionin |
| A0A1D5WG52 | Triticum aestivum | | 23.43 | 2 | 0.29 | H/ACA ribonucleoprotein complex subunit |
| Q6XPZ4 | Triticum aestivum | | 25.89 | 6 | 0.08 | Peptidyl-prolyl cis-trans isomerase |
| A0A1D5U8J7 | Triticum aestivum | | 63.87 | 13 | 0.23 | Pseudouridine synthase |
| **Protein Synthesis/Assembly/Degradation** | | | | | | |
| A0A1D6RW70 | Triticum aestivum | | 58.52 | 17 | 0.33 | Metalloendopeptidase |
| M7ZN83 | Triticum urartu | | 43.58 | 10 | 0.14 | Disulfide isomerase-like 2-1 |
| M8A7K2 | Triticum urartu | | 49.58 | 6 | 0.45 | DnaJ protein-like protein |
| M8AD47 | Triticum urartu | | 77.46 | 27 | 0.35 | Luminal-binding protein 3 |
| R7WC71 | Aegilops tauschii | | 138.2 | 28 | 0.30 | Endoplasmin-like protein |
| A0A1D5YEH0 | Triticum aestivum | | 56.24 | 15 | 2.24 | Metalloendopeptidase |
| A0A1D5X267 | Triticum aestivum | | 18.89 | 4 | 2.82 | Ubiquitin-dependent protein catabolic |
| M8C6C1 | Aegilops tauschii | | 44.38 | 7 | 3.40 | Aspartic proteinase Asp1 |
| A0A1D5VCN0 | Triticum aestivum | | 53.09 | 14 | 2.07 | Aspartic proteinase oryzasin-1 |
| M7ZDX9 | Triticum urartu | | 72.70 | 9 | 3.72 | Tubulin alpha-2 chain |
| Q07810 | Triticum aestivum | | 29.61 | 11 | 15.04 | rRNA N-glycosidase |
| A0A1D5VIJ5 | Triticum aestivum | | 37.07 | 4 | 3.92 | 26s proteasome non-ATPase regulatory subunit |
| **Miscellaneous** |  | |  |  |  |  |
| A0A1D5S1G9 | Triticum aestivum | | 86.31 | 23 | 0.18 | Hydrogen-translocating pyrophosphatase |
| A0A1D5RS02 | Triticum aestivum | | 52.32 | 9 | 0.48 | Tocopherol cyclase |
| A0A1D5UG29 | Triticum aestivum | | 57.15 | 15 | 0.18 | Methylmalonate-semialdehyde dehydrogenase |
| A0A1D6AF35 | Triticum aestivum | | 39.62 | 5 | 0.17 | Methylenetetrahydrofolate dehydrogenase (NADP+ |
| A0A1D6C4W3 | Triticum aestivum | | 72.92 | 17 | 0.02 | Cell division protease ftsH-like protein |
| A0A1D5Z0I6 | Triticum aestivum | | 76.44 | 17 | 0.19 | Formate-tetrahydrofolate ligase |
| A0A1D6CWR8 | Triticum aestivum | | 32.44 | 9 | 0.17 | Methyltransferase |
| CON__P02533 | Triticum aestivum | | 51.62 | 10 | 0.13 | Keratin type I cytoskeletal 14 |
| A0A1D6DB09 | Triticum aestivum | | 44.30 | 7 | 0.20 | Fasciclin-like protein FLA5 |
| A0A1D5S2A6 | Triticum aestivum | | 52.81 | 20 | 3.69 | Pyridoxal phosphate binding |
| A0A1D6ALN8 | Triticum aestivum | | 54.42 | 14 | 4.42 | Betaine-aldehyde dehydrogenase |
| A0A1D5W804 | Triticum aestivum | | 83.47 | 12 | 2.32 | Proline-tRNA ligase |
| R7W4N8 | Aegilops tauschii | | 46.97 | 6 | 0.43 | Putative acetyl-CoA acetyltransferase |
| **Unknown** |  | |  |  |  |  |
| A0A1D5WP22 | Triticum aestivum | | 28.40 | 5 | 0.11 |  |
| W5AQE7 | Triticum aestivum | | 34.91 | 19 | 0.02 |  |
| A0A1D6CWE1 | Triticum aestivum | | 21.89 | 6 | 0.44 |  |
| W5EPT4 | Triticum aestivum | | 34.66 | 3 | 0.21 |  |
| A0A1D5YNL5 | Triticum aestivum | | 34.15 | 4 | 0.38 |  |
| A0A1D5WHU0 | Triticum aestivum | | 41.51 | 3 | 0.37 |  |
| F2DVW0 | Triticum aestivum | | 28.49 | 4 | 0.28 |  |
| F2DTT4 | Triticum aestivum | | 28.23 | 6 | 0.18 |  |
| F2D5T2 | Triticum aestivum | | 27.59 | 4 | 0.45 |  |
| M7YF45 | Triticum aestivum | | 37.52 | 4 | 0.42 |  |
| A0A1D6DKE3 | Triticum aestivum | | 112.79 | 20 | 0.29 |  |
| A0A1D5YGS5 | Triticum aestivum | | 31.27 | 6 | 0.24 |  |
| A0A1D5YME0 | Triticum aestivum | | 28.73 | 6 | 0.19 |  |
| A0A1D6ANT1 | Triticum aestivum | | 39.42 | 11 | 0.16 |  |
| A0A1D5VLL5 | Triticum aestivum | | 53.44 | 14 | 0.36 |  |
| W5BGL0 | | Triticum aestivum | 28.29 | 6 | 0.36 |  |
| A0A1D6AQF5 | | Triticum aestivum | 44.68 | 11 | 0.18 |  |
| A0A0D3H5P2 | | Triticum aestivum | 111.51 | 7 | 0.33 |  |
| A0A0E0M8I0 | | Triticum aestivum | 31.91 | 7 | 0.44 |  |
| A0A1D5RP81 | | Triticum aestivum | 67.41 | 13 | 0.17 |  |
| M8BEB8 | | Triticum aestivum | 51.56 | 10 | 0.16 |  |
| R7W586 | | Triticum aestivum | 23.98 | 6 | 0.70 |  |
| W5AZH8 | | Triticum aestivum | 61.78 | 18 | 0.14 |  |
| I1IEP9 | | Triticum aestivum | 17.85 | 8 | 0.31 |  |
| A0A1D5UZ29 | | Triticum aestivum | 113.96 | 15 | 3.79 |  |
| A0A1D5SUV6 | | Triticum aestivum | 53.15 | 6 | 2.41 |  |
| A0A1D6DC72 | | Triticum aestivum | 22.55 | 4 | 11.16 |  |
| M0XEJ4 | | Triticum aestivum | 95.70 | 16 | 2.62 |  |
| A0A077RU59 | | Triticum aestivum | 102.4 | 7 | 5.87 |  |
| W5G8H4 | | Triticum aestivum | 81.81 | 7 | 2.88 |  |
| A0A1D6RVM9 | | Triticum aestivum | 86.30 | 11 | 2.15 |  |
| M0YHR5 | | Triticum aestivum | 54.27 | 24 | 4.13 |  |
| M8B8E6 | | Triticum aestivum | 41.62 | 11 | 10.74 |  |
| W5F815 | | Triticum aestivum | 25.04 | 6 | 6.07 |  |
| A0A1D6CKP1 | | Triticum aestivum | 40.98 | 4 | 2.37 |  |
| A0A1D6RXI3 | | Triticum aestivum | 25.43 | 7 | 29.35 |  |
| F2D9E0 | | Triticum aestivum | 27.60 | 8 | 5.29 |  |
| A0A1D5WJB3 | | Triticum aestivum | 54.03 | 14 | 3.86 |  |
| W4ZRU3 | | Triticum aestivum | 12.35 | 3 | 3.24 |  |
| A0A1D5T1I6 | | Triticum aestivum | 29.97 | 3 | 3.55 |  |
| M7YXZ5 | | Triticum aestivum | 25.34 | 5 | 5.86 |  |
| A0A1D5Z4H9 | | Triticum aestivum | 16.10 | 6 | 4.55 |  |
| A0A1D5UWB7 | | Triticum aestivum | 36.58 | 7 | 4.02 |  |
| A0A1D5U5J4 | | Triticum aestivum | 165.0 | 27 | 6.20 |  |
| A0A1D6BAS3 | | Triticum aestivum | 42.45 | 4 | 2.60 |  |
| A0A1D6CDM8 | | Triticum aestivum | 19.55 | 7 | 4.15 |  |
| M8AUX2 | | Triticum aestivum | 27.69 | 9 | 5.08 |  |
| A0A1D5S1Q4 | | Triticum aestivum | 16.11 | 4 | 5.30 |  |
| A0A1D5YNZ7 | | Triticum aestivum | 12.19 | 3 | 2.44 |  |

^a^ Accession number of the predicted protein in Uniprot.

^b^ Mr: Molecular mass of the predicted protein.

^c^ NP: Number of matched peptides.

^d^ Ratio: Ratio of the abundance of the protein identified at 15DAA to that of 10 DAA.

^e^ One way ANOVA *p* value <0.05.
