# Supplementary material for: Quantitative analysis of the grain amyloplast proteome reveals differences in metabolism between two wheat cultivars at two stages of grain development
Source: BMC Genomics. 2018 Oct 24;19:768. doi: 10.1186/s12864-018-5174-z (PMC6201562; doi:10.1186/s12864-018-5174-z)
Supplement: Supplementary file 3 — Table S3. Cultivar-specific expressed proteins identified in wheat grain amyloplasts of cultivars ZM366 (I) and YM49–198 (II) at 10 DAA. (DOCX 42 kb) [file 12864_2018_5174_MOESM3_ESM.docx]

Table S3. Cultivar-specific expression proteins identified in wheat grain amyloplasts of cultivars ZM366 (I) and YM49-198 (II) at 10 DAA.

| **I** | | | |  |  |  |  |  |
| --- | --- | --- | --- | --- | --- | --- | --- | --- |
| **Accession No.** | | | | **Species** | **Mr** | **NP** | **Intensity** | **Description** |
| **N Metabolism** | | | | |  |  |  |  |
| W5BEG0 | | | Triticum aestivum | | 25.80 | 2 | 1.93E+07 | uminoacyl-tRNA hydrolase |
| A0A1D6B8T7 | | | Triticum aestivum | | 61.38 | 5 | 1.86E+07 | serine-type endopeptidase |
| A0A0E0K688 | | | Oryza punctata | | 61.67 | 6 | 6.42E+07 | aminopeptidase |
| M7ZKX9 | | | Triticum urartu | | 44.78 | 10 | 2.33E+07 | Glutathione S-transferase DHAR2 |
| M0XNT1 | | | Triticum aestivum | | 48.57 | 3 | 5.66E+07 | S-adenosylmethionine synthase |
| **Carbohydrate Metabolism** | | | | |  |  |  |  |
| A0A077RTG8 | | | Triticum aestivum | | 35.45 | 12 | 1.78E+07 | Malate dehydrogenase |
| A0A1D5UU24 | | | Triticum aestivum | | 52.36 | 5 | 5.75E+07 | Dolichyl-diphosphooligosaccharide--protein glycosyltransferase subunit 1 |
| M8B0G6 | | | Aegilops tauschii | | 71.30 | 4 | 2.97E+07 | Pyruvate kinase |
| A0A1D5ZD42 | | | Triticum aestivum | | 67.60 | 16 | 2.66E+07 | Glucose-6-phosphate isomerase |
| I1HA00 | | | Brachypodium distachyon | | 106.8 | 9 | 1.97E+07 | Aconitate hydratase |
| M7ZQ92 | | | Triticum urartu | | 41.07 | 7 | 6.83E+06 | 12-oxophytodienoate reductase 2 |
| M7ZVJ8 | | | Triticum urartu | | 62.42 | 6 | 4.08E+07 | glucose-regulated protein-like protein |
| Q5PXV6 | | | Psathyrostachys fragilis | | 23.32 | 2 | 1.15E+07 | Beta-amylase |
| I1IKR4 | | | Brachypodium distachyon | | 46.23 | 3 | 1.44E+07 | GDP-mannose 3,5-epimerase |
| A0A1D6DBC3 | | | Triticum aestivum | | 76.44 | 8 | 2.62E+07 | UDP-glucose 4,6-dehydratase |
| F2CR08 | | | Triticum aestivum | | 48.06 | 20 | 9.04E+06 | phosphopyruvate hydratase |
| A0A1D5YPC2 | | | Triticum aestivum | | 67.38 | 6 | 1.06E+07 | hydrolyzing O-glycosyl |
| A0A1D5ZTS6 | | | Triticum aestivum | | 34.87 | 3 | 3.76E+07 | 6-phosphogluconolactonase |
| A0A1D6AIH4 | | | Triticum aestivum | | 55.16 | 10 | 3.31E+07 | 3-isopropylmalate dehydratase |
| **Energetics-related** | | | | |  |  |  |  |
| B6UZ91 | | | Triticum aestivum | | 28.22 | 2 | 3.66E+07 | Ferritin |
| M8A5N8 | | | Triticum aestivum | | 45.37 | 4 | 3.55E+07 | 90 kDa heat shock protein ATPase-like protein 1 |
| B2B9U2 | | | Triticum aestivum | | 12.39 | 3 | 1.13E+07 | acuolar ATPase subunit G |
| A0A1D6DIM7 | | | Triticum aestivum | | 44.34 | 14 | 2.20E+07 | Obg-like ATPase 1 |
| A0A0E0LFQ3 | | | Oryza punctate | | 84.34 | 13 | 2.04E+07 | ATPase |
| A0A1D6BXB7 | | | Triticum aestivum | | 34.60 | 4 | 2.31E+07 | magnesium protoporphyrin IX methyltransferase |
| A0A1D6C872 | | | Triticum aestivum | | 92.97 | 27 | 1.06E+07 | ATPase activity |
| **Transport** | | | | |  |  |  |  |
| M7Z3Q1 | | | Triticum urartu | | 20.84 | 5 | 4.76E+06 | Outer membrane lipoprotein blc |
| A0A1D5YDP9 | | | Triticum aestivum | | 41.04 | 4 | 4.01E+07 | Protein brittle-1 |
| A0A1D5Y2T2 | | | Triticum aestivum | | 135.9 | 17 | 1.63E+07 | Coatomer subunit alpha |
| A0A1D5TK37 | | | Triticum aestivum | | 89.93 | 14 | 2.40E+07 | protein transporter |
| A0A1D5UD87 | | | Triticum aestivum | | 17.00 | 2 | 2.55E+07 | lipid transport |
| A0A1D5Z009 | | | Triticum aestivum | | 24.39 | 4 | 1.26E+08 | vacuolar transport |
| A0A1D5Z640 | | | Triticum aestivum | | 11.15 | 2 | 3.70E+07 | Protein transport protein Sec61 subunit beta |
| M7Z0Y3 | | | Triticum aestivum | | 28.38 | 2 | 1.60E+07 | Vesicle-associated protein 1-3 |
| **Signal Transduction** | | | | |  |  |  |  |
| A0A1D5SBW4 | | | Triticum aestivum | | 56.85 | 3 | 3.97E+07 | GTPase activator |
| I7JI43 | | | Triticum aestivum | | 16.19 | 2 | 1.98E+07 | Histidine-containing phosphotransfer protein 3 |
| **Stress/Defense** | | |  | |  |  |  |  |
| M7YJV0 | | | | Triticum urartu | 82.47 | 8 | 7.97E+07 | Delta-1-pyrroline-5-carboxylate synthase |
| A0A1D5WD63 | | | | Triticum aestivum | 29.50 | 6 | 3.43E+07 | Pyrroline-5-carboxylate reductase |
| A0A097KUG3 | | | | Triticum aestivum | 16.80 | 4 | 1.56E+07 | Actin-depolymerizing factor 6 |
| M7YRR2 | | | | Triticum urartu | 21.46 | 6 | 2.00E+07 | Actin-depolymerizing factor 4 |
| A0A0C4BIK9 | | | | Triticum aestivum | 40.07 | 6 | 5.05E+07 | Farnesyl pyrophosphate synthase A2 |
| L7S310 | | | | Triticum aestivum | 40.09 | 7 | 9.95E+07 | Farnesyl pyrophosphate synthase B2 |
| M7YAG3 | | | | Triticum aestivum | 38.42 | 4 | 6.17E+06 | lactoylglutathione lyase, |
| M8BMF3 | | | | Triticum aestivum | 36.31 | 2 | 6.43E+06 | Autophagy-related protein 3 |
| A0A1D5TTP6 | | | | Triticum aestivum | 36.65 | 3 | 2.30E+07 | Peroxidase |
| A0A1D5XAK8 | | | | Triticum aestivum | 21.37 | 3 | 6.37E+07 | Glutathione peroxidase |
| A0A1D5XW56 | | | | Triticum aestivum | 71.82 | 18 | 3.50E+07 | Heat shock cognate 70 kDa protein |
| G9I6G6 | | | | Triticum aestivum | 18.41 | 5 | 4.63E+07 | Glutathione peroxidase |
| F2CTU5 | | | | Triticum aestivum | 35.69 | 5 | 4.37E+07 | Peroxidase |
| A0A1D5YHN5 | | | | Triticum aestivum | 25.06 | 5 | 7.84E+06 | Wali7 protein |
| A0A1D6CM14 | | | | Triticum aestivum | 28.40 | 8 | 1.06E+08 | Basic endochitinase C |
| M7YQT7 | | | | Triticum urartu | 36.30 | 4 | 4.12E+07 | Dihydroflavonol-4-reductase |
| **Nucleic acid-related** | | | | |  |  |  |  |
| T1LC77 | | | Pennisetum americanum | | 14.02 | 2 | 1.76E+07 | Ribosomal protein L14 |
| M7YXL2 | | | Aegilops tauschii | | 23.79 | 2 | 6.12E+07 | 50S ribosomal protein L11 |
| A0A1D6CBM9 | | | Triticum aestivum | | 24.08 | 7 | 5.84E+07 | 60S ribosomal protein L13 |
| M7ZRR9 | | | Triticum urartu | | 12.70 | 4 | 4.99E+07 | 60S ribosomal protein L35a-3 |
| W5AQP5 | | | Triticum aestivum | | 18.38 | 2 | 1.01E+07 | Transcription factor bHLH113 |
| W5BJX9 | | | Triticum aestivum | | 45.56 | 8 | 3.05E+07 | translation initiation factor 3 subunit |
| W5C5K1 | | | Triticum aestivum | | 45.91 | 11 | 2.37E+08 | translation initiation factor 3 subunit M |
| A0A1D5Y5R8 | | | Triticum aestivum | | 16.11 | 4 | 7.78E+07 | Glycine-rich RNA-binding protein |
| W5FBD5 | | | Triticum aestivum | | 17.26 | 5 | 3.63E+07 | RNA binding |
| A0A1D6B1C3 | | | Triticum aestivum | | 31.84 | 7 | 7.54E+07 | Eukaryotic translation initiation factor 3 subunit |
| M8BYQ5 | | | Aegilops tauschii | | 44.20 | 3 | 1.89E+07 | DNA repair protein RAD23 |
| A0A1D6SA10 | | | Triticum aestivum | | 28.57 | 3 | 5.24E+06 | Eukaryotic translation initiation factor 3 subunit J |
| N1R214 | | | Aegilops tauschii | | 29.77 | 2 | 6.73E+07 | RNA-binding protein 8A |
| M8D1J9 | | | Aegilops tauschii | | 31.47 | 6 | 1.02E+07 | translation initiation factor 3 subunit G |
| A0A0D3FEJ1 | | | Oryza barthii | | 101.1 | 10 | 2.42E+07 | translation elongation factor |
| I1HNH4 | | | Brachypodium distachyon | | 51.57 | 5 | 1.30E+07 | ATP-dependent RNA helicase |
| A0A1D6C2M0 | | | Triticum aestivum | | 34.07 | 3 | 6.41E+06 | ribonuclease III activity |
| A0A1D6BPS7 | | | Triticum aestivum | | 22.72 | 4 | 2.24E+07 | 7S RNA binding |
| M8B1Y7 | | | Triticum aestivum | | 38.45 | 3 | 3.23E+07 | aminoacyl-tRNA editing |
| A0A1D5VUB8 | | | Triticum aestivum | | 52.18 | 2 | 4.65E+07 | ATP binding |
| A0A1D5WJE0 | | | Triticum aestivum | | 37.15 | 4 | 5.36E+07 | exonuclease |
| A0A1D5WJJ5 | | | Triticum aestivum | | 17.20 | 4 | 5.43E+07 | RNA binding |
| **Protein synthesis/Assembly/Degradation** | | | | | | |  |  |
| A0A1D5WSP2 | | | | Triticum aestivum | 24.90 | 5 | 1.49E+08 | Multiprotein-bridging factor 1a |
| M7ZJ17 | | | | Triticum urartu | 51.74 | 5 | 2.38E+07 | 26S proteasome non-ATPase regulatory subunit 12 |
| M8AIK8 | | | | Triticum urartu | 77.35 | 9 | 3.23E+07 | glutamyl-tRNA synthetase |
| N1QYH1 | | | | Aegilops tauschii | 47.65 | 5 | 4.43E+07 | Tryptophanyl-tRNA synthetase, |
| I1HED3 | | | | Brachypodium distachyon | 81.08 | 7 | 2.88E+07 | glutamate-tRNA ligase |
| A0A1D6BLI1 | | | | Triticum aestivum | 20.72 | 3 | 8.25E+07 | Multiprotein bridging factor 1 |
| A0A1D6CXF2 | | | | Triticum aestivum | 27.34 | 3 | 2.84E+07 | Proteasome subunit alpha type |
| K7VKH1 | | | | Zea mays | 49.85 | 8 | 2.69E+07 | Tubulin alpha chain |
| A0A1D5SUI0 | | | | Aegilops tauschii | 45.53 | 4 | 2.11E+07 | MC3D protein |
| A0A1D5WX38 | | | | Triticum aestivum | 39.62 | 4 | 1.71E+08 | protein dimerization |
| F2DVH8 | | | | Hordeum vulgare | 23.28 | 2 | 4.72E+07 | Peptidylprolyl isomerase |
| A0A1D5RSE1 | | | | Triticum aestivum | 54.49 | 14 | 4.80E+06 | Aspartic proteinase |
| **Miscellaneous** | | | |  |  |  |  |  |
| A0A1D5VCD7 | | | | Triticum aestivum | 28.48 | 2 | 2.06E+07 | Very-long-chain (3R)-3-hydroxyacyl-CoA dehydratase |
| A0A1D5SR31 | | | | Triticum aestivum | 54.71 | 12 | 1.47E+07 | Phosphotransferase |
| A0A1D5STJ6 | | | | Triticum aestivum | 51.81 | 8 | 7.36E+06 | Tocopherol cyclase |
| M8BTP6 | | | | Aegilops tauschii | 50.92 | 2 | 1.29E+07 | Cell division protein ftsZ-like protein |
| A0A1D5SU38 | | | | Triticum aestivum | 41.03 | 4 | 3.38E+06 | flavin adenine dinucleotide |
| A0A1D5TRL8 | | | | Triticum aestivum | 37.78 | 6 | 1.76E+07 | isopentenyl-diphosphate delta-isomerase |
| A0A096URX8 | | | | Triticum aestivum | 40.28 | 4 | 7.14E+06 | tyrosine kinase |
| Q0J8J9 | | | | Oryza sativa | 20.47 | 2 | 1.56E+07 | Protein mago nashi homolog 1 |
| M7ZPZ7 | | | Triticum aestivum | | 44.31 | 3 | 3.87E+07 | choline-phosphate cytidylyltransferase |
| A0A1D5RQM3 | | | Triticum aestivum | | 35.93 | 3 | 1.63E+07 | 2-aminoethanethiol dioxygenase |
| A0A1D5ULL6 | | | Triticum aestivum | | 23.60 | 5 | 3.11E+07 | hydro-lyase |
| A0A1D6SBV0 | | | Triticum aestivum | | 60.98 | 6 | 5.97E+07 | T-complex protein 1 subunit gamma |
| A0A1D6PDR8 | | | Zea mays | | 91.69 | 10 | 2.54E+07 | Protein TOC75-3 chloroplastic |
| A0A1D5ZED1 | | | Triticum aestivum | | 42.44 | 6 | 2.91E+07 | transferring acyl |
| A0A1D5X8U0 | | | Triticum aestivum | | 55.79 | 5 | 1.43E+07 | Ent-kaurene acid oxidase |
| A0A1D5XGW0 | | | Triticum aestivum | | 35.22 | 2 | 8.62E+06 | Deoxymugineic acid synthase 1-B |
| **Unknown** | | |  | |  |  |  |  |
| A0A0E0DRY3 | | | Oryza meridionalis | | 17.79 | 6 | 2.36E+07 |  |
| M7ZQ23 | | | Triticum aestivum | | 44.04 | 6 | 3.50E+07 |  |
| A0A1D5S6V1 | | | Triticum aestivum | | 14.88 | 3 | 1.53E+08 |  |
| A0A1D5SCB3 | | | Triticum aestivum | | 19.93 | 3 | 1.53E+07 |  |
| A0A1D5SPJ6 | | | Triticum aestivum | | 48.05 | 6 | 6.58E+07 |  |
| A0A1D5U5Z0 | | | Triticum aestivum | | 73.30 | 11 | 3.03E+07 |  |
| N1QQW6 | | | Triticum aestivum | | 74.52 | 10 | 1.94E+07 |  |
| A0A1D5WIN7 | | | Triticum aestivum | | 34.84 | 7 | 1.53E+08 |  |
| A0A1D5XVQ8 | | | Triticum aestivum | | 23.29 | 3 | 2.92E+07 |  |
| A0A1D6CVG3 | | | Triticum aestivum | | 42.09 | 4 | 7.62E+07 |  |
| A0A1D5ZXK4 | | | Triticum aestivum | | 25.29 | 3 | 2.90E+07 |  |
| W5FIN1 | | | Triticum aestivum | | 25.91 | 3 | 2.75E+07 |  |
| W5F969 | | | Triticum aestivum | | 29.42 | 4 | 2.79E+07 |  |
| A0A1D6B6J0 | | | Triticum aestivum | | 19.10 | 2 | 2.02E+08 |  |
| A0A1D6C1Y5 | | | Triticum aestivum | | 21.42 | 5 | 3.68E+07 |  |
| A0A1D6CA80 | | | Triticum aestivum | | 19.84 | 3 | 1.92E+07 |  |
| W5ELV8 | | | Triticum aestivum | | 24.47 | 4 | 2.03E+07 |  |
| A0A1D6S9R6 | | | Triticum aestivum | | 39.54 | 5 | 3.17E+07 |  |
| A0A1D6BQH6 | | | Triticum aestivum | | 47.35 | 7 | 5.88E+07 |  |
| M7ZUF9 | | | Triticum aestivum | | 22.04 | 2 | 2.78E+07 |  |
| **II** | | |  | |  |  |  |  |
| **Accession No.** | | | **Species** | | **Mr** | **NP** | **Ratio** | **Description** |
| **N Metabolism** | | | | |  |  |  |  |
| G5CU79 | | | Elymus elongatus | | 40.16 | 3 | 1.07E+08 | Cysteine endopeptidase EP8 |
| **Carbohydrate metabolism** | | | | |  |  |  |  |
| A0A1C8E331 | | | Taeniatherum | | 66.17 | 27 | 4.69E+08 | Starch synthase, |
| M7Z133 | | | Triticum urartu | | 25.75 | 3 | 7.87E+07 | NHP2-like protein 1 |
| A0A1D5ZGJ0 | | | Triticum aestivum | | 44.01 | 8 | 3.85E+07 | Alpha-galactosidase |
| O81591 | | | Glyceria grandis | | 27.71 | 3 | 1.28E+07 | Granule-bound starch synthase |
| Q9AWE1 | | | Triticum aestivum | | 27.87 | 11 | 2.97E+07 | Granule-bound starch synthase |
| Q9SBD2 | | | Zea mays | | 27.67 | 6 | 5.10E+07 | Granule-bound starch synthase |
| D7F1C0 | | | Chasmanthium latifolium | | 27.53 | 3 | 3.55E+08 | Granule-bound starch synthase |
| Q6L798 | | | Hordeum bulbosum | | 66.48 | 24 | 6.46E+08 | Starch synthase |
| F8KGY8 | | | Calamagrostis arundinacea | | 22.73 | 8 | 4.07E+07 | Ribulose bisphosphate carboxylase large chain |
| **Energetics-related** | | | | |  |  |  |  |
| M0YLB9 | | Hordeum vulgare | | | 39.82 | 7 | 6.63E+07 | Ferredoxin--NADP reductase |
| I1HWC7 | Brachypodium distachyon | | | | 73.36 | 22 | 4.52E+07 | ATP binding |
| A0A1D5SDI4 | | | Triticum aestivum | | 61.06 | 22 | 7.41E+07 | ATP binding |
| **Stress/Defense** | | | | |  |  |  |  |
| A0A1D5X0A7 | | | | Triticum aestivum | 33.41 | 10 | 1.76E+07 | Peroxidase |
| W5D003 | | | Triticum aestivum | | 16.48 | 9 | 2.89E+08 | serine-type endopeptidase inhibitor |
| **Nucleic acid-related** | | | | |  |  |  |  |
| A0A1D6BBU1 | | | | Triticum aestivum | 86.71 | 8 | 1.23E+08 | Pre-mRNA-processing factor 39 |
| Q67W00 | | | | Oryza sativa | 36.17 | 3 | 2.04E+07 | RNA binding |
| **Unknown** | | | |  |  |  |  |  |
| A0A1D5YNZ7 | | | | Triticum aestivum | 12.19 | 3 | 4.56E+06 |  |
| A0A1D6BL73 | | | | Triticum aestivum | 64.75 | 10 | 3.17E+07 |  |

^a^ Accession number of the predicted protein in Uniprot.

^b^ Mr: Molecular mass of predicted protein.

^c^ NP: Number of matched peptides.
