# Supplementary material for: Quantitative analysis of the grain amyloplast proteome reveals differences in metabolism between two wheat cultivars at two stages of grain development
Source: BMC Genomics. 2018 Oct 24;19:768. doi: 10.1186/s12864-018-5174-z (PMC6201562; doi:10.1186/s12864-018-5174-z)
Supplement: Supplementary file 4 — Table S4. Cultivar-specific expressed proteins identified in wheat grain amyloplasts of cultivars ZM366 (I) and YM49–198 (II) at 15 DAA. (DOCX 33 kb) [file 12864_2018_5174_MOESM4_ESM.docx]

Table S4. Cultivar-specific expression proteins identified in wheat grain amyloplasts of cultivars ZM366 (I) and YM49-198 (II) at 15 DAA.

| **I** |  |  | | |  |  |  |
| --- | --- | --- | --- | --- | --- | --- | --- |
| **Accession No.** | **Species** | **NP** | | | **Mr.** | **Intensity** | **Description** |
| **N Metabolism** | |  | | |  |  |  |
| A0A1D5TBU1 | Triticum aestivum | 13 | | | 53.23 | 2.87E+07 | Adenosylhomocysteinase |
| M8CI80 | Triticum aestivum | 3 | | | 27.53 | 5.99E+06 | Proteasome subunit beta type |
| A0A1D5Z0H9 | Triticum aestivum | 4 | | | 27.87 | 1.75E+07 | Proteasome subunit beta type |
| A0A1D6A475 | Triticum aestivum | 4 | | | 52.69 | 8.23E+06 | Cysteine synthase |
| F2DBX0 | Triticum aestivum | 9 | | | 69.36 | 4.91E+07 | Acetolactate synthase |
| A0A1D6B557 | Triticum aestivum | 13 | | | 65.81 | 3.31E+07 | Aminopeptidase |
| A0A1D5VM70 | Triticum aestivum | 6 | | | 51.94 | 2.36E+07 | Carboxypeptidase |
| **Carbohydrate Metabolism** | |  | | |  |  |  |
| A0A1D6BWH0 | Triticum aestivum | 8 | | | 70.25 | 9.91E+06 | Dihydroxy-acid dehydratase |
| A0A1D6D7L9 | Triticum aestivum | 5 | | | 87.17 | 7.48E+07 | Dolichyl-diphosphooligosaccharide--protein glycosyltransferase subunit STT3 |
| B6T3P9 | Zea mays | 14 | | | 48.13 | 3.80E+07 | Enolase |
| A0A1D5SY22 | Triticum aestivum | 5 | | | 76.16 | 4.59E+07 | Pyruvate kinase |
| E0WBH7 | Elymus burchan | 10 | | | 27.77 | 4.72E+06 | POAL Granule bound starch synthase |
| A0A1D5X0P4 | Triticum aestivum | 9 | | | 83.65 | 5.46E+07 | Hydrolyzing O-glycosyl c |
| A0A1D6BVA5 | Triticum aestivum | 12 | | | 100.0 | 7.09E+07 | Cellulose synthase (UDP-forming) |
| W5GF36 | Triticum aestivum | 12 | | | 78.61 | 3.63E+06 | 3-hydroxyacyl-CoA dehydrogenas |
| A0A1D6B2M0 | Triticum aestivum | 11 | | | 45.18 | 2.36E+08 | Succinate-CoA ligase subunit beta |
| M7ZAC1 | Triticum urartu | 17 | | | 50.93 | 1.13E+07 | Ribulose bisphosphate carboxylase/oxygenase activase A, chloroplastic |
| A0A096UJV2 | Triticum aestivum | 5 | | | 28.14 | 4.32E+07 | Chlorophyll a-b binding protein, chloroplastic |
| M8A2T1 | Triticum urartu | 13 | | | 46.56 | 1.46E+07 | Ribulose bisphosphate carboxylase large chain |
| **Energetics-related** | |  | | |  |  |  |
| A0A0G2QKG4 | Triticum aestivum | 3 | | | 29.17 | 4.44E+07 | Vacuolar ATPase subunit D1 |
| Q9G725 | Elymus sibiricus | 5 | | | 10.71 | 4.89E+07 | L F-1-ATPase alpha subunit |
| A0A1D5XUI4 | Triticum aestivum | 20 | | | 80.13 | 4.05E+07 | NADH dehydrogenase (ubiquinone) |
| A0A1D6BXL7 | Triticum aestivum | 8 | | | 39.84 | 3.63E+07 | Ferredoxin--NADP reductase |
| B2B9U2 | Triticum aestivum | 3 | | | 12.39 | 1.29E+07 | ATPase subunit G |
| A0A1D5SG34 | Triticum aestivum | 16 | | | 61.61 | 8.07E+07 | ATP binding |
| A0A1D5UE96 | Triticum aestivum | 5 | | | 51.38 | 2.74E+07 | Proton-transporting ATPase |
| **Transport** | |  | | |  |  |  |
| M7ZW32 | Triticum urartu | 4 | | | 53.09 | 1.24E+07 | AP-4 complex subunit mu-1 |
| M8ATP1 | Triticum aestivum | 6 | | | 74.51 | 4.12E+07 | Transmembrane 9 superfamily member |
| M0XYC3 | Triticum urartu | 7 | | | 89.37 | 3.01E+07 | Multiple C2 and transmembrane domain-containing protein 1 |
| N1R259 | Triticum aestivum | 3 | | | 31.53 | 2.79E+07 | Reticulon-like protein |
| A0A0P0VL75 | Oryza sativa | 3 | | | 26.00 | 4.36E+07 | Vacuolar transport |
| J3MFF9 | Oryza brachyantha | 3 | | | 42.20 | 5.65E+07 | Transmembrane transport |
| A0A1D5Z640 | Triticum aestivum | 2 | | | 11.15 | 2.11E+07 | Protein transport protein Sec61 |
| **Signal Transduction** | |  | | |  |  |  |
| A0A1D6B740 | Triticum aestivum | 12 | | | 99.06 | 8.19E+06 | GTPase |
| **Stress/Defense** | |  | | |  |  |  |
| H2DPU3 | Triticum aestivum | 5 | | | 61.77 | 3.10E+07 | Polyphenol oxidase |
| A0A1D5UXT7 | Triticum aestivum | 3 | | | 13.08 | 2.39E+07 | Cysteine proteinase inhibitor |
| A0A1D5YIT2 | Triticum aestivum | 3 | | | 37.83 | 7.27E+07 | Lactoylglutathione lyase |
| M8BMF3 | Triticum aestivum | 2 | | | 36.31 | 7.49E+06 | Autophagy-related protein 3 |
| M7YQT7 | Triticum urartu | 4 | | | 36.30 | 3.45E+06 | Dihydroflavonol-4-reductase |
| W5AQ87 | Triticum aestivum | 3 | | | 13.29 | 2.14E+07 | Glutaredoxin |
| A0A1D6CI91 | Triticum aestivum | 2 | | | 8.11 | 3.31E+07 | Defensin Tk-AMP-D4 |
| M8CFS7 | Aegilops tauschii | 5 | | | 45.87 | 1.05E+08 | Peroxiredoxin Q, |
| **Nucleic acid-related** | |  | | |  |  |  |
| A0A1D5V7I2 | Triticum aestivum | 7 | | | 30.36 | 2.42E+07 | RNA binding |
| A0A1D5UZ25 | Triticum aestivum | 11 | | | 63.60 | 4.21E+07 | pseudouridine synthase |
| M8BTU8 | Triticum aestivum | 6 | | | 42.74 | 3.80E+07 | Translation initiation factor 2 subunit alpha |
| M8A374 | Triticum urartu | 4 | | | 11.43 | 6.00E+07 | Histone H4 |
| M8AER1 | Triticum urartu | 4 | | | 45.96 | 2.08E+07 | Eukaryotic translation initiation factor 2 subunit beta |
| A0A1D6D9Y7 | Triticum aestivum | 4 | | | 15.30 | 3.47E+07 | Histone H3 |
| **Protein synthesis/Assembly/Degradation** | | | | | |  |  |
| A0A1D6LN76 | Zea mays | | 2 | | 30.50 | 8.39E+06 | Peptidyl-prolyl cis-trans isomerase |
| M7ZDC1 | Triticum urartu | | 5 | | 60.20 | 3.67E+07 | Peptide chain release factor GTP-binding subunit ERF3B |
| M8A4L9 | Triticum urartu | | 5 | | 48.38 | 2.78E+07 | Sorting nexin-1 |
| A0A0E0DRY3 | Oryza meridionalis | | 6 | | 17.79 | 6.86E+06 | Protein heterodimerization |
| A0A1D5VZH2 | Triticum aestivum | | 17 | | 91.13 | 7.17E+06 | Glutamine-tRNA ligase |
| A0A1D6BKU8 | Triticum aestivum | | 17 | | 72.68 | 1.27E+07 | Metalloendopeptidase |
| **Miscellaneous** | | |  | |  |  |  |
| Q06I75 | Triticum aestivum | | 4 | | 30.34 | 4.85E+07 | Fasciclin-like protein |
| F2CQL4 | Triticum aestivum | | 2 | | 13.51 | 9.95E+07 | Peptidase |
| M8BQN3 | Triticum aestivum | | 8 | | 69.29 | 1.38E+07 | Vacuolar-sorting receptor 1 |
| CON__P02538 | Triticum aestivum | | 12 | | 60.04 | 4.70E+07 | Keratin, type II cytoskeletal 6A |
| A0A1D6SCC4 | Triticum aestivum | | 6 | | 48.40 | 2.97E+07 | 3-oxoacyl-[acyl-carrier-protein] synthase |
| B6DU63 | Triticum aestivum | | 5 | | 62.54 | 4.64E+07 | Zeta-carotene desaturase |
| N1QPC6 | Aegilops tauschii | | 7 | | 75.09 | 1.56E+07 | Fatty acyl-CoA synthetase B |
| M8CFD2 | Aegilops tauschii | | 5 | | 78.40 | 9.71E+06 | Long-chain-fatty-acid--CoA ligase 4 |
| A0A1D5XDV1 | Triticum aestivum | | 13 | | 50.29 | 2.27E+07 | Transaminase |
| A0A1D6C5Q0 | Triticum aestivum | | 5 | | 56.59 | 4.16E+08 | Purple acid phosphatase |
| Q0GJJ2 | Triticum aestivum | | 3 | | 38.54 | 1.52E+07 | beta-hydroxysteroid dehydrogenase-like protein |
| M7ZF90 | Triticum urartu | | 4 | | 59.86 | 3.29E+07 | Complex protein 1 subunit epsilon |
| **Unknown** | | |  | |  |  |  |
| A0A1D5VE94 | Triticum aestivum | | 3 | | 29.88 | 7.33E+07 |  |
| Q5NKR1 | Triticum monococcum | | 4 | | 26.16 | 1.37E+07 |  |
| A0A1D5SBZ7 | Triticum aestivum | | 6 | | 29.58 | 5.02E+07 |  |
| A0A1D5T8I8 | Triticum aestivum | | 5 | | 10.71 | 6.01E+07 |  |
| A0A1D5UDC2 | Triticum aestivum | | 5 | | 34.08 | 6.48E+07 |  |
| F2D4H7 | Triticum aestivum | | 4 | | 33.45 | 4.80E+07 |  |
| A0A1D5XG60 | Triticum aestivum | | 11 | | 81.13 | 1.20E+08 |  |
| M0XGG6 | Triticum aestivum | | 10 | | 57.52 | 2.78E+07 |  |
| A0A1D5ZUV0 | Triticum aestivum | | 5 | | 50.02 | 2.04E+07 |  |
| R7WDB3 | Triticum aestivum | | 5 | | 23.84 | 5.40E+07 |  |
| A0A1D6CZK2 | Triticum aestivum | | 3 | | 9.68 | 1.72E+07 |  |
| M8C7G1 | Triticum aestivum | | 7 | | 37.17 | 3.32E+07 |  |
| W5BBP4 | Triticum aestivum | | 7 | | 41.37 | 2.72E+07 |  |
| I1HRV9 | Triticum aestivum | | 2 | | 15.09 | 3.91E+07 |  |
| A0A1D6S578 | Triticum aestivum | | 5 | | 15.60 | 5.50E+07 |  |
| **II** |  | |  | |  |  |  |
| **Accession No.** | **Species** | | **NP** | | **Mr.** | **Intensity** | **Description** |
| **Carbohydrate Metabolism** | | |  | |  |  |  |
| A0A0D9VKT9 | Oryza brachyantha | | 5 | | 42.71 | 5.07E+07 | Pyruvate dehydrogenase E1 component subunit alpha |
| A9UGN1 | Hordeum marinum | | 2 | | 25.13 | 1.81E+07 | Beta-amylase |
| M8D1A8 | Aegilops tauschii | | 12 | | 39.54 | 4.72E+07 | Formate dehydrogenase |
| A0A1D6L3I4 | Zea mays | | 6 | | 14.49 | 8.59E+07 | Granule-bound starch synthase 1 |
| M7ZSW8 | Triticum urartu | | 7 | | 56.08 | 3.81E+06 | Photosystem II CP47 reaction center protein |
| **Signal Transduction** | | |  | |  |  |  |
| B4FKM1 | Zea mays | | 5 | | 36.24 | 4.12E+07 | Guanine nucleotide-binding protein beta subunit-like protein |
| A0A1D5Y502 | Triticum aestivum | | 4 | | 16.23 | 2.78E+08 | Histidine-containing phosphotransfer protein 1 |
| A0A0D9WHD0 | Oryza punctata | | 4 | | 36.30 | 2.33E+06 | GTPase |
| **Stress/Defense** |  | |  | |  |  |  |
| E0WC53 | Triticum aestivum | | 3 | | 28.07 | 1.41E+07 | WALI7 |
| A0A1D5X0A7 | Triticum aestivum | | 10 | | 33.41 | 1.65E+07 | Peroxidase |
| Q9S6Y2 | Triticum aestivum | | 4 | | 9.53 | 5.62E+08 | Alpha purothionin |
| A0A1D6BF22 | Triticum aestivum | | 4 | | 56.20 | 3.93E+07 | Peroxidase |
| W5D003 | Triticum aestivum | | 9 | | 16.48 | 1.05E+09 | Dimeric alpha-amylase inhibitor |
| **Nucleic acid-related** | | |  | |  |  |  |
| A0A0D9VHT5 | Oryza meridionalis | | 7 | | 51.08 | 3.25E+07 | Elongation factor Tu |
| A0A1D5UBI3 | Triticum aestivum | | 8 | | 23.32 | 4.52E+07 | Elongation factor 1-beta |
| A0A1D6D2K1 | Triticum aestivum | | 8 | | 42.94 | 5.59E+06 | Chromatin binding |
| A0A1D6C024 | Triticum aestivum | | 5 | | 27.98 | 3.41E+07 | RNA binding |
| A0A1E5UVD1 | Oryza rufipogon | | 3 | | 27.57 | 9.74E+07 | 40S ribosomal protein S8 |
| **Protein synthesis/Assembly/Degradation** | | | | | | |  |
| A0A1D6B1K1 | Triticum aestivum | | | 5 | 31.26 | 1.72E+07 | Proteasome subunit alpha type |
| **Miscellaneous** |  | | |  |  |  |  |
| W5E0L9 | Triticum aestivum | | | 4 | 39.85 | 1.16E+08 | [acyl-carrier-protein] S-malonyltransferase |
| **Unknown** |  | | |  |  |  |  |
| A0A1D5SAF6 | Triticum aestivum | | | 4 | 28.05 | 1.30E+07 |  |
| A0A1D5T395 | Triticum aestivum | | | 6 | 26.91 | 7.37E+07 |  |

^a^ Accession number of the predicted protein in Uniprot.

^b^ Mr: Molecular mass of predicted protein.

^c^ NP: Number of matched peptides.
