# Supplementary material for: Quantitative analysis of the grain amyloplast proteome reveals differences in metabolism between two wheat cultivars at two stages of grain development
Source: BMC Genomics. 2018 Oct 24;19:768. doi: 10.1186/s12864-018-5174-z (PMC6201562; doi:10.1186/s12864-018-5174-z)
Supplement: Supplementary file 5 — Table S5. Differentially expressed proteins identified between ZM366 and YM49–198 at 10 DAA (I) and 15 DAA (II). (DOCX 50 kb) [file 12864_2018_5174_MOESM5_ESM.docx]

Table S5. Differentially expressed proteins identified between ZM366 and YM49-198 at 10 DAA (I) and 15 DAA (II).

| **I** | |  |  |  |  |  |
| --- | --- | --- | --- | --- | --- | --- |
| **Accession No.** | | **Species** | **Mr.** | **Ratio** | ***p* value** | **Description** |
| **N Metabolism** | | |  |  |  |  |
| A0A077S2R7 | Triticum aestivum | | 111.04 | 2.079 | 0.045 | Glycine cleavage system P protein |
| M9T6Y7 | Triticum turgidum | | 69.98 | 0.343 | 0.030 | Sulfite reductase |
| S5YTU4 | Triticum aestivum | | 235.1 | 0.255 | 0.031 | NADH-dependent glutamate synthase |
| A0A1D5X2D4 | Hordeum vulgare | | 29.88 | 0.445 | 0.029 | nitrite reductase [NAD(P)H] |
| A0A1D6C6R0 | Hordeum vulgare | | 41.14 | 0.403 | 0.020 | indole-3-glycerol-phosphate synthase |
| **Carbohydrate Metabolism** | | |  |  |  |  |
| W5F620 | Triticum aestivum | | 44.00 | 2.160 | 0.031 | Alpha-galactosidase |
| A0A1B1V4Q3 | Triticum aestivum | | 36.55 | 2.322 | 0.034 | Glyceraldehyde-3-phosphate dehydrogenase |
| A0A1D5XGF3 | Triticum aestivum | | 58.73 | 2.984 | 0.049 | Beta-amylase |
| A0A1D5WP73 | Triticum aestivum | | 54.44 | 3.937 | 0.007 | Pectin acetylesterase |
| A0A1D6D1Q3 | Triticum aestivum | | 60.56 | 0.331 | 0.033 | Pyrophosphate--fructose 6-phosphate 1-phosphotransferase subunit beta |
| A0A1D5YUK3 | Triticum aestivum | | 67.60 | 0.343 | 0.004 | Pyrophosphate--fructose 6-phosphate 1-phosphotransferase subunit alpha |
| A0A1D5TM13 | Triticum aestivum | | 45.89 | 0.371 | 0.007 | Pyruvate dehydrogenase E1 component subunit alpha |
| I1HTH7 | Triticum aestivum | | 55.51 | 0.476 | 0.019 | Glycerol-3-phosphate acyltransferase 6 |
| A0A1D5SCW4 | Triticum aestivum | | 53.92 | 0.463 | 0.050 | 6-phosphogluconate dehydrogenase, decarboxylating |
| A0A1D6D7L9 | Triticum aestivum | | 87.17 | 0.320 | 0.010 | Dolichyl-diphosphooligosaccharide--protein glycosyltransferase subunit STT3 |
| M8AYR9 | Aegilops tauschii | | 70.51 | 0.280 | 0.035 | Dolichyl-diphosphooligosaccharide--protein glycosyltransferase subunit 1 |
| A0A1D6RJE7 | Triticum aestivum | | 52.52 | 0.475 | 0.013 | Dolichyl-diphosphooligosaccharide--protein glycosyltransferase subunit 1 |
| A0A1D5T6Q3 | Triticum aestivum | | 72.40 | 0.496 | 0.016 | Starch synthase |
| A0A1D5ZV85 | Triticum aestivum | | 48.66 | 0.373 | 0.031 | Dihydrolipoamide acetyltransferase component of pyruvate dehydrogenase complex |
| A0A1D5S0J4 | Triticum aestivum | | 53.34 | 0.389 | 0.038 | Endoglucanase |
| A0A1D5WKF4 | Triticum aestivum | | 63.65 | 0.430 | 0.049 | phosphoglycerate mutase |
| A0A1D5WLK3 | Hordeum vulgare | | 36.02 | 0.161 | 0.025 | hydrolyzing O-glycosyl compounds |
| A0A024BLG7 | Pennisetum americanum | | 8.90 | 0.375 | 0.002 | Photosystem I iron-sulfur center |
| A0A1D5SCA9 | Triticum aestivum | | 45.97 | 0.495 | 0.002 | NADPH-protochlorophyllide oxidoreductase B |
| **Energetics-related** | | |  |  |  |  |
| A0A1D6BXL7 | Triticum aestivum | | 39.84 | 0.325 | 0.001 | Ferredoxin--NADP reductase |
| A0A1D5ZAK3 | Triticum aestivum | | 30.65 | 0.341 | 0.014 | Ferritin |
| A0A1D5VGQ8 | Triticum aestivum | | 33.84 | 0.273 | 0.008 | NADH-cytochrome b5 reductase |
| M8CW07 | Aegilops tauschii | | 108.5 | 0.367 | 0.015 | Putative ATP-citrate synthase |
| F2CUJ6 | Hordeum vulgare | | 68.14 | 0.438 | 0.028 | ATPase activity |
| W5DVY1 | Triticum aestivum | | 70.91 | 0.245 | 0.001 | ATP binding |
| **Transport** | | |  |  |  |  |
| A0A077KS83 | Triticum aestivum | | 25.66 | 3.124 | 0.048 | Aquaporin |
| A0A1D5VNA4 | Triticum aestivum | | 72.82 | 0.339 | 0.028 | ADP, ATP carrier protein |
| W5AA91 | Triticum aestivum | | 58.62 | 0.336 | 0.003 | Importin subunit alpha |
| M8AQF7 | Triticum aestivum | | 195.85 | 0.477 | 0.012 | Clathrin heavy chain |
| A0A1D5SUU2 | Triticum aestivum | | 56.64 | 0.073 | 0.009 | Coatomer subunit delta-1 |
| W5D5P8 | Triticum aestivum | | 105.39 | 0.483 | 0.007 | Coatomer subunit beta |
| A0A1D5XKY8 | Triticum aestivum | | 135.82 | 0.301 | 0.008 | Coatomer subunit alpha |
| **Signal Transduction** | | |  |  |  |  |
| A0A1D5Y502 | Triticum aestivum | | 16.23 | 2.569 | 0.007 | Histidine-containing phosphotransfer protein 1 |
| A0A1D5UBN2 | Triticum aestivum | | 27.27 | 2.266 | 0.048 | UMP-CMP kinase |
| A0A1D5WBE0 | Triticum aestivum | | 31.93 | 3.548 | 0.011 | 14-3-3 protein |
| P29305 | Hordeum vulgare | | 29.35 | 0.318 | 0.045 | 14-3-3-like protein A |
| M7ZQ50 | Triticum urartu | | 28.51 | 0.149 | 0.048 | GTP-binding protein SAR1A |
| A0A1D5SVG3 | Triticum aestivum | | 151.78 | 0.242 | 0.005 | GTPase |
| A0A1D5SB20 | Triticum aestivum | | 82.70 | 0.466 | 0.020 | GTPase |
| A0A077S025 | Triticum aestivum | | 90.21 | 0.351 | 0.022 | GTPase |
| A0A1D6CFI9 | Triticum aestivum | | 98.60 | 0.233 | 0.008 | GTPase |
| **Stress/Defense** | | |  |  |  |  |
| A0A1D6ACI6 | Triticum aestivum | | 12.63 | 5.474 | 0.000 | serine-type endopeptidase inhibitor |
| A0A1D5XMK2 | Triticum aestivum | | 14.73 | 3.393 | 0.006 | serine-type endopeptidase inhibitor |
| M8A1S2 | Triticum urartu | | 16.47 | 3.320 | 0.013 | Trypsin/alpha-amylase inhibitor CMX1/CMX3 |
| N1QTW5 | Triticum aestivum | | 15.26 | 2.432 | 0.015 | Trypsin inhibitor CMc |
| P17314 | Triticum aestivum | | 18.22 | 4.674 | 0.018 | Alpha-amylase/trypsin inhibitor CM3 |
| M8BV45 | Aegilops tauschii | | 24.25 | 5.966 | 0.000 | Alpha-amylase/trypsin inhibitor CM3 |
| Q7M219 | Triticum turgidum | | 2.39 | 4.148 | 0.003 | Alpha-amylase inhibitor (Fragment) |
| Q84U03 | Triticum aestivum | | 38.82 | 3.987 | 0.008 | Peroxidase |
| A0A1D6BF22 | Triticum aestivum | | 56.20 | 4.347 | 0.016 | Peroxidase |
| A0A1D5V365 | Aegilops tauschii | | 27.66 | 0.456 | 0.037 | L-ascorbate peroxidase 2 |
| C0LF30 | Triticum aestivum | | 43.12 | 7.145 | 0.003 | Serpin 1 |
| S4VQP9 | Triticum aestivum | | 58.05 | 0.347 | 0.014 | Chaperonin family theta subunit |
| N1R361 | Aegilops tauschii | | 160.05 | 0.423 | 0.018 | Heat shock cognate 70 kDa protein |
| A0A1D5YB80 | Aegilops tauschii | | 72.75 | 0.357 | 0.001 | Heat shock 70 kDa protein |
| M8AVT1 | Triticum urartu | | 76.90 | 0.239 | 0.044 | Heat shock cognate 70 kDa protein |
| F4Y5B2 | Aegilops tauschii | | 80.46 | 0.396 | 0.044 | Heat shock protein 90 |
| M7YVS8 | Triticum aestivum | | 15.28 | 0.283 | 0.003 | Profilin |
| **Nucleic acid-related** | | |  |  |  |  |
| A0A1D5ZLD1 | Triticum aestivum | | 40.66 | 2.503 | 0.007 | 5S rRNA binding |
| A0A1D5TE22 | Triticum aestivum | | 47.22 | 2.859 | 0.014 | RNA binding |
| M7Z7E7 | Triticum aestivum | | 66.92 | 2.929 | 0.039 | Splicing factor U2af large subunit B |
| M8C107 | Aegilops tauschii | | 24.94 | 0.313 | 0.046 | translation initiation factor 5A-1 |
| A0A1D5ZGU3 | Aegilops tauschii | | 75.10 | 0.204 | 0.000 | Eukaryotic translation initiation factor 2 subunit 3 |
| M8CZY8 | Aegilops tauschii | | 104.85 | 0.225 | 0.019 | Eukaryotic translation initiation factor 3 subunit C |
| R7W8M7 | Aegilops tauschii | | 45.83 | 0.342 | 0.029 | Replication factor C subunit 3 |
| A0A0A7C089 | Triticum aestivum | | 18.81 | 0.367 | 0.044 | Translationally controlled protein |
| E6Y289 | Triticum aestivum | | 18.78 | 0.461 | 0.039 | Translationally-controlled protein |
| A0A1D6S5Y2 | Triticum aestivum | | 17.75 | 0.263 | 0.007 | Histone H2B |
| N1QW22 | Aegilops tauschii | | 61.83 | 0.407 | 0.028 | 26S protease regulatory subunit 6A-like protein |
| M8BBE3 | Aegilops tauschii | | 25.35 | 0.480 | 0.001 | Elongation factor 1-delta |
| A0A1E5W0T3 | Zea mays | | 49.24 | 0.352 | 0.030 | Elongation factor 1-alpha |
| A0A1D6C4E9 | Hordeum | | 46.89 | 0.410 | 0.024 | elongation factor 1 gamma |
| A0A1D5TKE5 | Triticum aestivum | | 23.39 | 0.286 | 0.005 | translation elongation factor |
| A0A1D5XUJ1 | Triticum aestivum | | 9.20 | 0.489 | 0.020 | 40S ribosomal protein S21 |
| G4XH71 | Triticum aestivum | | 18.38 | 0.273 | 0.040 | Peptidyl-prolyl cis-trans isomerase |
| N0E6R8 | Triticum aestivum | | 90.69 | 0.265 | 0.031 | DEAD-box ATP-dependent RNA helicase, putative |
| M7YKC6 | Triticum aestivum | | 95.49 | 0.046 | 0.008 | Ribonucleoside-diphosphate reductase |
| **Protein synthesis/Assembly/Degradation** | | | |  |  |  |
| A0A1D5Z1A0 | Triticum aestivum | | 60.74 | 2.597 | 0.013 | metalloendopeptidase |
| CON__P07477 | Triticum aestivum | | 26.56 | 4.827 | 0.034 | Trypsin-1 precursor |
| A0A1D6B662 | Triticum aestivum | | 87.55 | 0.329 | 0.050 | metalloendopeptidase |
| A5CFY5 | Triticum aestivum | | 50.05 | 0.171 | 0.006 | Tubulin beta-3 chain |
| P20973 | Triticum aestivum | | 117.01 | 0.151 | 0.000 | Ubiquitin-activating enzyme E1 |
| R7WCF8 | Aegilops tauschii | | 47.01 | 0.159 | 0.011 | FAM10 family protein |
| M8A7K2 | Triticum urartu | | 49.58 | 0.247 | 0.001 | DnaJ protein-like protein |
| W5EU17 | Triticum aestivum | | 49.05 | 2.102 | 0.012 | Aspartic proteinase nepenthesin-1 |
| A0A1D5VCN0 | Triticum aestivum | | 53.09 | 4.248 | 0.019 | Aspartic proteinase oryzasin-1 |
| A0A1D5T408 | Triticum aestivum | | 73.71 | 0.175 | 0.002 | arginine-tRNA ligase |
| **Miscellaneous** | | |  |  |  |  |
| N1R356 | Triticum aestivum | | 41.38 | 2.068 | 0.019 | Formate dehydrogenase |
| A0A1D5WYI6 | Triticum aestivum | | 30.49 | 2.679 | 0.011 | acid phosphatase |
| A0A1D5UG29 | Triticum aestivum | | 57.15 | 2.070 | 0.016 | methylmalonate-semialdehyde dehydrogenase |
| I1I5U0 | Triticum aestivum | | 41.01 | 0.339 | 0.023 | Methyltransferase |
| A0A1D6AF35 | Aegilops tauschii | | 39.62 | 0.456 | 0.029 | C-1-tetrahydrofolate synthase, |
| D2KZ12 | Triticum aestivum | | 47.93 | 0.499 | 0.016 | 3-ketoacyl-CoA thiolase-like protein |
| M7ZE51 | Triticum urartu | | 56.01 | 0.397 | 0.025 | Caffeoyl-CoA O-methyltransferase 2 |
| M7ZJZ8 | Triticum urartu | | 45.35 | 0.236 | 0.003 | 3-ketoacyl-CoA thiolase 2 |
| A0A1D5T4H8 | Triticum aestivum | | 53.88 | 0.253 | 0.013 | transaminase |
| A0A1D6ANS3 | Triticum aestivum | | 53.46 | 0.410 | 0.010 | geranylgeranyl reductase |
| W5FMA5 | Triticum aestivum | | 56.34 | 0.429 | 0.035 | Protoporphyrinogen oxidase |
| Q06I75 | Triticum aestivum | | 30.34 | 0.244 | 0.009 | Fasciclin-like protein FLA31 |
| W5G5V4 | Triticum aestivum | | 85.47 | 0.155 | 0.022 | S-adenosylmethionine-dependent methyltransferase |
| **Unknown** |  | |  |  |  |  |
| W5EFT2 | Triticum aestivum | | 25.15 | 3.228 | 0.002 |  |
| A0A1D5XS09 | Triticum aestivum | | 68.91 | 14.806 | 0.003 |  |
| A0A1D5SXT1 | Triticum aestivum | | 38.87 | 2.327 | 0.007 |  |
| A0A1D6C0D3 | Triticum aestivum | | 17.74 | 6.792 | 0.007 |  |
| W5EST8 | Triticum aestivum | | 70.62 | 2.880 | 0.013 |  |
| A0A1D5S0Z9 | Triticum aestivum | | 63.87 | 2.903 | 0.038 |  |
| A0A1D5X2J6 | Triticum aestivum | | 18.19 | 5.681 | 0.050 |  |
| A0A1D6C0U4 | Triticum aestivum | | 66.26 | 0.313 | 0.002 |  |
| W5AU55 | Triticum aestivum | | 45.72 | 0.193 | 0.004 |  |
| W5EB84 | Triticum aestivum | | 108.65 | 0.440 | 0.004 |  |
| A0A1D5TT49 | Triticum aestivum | | 63.40 | 0.255 | 0.030 |  |
| M8BUH9 | Triticum aestivum | | 39.36 | 0.456 | 0.032 |  |
| A0A1D5X3W3 | Triticum aestivum | | 50.70 | 0.214 | 0.045 |  |
| A0A1D5X267 | Triticum aestivum | | 18.89 | 0.123 | 0.045 |  |
| W5FLK8 | Triticum aestivum | | 24.05 | 0.151 | 0.026 |  |
| A0A1D6CDM8 | Triticum aestivum | | 19.55 | 0.188 | 0.001 |  |
| M8BZG3 | Triticum aestivum | | 21.00 | 0.301 | 0.033 |  |
| A0A1D5YMS0 | Triticum aestivum | | 29.34 | 0.419 | 0.038 |  |
| A0A1D5VA12 | Triticum aestivum | | 51.48 | 0.311 | 0.014 |  |
| F2DVW0 | Triticum aestivum | | 28.49 | 0.473 | 0.015 |  |
| W5CVF8 | Triticum aestivum | | 17.59 | 0.083 | 0.017 |  |
| T1N701 | Triticum aestivum | | 158.51 | 0.460 | 0.014 |  |
| M8CG75 | Triticum aestivum | | 37.03 | 0.303 | 0.012 |  |
| A0A1D5SUM8 | Triticum aestivum | | 64.80 | 0.483 | 0.001 |  |
| F2DNL6 | Triticum aestivum | | 60.17 | 0.141 | 0.026 |  |
| A0A1D6AKJ1 | Triticum aestivum | | 20.60 | 0.442 | 0.015 |  |
| A0A1D6B042 | Triticum aestivum | | 23.26 | 0.348 | 0.045 |  |
| A0A1D5VE94 | Triticum aestivum | | 29.88 | 0.198 | 0.001 |  |
| **II** |  | |  |  |  |  |
| **Accession No.** | **Species** | | **Mr.** | **Ratio** | ***p* value** | **Description** |
| **N Metabolism** | | |  |  |  |  |
| S5YTU4 | Triticum turgidum | | 235.17 | 23.054 | 0.000 | NADH-dependent glutamate synthase |
| W5BES7 | Triticum aestivum | | 80.04 | 2.113 | 0.001 | protein serine/threonine kinase |
| W5B8D7 | Triticum aestivum | | 56.74 | 0.337 | 0.004 | Glutamate dehydrogenase |
| W5AC96 | Triticum aestivum | | 52.14 | 0.285 | 0.019 | Carboxypeptidase |
| **Carbohydrate Metabolism** | | |  |  |  |  |
| R7W9X7 | Aegilops tauschii | | 79.79 | 2.012 | 0.031 | Beta-fructofuranosidase, insoluble isoenzyme 4 |
| M8CW07 | Aegilops tauschii | | 108.54 | 0.342 | 0.008 | Putative ATP-citrate synthase |
| A0A1B6PEI8 | Sorghum bicolor | | 47.10 | 0.428 | 0.012 | Malate dehydrogenase |
| A0A1D5WP39 | Triticum aestivum | | 51.82 | 0.448 | 0.010 | sedoheptulose-7-phosphate:D-glyceraldehyde-3-phosphate glyceronetransferase |
| A0A1D5RY74 | Triticum aestivum | | 35.53 | 0.322 | 0.011 | Malate dehydrogenase |
| A0A1D5W5J6 | Triticum aestivum | | 38.02 | 0.159 | 0.015 | hydrolyzing O-glycosyl |
| M7ZLJ5 | Triticum urartu | | 54.68 | 0.414 | 0.022 | dehydrogenase [ubiquinone] flavoprotein 1 |
| W5AN92 | Triticum aestivum | | 62.13 | 0.304 | 0.049 | Dihydrolipoyl dehydrogenase |
| M7Z323 | Aegilops tauschi | | 40.33 | 0.198 | 0.001 | Putative succinyl-CoA ligase [ADP-forming] subunit alpha |
| N1R3V3 | Triticum aestivum | | 60.73 | 0.499 | 0.001 | Acetyltransferase component of pyruvate dehydrogenase complex |
| A0A0Q3K692 | Brachypodium distachyon | | 58.84 | 0.296 | 0.031 | Acetyltransferase component of pyruvate dehydrogenase complex |
| A0A1D5YR00 | Triticum aestivum | | 22.98 | 0.304 | 0.001 | Cytochrome b-c1 complex subunit 7 |
| A0A1D5TPW6 | Triticum aestivum | | 23.70 | 0.305 | 0.008 | Cytochrome b6-f complex iron-sulfur subunit |
| A0A1D5SV65 | Triticum aestivum | | 28.73 | 0.366 | 0.023 | Chlorophyll a-b binding protein, |
| A0A024BKF6 | Pennisetum americanum | | 38.93 | 0.204 | 0.032 | Photosystem II protein D1 |
| **Energetics-related** | | |  |  |  |  |
| K3XVX4 | Setaria italica | | 64.91 | 2.112 | 0.001 | ATP binding |
| A0A191TDI3 | Hordeum vulgare | | 55.31 | 0.351 | 0.000 | ATP synthase subunit alpha |
| A0A1D5RP45 | Triticum aestivum | | 63.53 | 0.471 | 0.001 | ATP synthase subunit beta |
| F2VQK3 | Triticum aestivum | | 21.62 | 0.395 | 0.007 | ATP4-1 |
| A0A1D5T2S6 | Triticum aestivum | | 35.44 | 0.335 | 0.008 | proton-transporting ATP synthase |
| A0A1D5TNA2 | Triticum aestivum | | 25.90 | 0.495 | 0.028 | electron transport chain |
| M8AIK4 | Triticum aestivum | | 104.98 | 0.410 | 0.028 | Plasma membrane ATPase |
| **Transport** | | |  |  |  |  |
| W5AA91 | Triticum aestivum | | 58.62 | 2.070 | 0.001 | Importin subunit alpha |
| A0A1D5VXC3 | Triticum aestivum | | 58.95 | 2.633 | 0.003 | Importin subunit alpha |
| M0XEJ4 | Hordeum vulgare | | 95.70 | 2.097 | 0.010 | Importin subunit beta-1 |
| A0A1D5ZF82 | Triticum aestivum | | 108.3 | 2.415 | 0.017 | protein transporter |
| A0A1D5Z743 | Triticum aestivum | | 24.10 | 2.051 | 0.044 | copper ion transmembrane transporter |
| M7Z5I7 | Triticum urartu | | 32.34 | 0.148 | 0.001 | uncoupling protein 3 |
| A0A1D6CJX0 | Hordeum vulgare subsp | | 41.45 | 0.348 | 0.000 | transporter |
| A0A1D5TIE5 | Triticum aestivum | | 30.37 | 0.429 | 0.001 | transporter activity |
| A0A1D6ANT1 | Triticum aestivum | | 39.42 | 0.269 | 0.010 | transport |
| I1IV99 | Brachypodium distachyon | | 10.83 | 0.358 | 0.030 | transport protein Sec61 subunit beta |
| A0A1D6AX03 | Triticum aestivum | | 45.89 | 0.493 | 0.014 | ADP-glucose brittle-1 transporter |
| M7ZDS1 | Triticum aestivum | | 32.69 | 0.353 | 0.008 | 2-oxoglutarate/malate carrier protein |
| M8A2G0 | Triticum urartu | | 41.40 | 0.405 | 0.011 | ADP,A TP carrier protein |
| M8C0V9 | Aegilops tauschii | | 85.25 | 0.363 | 0.031 | Transmembrane 9 superfamily member |
| A0A1D5ZTV6 | Triticum aestivum | | 73.31 | 0.488 | 0.007 | Transmembrane 9 superfamily member |
| W5DWT9 | Triticum aestivum | | 37.01 | 0.139 | 0.001 | transmembrane transport |
| M7ZFX1 | Triticum urartu | | 64.60 | 0.414 | 0.041 | outer membrane porin |
| **Signal Transduction** | | |  |  |  |  |
| K4A231 | Triticum urartu | | 48.58 | 4.295 | 0.025 | Calmodulin-related protein |
| A0A1D6RQN4 | Triticum aestivum | | 121.0 | 2.088 | 0.011 | Ran GTPase |
| A0A1D5WMA2 |  | | 89.73 | 3.711 | 0.034 | GTPase |
| **Stress/Defense** | | |  |  |  |  |
| M7ZQF1 | Triticum urartu | | 42.87 | 2.278 | 0.004 | Serpin-Z1C |
| A0A1D6D8Z1 | Triticum aestivum | | 15.53 | 2.826 | 0.002 | Alpha-amylase/trypsin inhibitor CM1 |
| B5B0D5 | Triticum aestivum | | 15.78 | 2.304 | 0.027 | Alpha-amylase/trypsin inhibitor CM16 |
| P81713 | Triticum aestivum | | 7.96 | 4.644 | 0.000 | Bowman-Birk type trypsin inhibitor |
| A0A1D5X8F8 | Triticum aestivum | | 15.27 | 4.167 | 0.048 | Trypsin inhibitor CMe |
| A0A1D6CG26 | Triticum aestivum | | 14.18 | 3.968 | 0.032 | Profilin |
| A3FKE5 | Triticum aestivum | | 19.32 | 0.408 | 0.000 | Superoxide dismutase |
| R7W2K4 | Aegilops tauschii | | 10.98 | 0.225 | 0.000 | Ozone-responsive stress-related protein |
| M7YWA0 | Triticum aestivum | | 76.12 | 0.050 | 0.002 | Heat shock 70 kDa protein |
| A0A1D6CM14 | Triticum aestivum | | 28.40 | 0.477 | 0.010 | Basic endochitinase C |
| M8A1S2 | Triticum urartu | | 16.47 | 0.301 | 0.019 | Trypsin/alpha-amylase inhibitor CMX1/CMX3 |
| **Nucleic acid-related** | | |  |  |  |  |
| A0A1D5Y5R8 | Triticum aestivum | | 16.11 | 4.574 | 0.014 | Glycine-rich RNA-binding protein |
| R7WDC3 | Aegilops tauschii | | 31.44 | 2.126 | 0.015 | 60S ribosomal protein L31 |
| M8AZD7 | Aegilops tauschii | | 63.77 | 2.362 | 0.017 | translation initiation factor 3 subunit E |
| A0A1D6B1C3 | Triticum aestivum | | 31.84 | 2.218 | 0.011 | translation initiation factor 3 subunit F |
| A3RCW1 | Triticum aestivum | | 49.03 | 2.401 | 0.031 | Translation initiation factor eIF5 |
| A0A1D5TKE5 | Triticum aestivum | | 23.39 | 2.388 | 0.037 | Translation elongation factor |
| A0A1D6S1Y9 | Triticum aestivum | | 17.15 | 0.304 | 0.002 | RNA binding |
| A0A1E5W0T3 | Sorghum | | 49.24 | 0.488 | 0.000 | Elongation factor 1-alpha |
| A0A1D5ZWW7 | Triticum aestivum | | 53.06 | 0.285 | 0.026 | Elongation factor Tu |
| M8BQP6 | Aegilops tauschii | | 30.81 | 0.393 | 0.041 | 60S ribosomal protein L10a-1 |
| M7YF02 | Triticum urartu | | 46.46 | 0.375 | 0.049 | 26S proteasome non-ATPase regulatory subunit RPN12A |
| **Protein synthesis/Assembly/Degradation** | | | | | |  |
| M7ZWM8 | Triticum urartu | | 39.07 | 2.961 | 0.004 | protein serine/threonine phosphatase |
| M7ZN83 | Triticum aestivum | | 43.58 | 0.371 | 0.002 | Protein disulfide isomerase-like 2-1 |
| P20973 | Triticum aestivum | | 117.01 | 0.290 | 0.046 | Ubiquitin-activating enzyme E1 1 |
| **Miscellaneous** | | |  |  |  |  |
| A0A1D5Z4H9 | Triticum aestivum | | 16.10 | 2.700 | 0.035 | actin binding |
| A0A1D6CWR8 |  | | 32.44 | 0.459 | 0.016 | methyltransferase |
| F2DVW0 | Hordeum vulgare | | 28.49 | 0.390 | 0.035 | peptidase |
| CON__P02533 | Triticum aestivum | | 51.62 | 0.417 | 0.038 | Keratin, type I cytoskeletal 14; |
| A0A1D5Z0I6 | Triticum aestivum | | 76.44 | 0.450 | 0.034 | formate-tetrahydrofolate ligase |
| A0A1D6DB09 | Triticum aestivum | | 44.30 | 0.401 | 0.012 | Fasciclin-like protein FLA5 |
| A0A1D5UG29 | Triticum aestivum | | 57.15 | 0.375 | 0.003 | methylmalonate-semialdehyde dehydrogenase |
| **Unknown** |  | |  |  |  |  |
| A0A1D5ZX81 | Triticum aestivum | | 43.71 | 3.287 | 0.005 |  |
| M8B8E6 | Aegilops tauschii | | 41.62 | 2.999 | 0.012 |  |
| A0A1D6CDM8 | Triticum aestivum | | 19.55 | 2.167 | 0.014 |  |
| A0A1D6DC72 | Triticum aestivum | | 22.55 | 2.355 | 0.004 |  |
| A0A1D5TPD5 | Triticum aestivum | | 122.8 | 3.102 | 0.020 |  |
| A0A1D5VJT5 | Triticum aestivum | | 32.63 | 2.224 | 0.030 |  |
| W5AUU7 | Triticum aestivum | | 33.69 | 2.449 | 0.045 |  |
| A0A1D6RUI3 | Triticum aestivum | | 24.32 | 0.384 | 0.003 |  |
| W5EST8 | Triticum aestivum | | 70.62 | 0.282 | 0.003 |  |
| A0A0D3G8F9 | Triticum aestivum | | 18.53 | 0.440 | 0.004 |  |
| A0A1D5VLL5 | Triticum aestivum | | 53.44 | 0.472 | 0.008 |  |
| A0A1D6DKE3 | Triticum aestivum | | 112.79 | 0.429 | 0.016 |  |
| A0A1D5RS02 | Triticum aestivum | | 52.32 | 0.447 | 0.017 |  |
| A0A1D6AQF5 | Triticum aestivum | | 44.68 | 0.183 | 0.018 |  |
| I1IEP9 | Triticum aestivum | | 17.85 | 0.335 | 0.023 |  |
| A0A1D5V5U4 | Triticum aestivum | | 23.31 | 0.318 | 0.032 |  |
| A0A1D5YGS5 | Triticum aestivum | | 31.27 | 0.414 | 0.034 |  |
| A0A096UT91 | Triticum aestivum | | 28.31 | 0.403 | 0.042 |  |

Ratio: Ratio of the abundance of the protein identified at YM49-198 to that ZM366.
