# Supplementary figures and images for: Quantitative analysis of the grain amyloplast proteome reveals differences in metabolism between two wheat cultivars at two stages of grain development
Source: BMC Genomics. 2018 Oct 24;19:768. doi: 10.1186/s12864-018-5174-z (PMC6201562; doi:10.1186/s12864-018-5174-z)

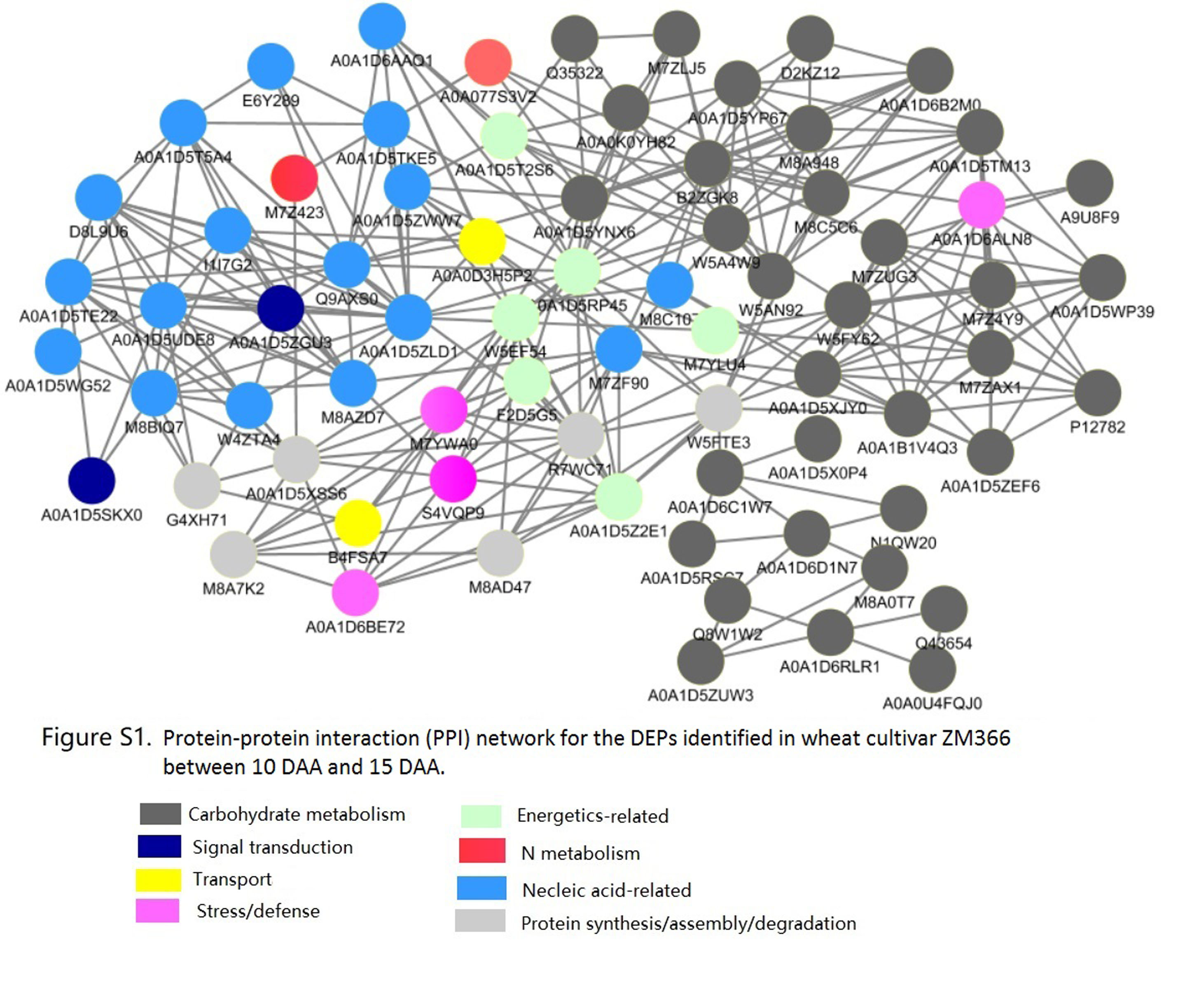

Supplement: Supplementary file 6 — Figure S1. Protein-protein interaction (PPI) network for the DEPs identified in wheat cultivar ZM366 between 10 DAA and 15 DAA. (TIF 4329 kb) [file 12864_2018_5174_MOESM6_ESM.tif]

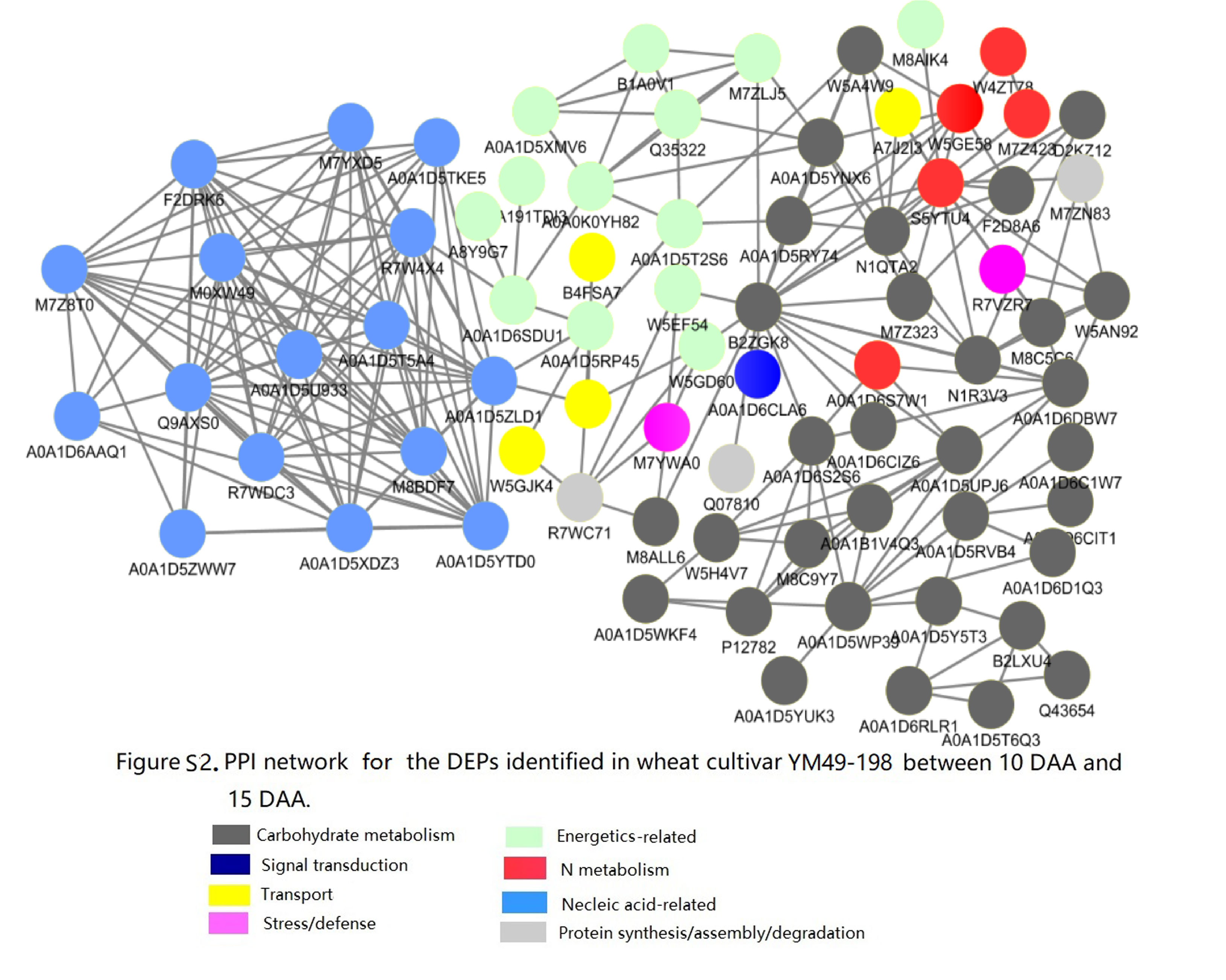

Supplement: Supplementary file 7 — Figure S2. PPI network for the DEPs identified in wheat cultivar YM49–198 between 10 DAA and 15 DAA. (TIF 3495 kb) [file 12864_2018_5174_MOESM7_ESM.tif]
